# Ohm’s law of electromagnetic ideal fluids: impedance-governed supercoupling in complex near-zero-index networks

**DOI:** 10.1038/s41467-026-73657-1

**Published:** 2026-06-02

**Authors:** Wendi Yan, Peihang Li, Jiarui Liu, Kaifeng Li, Pengyu Fu, Shuyu Wang, Mingzhe Hu, Yue Li

**Affiliations:** 1 Department of Electronic Engineering, Tsinghua University, Beijing, China; 2 Beijing National Research Center for Information Science and Technology, Beijing, China; 3 Department of Physics, Tsinghua University, Beijing, China; 4 State Key Laboratory of Space Network and Communications, Tsinghua University, Beijing, China

**Keywords:** Metamaterials, Nanophotonics and plasmonics, Electrical and electronic engineering

## Abstract

Supercoupling in near-zero-index (NZI) media enables geometry-insensitive electromagnetic (EM) transport through narrow channels with near-zero phase delay. However, most studies have focused on single-channel, point-to-point configurations, leaving EM power-flow distribution in complex structures largely unexplored. Here we extend NZI supercoupling to complex structures and show that EM power flow follows a passive, deterministic, and quasi-static distribution governed by boundary conditions and impedance contrasts, with PEC-terminated branches carrying no propagating power flow. We interpret this behavior using a pressure-driven flow analogy and directly visualize it in a waveguide-emulated plasmonic platform with photonic doping. This quasi-static power-flow distribution follows an “Ohm’s law of ideal EM power flow”, where the potential is set by boundary conditions and the effective impedance by each branch’s length-to-width ratio. Beyond the physical interpretation, our results suggest an impedance-designed approach to passive multi-port EM interconnects, offering insights for NZI physics and on-chip networks at millimeter-wave and terahertz frequencies.

## Abstract (web summary)

This study shows that electromagnetic energy in near-zero-index complex networks forms a quasi-static distribution following an “Ohm’s law”, where boundary conditions act as “potential differences” and branch length-to-width ratios act as “impedances”.

## Introduction

Supercoupling in near-zero-index (NZI) media represents a characteristic feature of electromagnetic (EM) wave propagation, in which energy becomes largely insensitive to geometric constraints^1–3^. Owing to near-zero permittivity or permeability, EM waves supported by NZI media exhibit negligible phase variation, enabling efficient energy tunneling through deeply subwavelength channels, sharp bends, and arbitrarily deformed waveguides^4–7^. Since its theoretical prediction and experimental verification, numerous unique optical and microwave devices based on the supercoupling effect have been developed, including impedance-matching and cloaking devices^8,9^, geometry-independent and wideband antennas^10–13^, flexible transmission lines^14^, advanced filters^15–17^, and even devices capable of performing calculus operations^18^. Despite these advances, existing studies on supercoupling have predominantly focused on isolated channels or individual functional devices, where energy transmission is only analyzed between predefined input and output ports. To extend supercoupling from point-to-point configurations to complex multi-branch structures, it is therefore important to investigate how EM power is distributed across interconnected NZI branches.

**Fig. 1:**
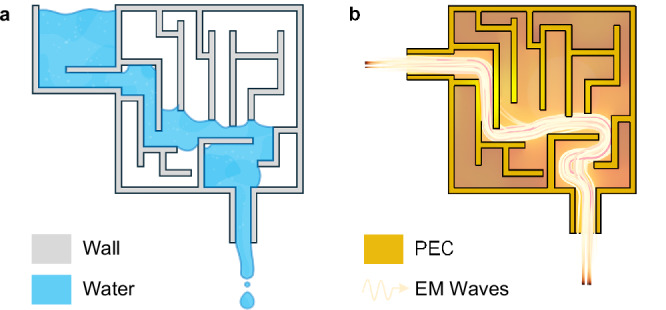
Conceptual sketch. **a** Conceptual sketch of water flowing through a complex branched channel network. **b** Electromagnetic analogue: quasi-static power flow distribution in a complex NZI network bounded by PEC walls.

Recently, EM power flow in NZI media has been shown to exhibit ideal-fluid-like behavior, thereby enabling EM energy distributions in complex NZI networks to be analyzed through a fluid-dynamical perspective^19,20^. In particular, pressure-driven water flow in a branched channel network provides a particularly intuitive analogy^21^. As demonstrated in Fig. 1a, when water is injected into a complex branched channel network, it propagates from the inlet to the outlet through a connected branch, while almost no water enters the blocked branches. This surprising behavior arises from pockets of air trapped in the blocked branches, which compress under pressure and push back against the incoming flow. Once the internal air pressure balances the hydrostatic force, further inflow into these branches is effectively suppressed^22–24^. (See Supplementary Note 1 for more details.) This observation suggests that, in branched fluidic networks, the steady-state flow distribution is passively determined by the inlet–outlet boundary conditions, the geometry of the branches, and the back-pressure generated at blocked terminations. Similar phenomena have also been observed in chemical wave propagation^25^, tube morphogenesis in amoeboid organisms^26^, and plasma path tracing^27^. Since EM waves in NZI media already exhibit ideal-fluid-like behavior^19,20^, power flow in complex NZI structures is expected to be governed by boundary conditions and impedance contrasts at terminations, thereby resulting in a passive and quasi-static field distribution.

In this work, we extend NZI supercoupling from isolated channels to complex, multi-branch EM networks, as shown in Fig. 1b, and systematically investigate how EM power is distributed through boundary-conditioned and impedance-governed transport. Specifically, we demonstrate that, in complex multi-branch PEC-defined structures filled with NZI media, EM power flow injected at an input port is passively distributed toward impedance-matched output ports, while power transport into PEC-terminated branches is strongly suppressed^19,20^. This multi-port supercoupling behavior is directly visualized in experiment using a waveguide-emulated plasmonic platform^28,29^ combined with photonic doping^14,15^. By contrast, in structures filled with vacuum, EM power flow exhibits strong sensitivity to geometry, leading to inefficient energy transport and negligible transmission to the output. Motivated by analogies with pressure-driven water flow in branched hydraulic networks^21^ and current distribution in direct-current circuits, we derive an Ohm’s law of ideal EM power flow^19,20^, which provides a simple framework for explaining the observed quasi-static power-flow distribution in NZI media. By appropriately engineering the boundary conditions and effective branch impedances in complex NZI structures, near-total power transmission can be achieved between desired input and output ports. This suggests an approach toward reconfigurable multi-port EM interconnects for future millimeter-wave (mmW) and terahertz (THz) systems^30–32^, where power delivery among multiple inputs and outputs can be controlled through terminal-impedance tuning. Overall, this work extends the supercoupling concept from point-to-point transmission to complex networks, experimentally demonstrates and directly visualizes this multi-port behavior, and suggests a possible reconfigurable EM interconnect for future millimeter-wave and terahertz systems.

## Results

### Simulation verifications

**Fig. 2:**
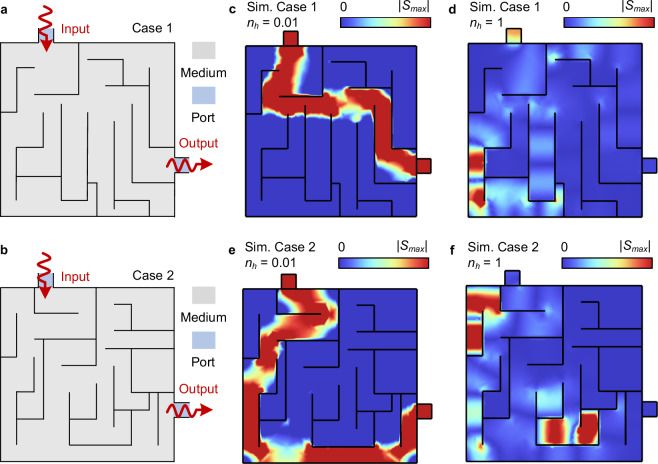
Simulated results of boundary-conditioned EM power redistribution in complex NZI networks. **a**, **b** Geometries of two randomly generated multi-branch structures, used to demonstrate the generality of the behavior. **c**, **e** Simulated magnitude of the real part of Poynting vector field in the structures. The simulated results in vacuum are demonstrated in **d**, **f** for comparison. Sim., Simulated.

To illustrate the generality of boundary-conditioned EM power-flow redistribution in NZI networks, we randomly generate two distinct multi-branch structures, as shown in Fig. 2a, b. Both structures are filled with media with refractive index *n*_h_, and two vacuum-filled ports are used for transmitting and receiving the waves (see Methods for more details about the simulations). The system is excited by transverse magnetic (TM) waves, with the electric field oriented in-plane and the magnetic field oriented out-of-plane. Figure 2c, e show the simulated magnitude of the real part of Poynting vector field (i.e., power flow) in the two structures filled with NZI media (*n*_h_ = 0.01) at a working frequency of 3 GHz. As shown, the power flow is predominantly concentrated in the impedance-favored branches connecting the input and output ports, whereas the PEC-terminated branches carry negligible net power flow. A reduced local power-flow magnitude is also observed in some regions, which can be attributed to the local widening of the branch, as will be further explained by the Ohm’s-law framework of ideal EM fluids introduced later. This behavior is qualitatively analogous to pressure-driven flow redistribution in fluidic networks, as illustrated in Fig. 1a and Supplementary Note 1. For comparison, the same structures filled with vacuum (*n*_h_ = 1) are simulated in Fig. 2d, f. In these cases, the Poynting vector exhibits an irregular spatial distribution, and there is negligible intensity at the output terminals—indicating that EM power transport through the structure is strongly suppressed. Quantitatively, the transmission efficiencies for Fig. 2d, f are only 15% and 12%, respectively, whereas for Fig. 2c, e, the efficiencies approach nearly 100% (see Fig. S1). Simulated time-domain results can be found in Movie S1, where this boundary-conditioned redistribution is observed with enhanced clarity. This behavior will be experimentally verified and directly visualized in the next section.

### Experimental visualization

**Fig. 3:**
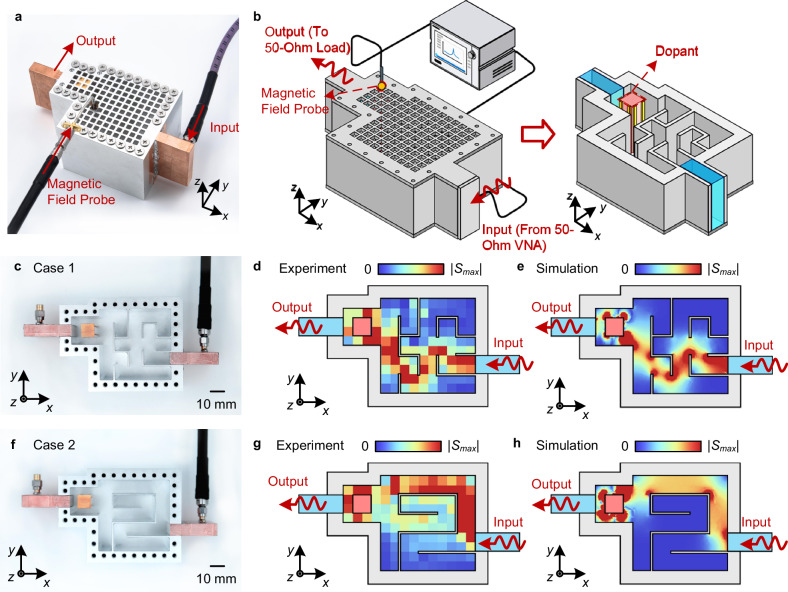
Experimental visualization. **a** Photograph of the experimental setup. **b** Schematic of the experimental design. **c**–**h** Top view of the experimental setup (with the cover removed), along with experimental and simulated power-flow distributions for two different experiments. **d**, **g** experimental results. **e**, **h** simulated results.

To experimentally verify boundary-conditioned EM power redistribution in NZI networks, a low-loss epsilon-and-mu near zero (EMNZ) material with a permittivity *ε*_r_ ~ 0 and a permeability *μ*_r_ ~ 0 is required. Plasmonic NZI materials operating in the optical or terahertz band are limited by their intrinsic losses^33,34^. Here, motivated by waveguide-emulated plasmonics^29^ and the concept of photonic doping^14,15^, we apply a parallel-plate waveguide incorporating a dielectric dopant to construct a low-loss EMNZ medium, whose photograph and schematic are depicted in Fig. 3a, b, respectively. As predicted by waveguide-emulated plasmonics, the central cross-section of a waveguide excited in the Transverse Electric (TE)_10_ mode possesses an effective relative permittivity given by *ε*_h_ = 1 – (*λ*/2 *h*)^2^. Accordingly, a waveguide with height *h* = *λ*/2 can emulate an effectively lossless epsilon-near-zero (ENZ) medium, yielding an approximate condition of *ε*_h_ ~ 0 within a finite bandwidth in our setup^28,29^. In addition, photonic doping offers a powerful mechanism for tailoring the effective permeability in ENZ media. Benefiting from the intrinsic field-enhancement effect of ENZ environments, embedding a square dielectric inclusion within the ENZ background enables controlled modification of the effective permeability, which can be expressed as^7,14^:

1 $${\mu }_{{{\rm{eff}}}}=1+\frac{64{l}_{{{\rm{d}}}}^{2}}{{\pi }^{4}A}\frac{{\omega }^{2}}{{{\omega }_{{{\rm{pmc}}}}}^{2}-{\omega }^{2}},\,{\omega }_{{{\rm{pmc}}}}=\frac{c}{\sqrt{{\varepsilon }_{{{\rm{d}}}}}}\sqrt{2{(\pi /{l}_{{\rm{d}}})}^{2}}$$

where *A* is the area of the ENZ background, and *l*_d_ is the size of the square dopant. The frequency dependence of the effective *μ*_eff_ is shown in Fig. S9 according to Eq. (1). When the area of the ENZ host is much larger than that of the dopant, the interior of the composite system can be approximately treated as having a uniform effective permeability distribution. A metal shorting strip, placed parallel to the wide side of the waveguide on the dopant surface, is used to suppress non-TE_10_ modes. Therefore, by precisely designing the size of the dopant, *μ*_eff_ ~ 0 can also be achieved, thus EMNZ can be simultaneously achieved at the cutoff frequency. We place multiple metallic boundaries inside the waveguide to construct a complex multi-branch network. The method of direct observation of EM fields is employed to enable accurate measurement of the Poynting vector on the central cross-section of the waveguide^20^. Specifically, the top plate of the waveguide is replaced with a metallic mesh composed of 116 grid cells while maintaining its original performance. A magnetic field probe is used to measure the magnetic field in these grids on the top surface of the waveguide, which can be used to extract the relative magnitude of the power flow (see Methods for the analysis of direct observation of EM fields).

To demonstrate the generality of the boundary-conditioned redistribution behavior in NZI media, we design two different structures, whose top-view of photographs are provided in Fig. 3c, f. The power flow, i.e., the real part of the Poynting vector extracted from the experiment is demonstrated in Fig. 3d, g. For comparison, simulated power flows of these two cases are depicted in Fig. 3e, h, respectively. As evidenced by the experimental results, in both scenarios, a dominant power-flow distribution connecting the input and output ports is clearly observed. Along the mismatched branches, the power-flow magnitude is significantly suppressed, dropping to less than half of that in the impedance-matched branches. These measurements remain highly consistent with the simulated results, and minor discrepancies may arise from fabrication tolerances and a slight frequency shift due to deviations in material parameters. Moreover, despite the varying path lengths and geometric complexities, both structures support near-total transmission from input to output (see Fig. S2), demonstrating the geometry-insensitive feature^10,30^. Additionally, a higher-resolution experimental result is presented in Fig. S12, which more solidly demonstrates the correspondence between the theoretical prediction and physical reality. However, it should be emphasized that this phenomenon arises only under carefully engineered ENZ/EMNZ conditions and is not representative of general EM propagation. In the present platform, because both the permittivity and permeability are dispersive, this effect can only be observed in a narrowband structure. In fact, the corresponding bandwidth is about 7%, as shown in Fig. S9. In summary, these experimental results, together with simulation results in Fig. 2, verify and directly visualize the generality of the observed multi-port power-flow distribution in NZI media, which is explained in the next section using a theoretical Ohm’s-law of ideal EM fluids.

### Theoretical analysis

**Fig. 4:**
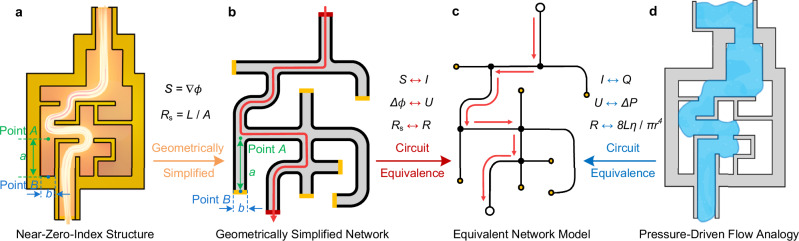
Theoretical analysis. **a** Schematic of a complex NZI structure. **b** Geometrically simplified representation of the structure in (**a**). **c** Equivalent circuit network representation of the simplified network in (**b**). **d** Pressure-driven fluidic network presented as an intuitive analogy to the circuit network shown in (**c**).

In this section, we provide an effective theoretical description of power redistribution in complex NZI structures. We first map the complex multi-branch geometry onto an equivalent network under the ideal NZI limit. Considering a branch with length *a* and width *b* in an ideal complex NZI structure (*ε*_h_ = 0) depicted in Fig. 4a, Faraday’s law can be applied^30,35^, and we obtain

2 $$\oint _{\partial A}{{\bf{E}}}\cdot d{{\bf{l}}}=-\iint _{A}\frac{\partial {{\bf{B}}}}{\partial t}\cdot d{{\bf{A}}}=i\omega {\mu }_{{{\rm{h}}}}{H}_{0}ab$$

Here, *μ*_h_ is the permeability of ENZ media, and *H*_0_ is the uniform magnetic field out-of-plane. According to Eq. (2), under the ideal NZI limit at a fixed operating frequency, the magnetic field *H*_0_ is primarily determined by the branch boundary conditions and its cross-section area, while being weakly sensitive to detailed geometrical deformations. This mechanism exemplifies the spatiotemporal decoupling characteristic inherent in NZI media, which has enabled numerous deformable NZI-based devices^10,30,36^. In other words, as long as the branch length *a* and width *b* remain unchanged, these segments can be stretched, allowing the complex NZI structure shown in Fig. 4a to be represented as an equivalent multi-port waveguide network, as illustrated in Fig. 4b. Within this perspective, Fig. 4b reveals that the complex structure effectively operates as a network of coupled NZI supercoupling channels, thereby extending the conventional point-to-point supercoupling paradigm to a multi-branch, system-level configuration.

In Fig. 4b, a branch that terminates at an output port corresponds to an impedance-matched transport branch, whereas a branch terminated by a PEC represents a strongly mismatched termination. According to the irrotational property of ideal EMNZ limit ∇ × **S** = 0^19^, the time-averaged Poynting vector is approximately curl-free, i.e.,

3 $${{\bf{S}}}=\nabla \phi$$

where *ϕ* denotes the velocity potential of the Poynting vector^19^. Applying Green’s theorem to the branch, we obtain

4 $${\oint }_{\partial A}\frac{\partial \phi }{\partial n}dl={\iint }_{A}{\nabla }^{2}\phi dS=0$$

where *dl* is the length element and *dS* is the area element. In the second equality in Eq. (4), we employ the incompressibility assumption of EM fields in ideal EMNZ media, ∇^2^*ϕ* = ∇ ∙ **S** = 0^19^. In other words, within the ideal EMNZ approximation (*ε*_h_ = 0, *μ*_h_ = 0), the Poynting vector satisfies the conditions of a harmonic field inside NZI media. For the non-ideal cases, please refer to Supplementary Note 4.

For a branch terminated by a PEC, all boundaries are PECs except at the branching junction, where the Neumann boundary condition ∂*ϕ*/∂*n* = 0 applies. Since the expected flow direction is normal to the junction at point *A* when the port width is relatively narrow (less than *λ*/2), we have

5 $${\int }_{a}\frac{\partial \phi }{\partial n}dl=0\to \frac{\partial \phi }{\partial n}=0\,{{\rm{at}}}\,A$$

Hence, the entrance to a PEC-terminated branch effectively behaves as a virtual PEC boundary, strongly suppressing net power flow into that branch. Furthermore, for a branch starting at point *A* and ending at point *B*, we have:

6 $${\int }_{a}{\int }_{b}{S}dxdy={\phi }_{{\rm{B}}}-{\phi }_{{\rm{A}}}\to b{\int }_{a}{S}dy={\phi }_{{\rm{B}}}-{\phi }_{{\rm{A}}}$$

Here, because the port width is very narrow, the Poynting vector along the port direction can be assumed to be approximately uniform. Assuming the power flow along each branch is approximately uniform under ideal or near-ideal EMNZ conditions, we can write *R*_S_ = *a* /*b*, and Δ*ϕ* = *ϕ*_B_ - *ϕ*_A_, and Eq. (6) can be written as

7 $${{SR}}_{{\rm{S}}}=\Delta {{\phi}}$$

This expression closely resembles Ohm’s law in direct current (DC) circuits^37^ and is therefore termed the Ohm’s law of ideal EM power flow. According to Reference ^21^, a formally similar relationship appears in pressure-driven water flow networks, described by:

8 $$\Delta P=Q\,8a\eta /\pi {r}^{4}$$

Here, *Q* denotes the volumetric flow rate in an ideal fluid, Δ*P* is the pressure difference between the inlet and the outlet, 8*aη*/*πr*^*4*^ is the water resistor, *a* is the length of the channel, *η* is the dynamic viscosity of ideal fluid, and *r* is the width of the ideal fluid branch. Within this analogy, a mathematical correspondence can be drawn among ideal EM power flow, DC circuits, and ideal fluid flow:

9 $$\begin{array}{l}{{\rm{Ideal}}}\,{{\rm{EM}}}\,{{\rm{power}}}\leftrightarrow {{\rm{DC}}}\,{{\rm{circuit}}}\leftrightarrow {{\rm{Ideal}}}\,{{\rm{fluid}}}\\ {{S}}\,\leftrightarrow {{\rm{Current}}}\,I\leftrightarrow Q\\ \Delta \phi\, \leftrightarrow {{\rm{Voltage}}}\,U\leftrightarrow \Delta P\\ {R}_{{\rm{S}}}=a/b\,\leftrightarrow {{\rm{Resistance}}}\,R\leftrightarrow 8a\eta /\pi {r}^{4}\end{array}$$

This explains why the ideal EM power flow exhibits a mechanism analogous to that of water flow. In other words, these quasi-static power-flow distributions are governed by Poynting potential difference Δ*ϕ* in ideal EM power flow, by voltage *U* in DC circuits, and by pressure difference Δ*P* in ideal fluid flow. As shown in Fig. 4b, the Poynting potential at impedance-matched branches is high, whereas the potential at PEC-terminated ports is effectively pinned to zero (note that this sign convention differs from that of the electric potential by a minus sign). Consequently, EM power is passively redistributed toward impedance-matched output ports, while power flow into strongly mismatched branches is suppressed. Moreover, the effective impedance of each branch is set by its geometry, with *R*_S_ ~ a/b. As a result, the power-flow distribution is passively and deterministically governed by the boundary conditions and impedance contrasts across the network, in agreement with the simulated results in Fig. 2, and as further illustrated in Figs. S10 and S11. In addition, although the above theoretical derivation was carried out for the EMNZ case, the underlying mechanism is in fact highly robust against variations in permeability. It therefore applies to a broad range of ENZ implementations, including the waveguide-emulated plasmonic used in our experiments. However, for conventional media, where the assumptions underlying ideal EM fluids no longer hold, the Ohm’s-law description is generally no longer applicable. A detailed discussion can be found in Supplementary Note 4. This framework provides an intuitive and physically consistent description of boundary-conditioned EM power redistribution in NZI networks.

### Multi-port interconnect applications enabled by supercoupling

**Fig. 5:**
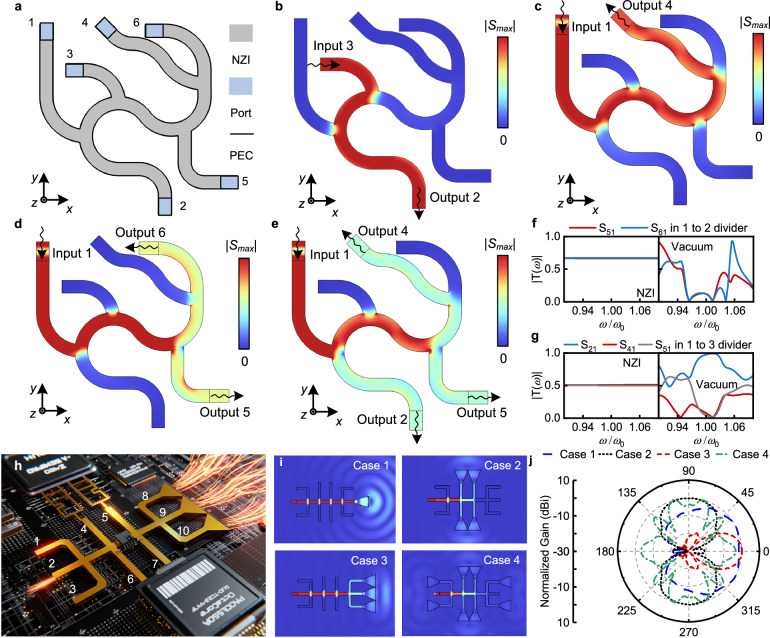
Multi-port interconnect applications enabled by multi-branch supercoupling. **a** Conceptual illustration of a multi-port supercoupling network. The network is filled with NZI media and is equipped with six ports. **b**, **c** Simulated magnitude of the real part of the Poynting vector field in a multi-port supercoupling network for different input-output configurations, respectively. **d**, **e** Simulated magnitude of the real part of Poynting vector field with two outputs or three outputs, respectively. These designs can be applied to 1-to-2 or 1-to-3 divider. **f**, **g** Simulated transmission amplitude in (**d**, **e**). For comparison, transmission amplitudes in vacuum cases are also demonstrated. **h** Conceptual sketch of a multi-port supercoupling bus at millimeter-wave and terahertz frequencies. The bus features ten ports that can interface with different EM components. **i** The simulated electric field distributions of the bus with different integrated horns for four representative cases. **j** Corresponding simulated radiation patterns for 4 different cases in (**i**).

Inspired by the above Ohm’s-law framework, the EM power-flow distribution in such systems is determined by the boundary potentials and the effective branch impedances set by *R*_S_ ~ a/b, thereby enabling passive control of EM power redistribution through structural design. Here, we demonstrate a multi-port NZI interconnect network, as illustrated in Fig. 5a. Specifically, it consists of a waveguide with irregular shape. The waveguide is filled with NZI material and equipped with multiple distinct input and output ports. By engineering the Poynting potential at the ports (i.e., either impedance-matched ports or PEC-terminated branches), the overall power flow can be redistributed, enabling near-total power transmission between desired ports. Two different examples are shown in Fig. 5b, c. In these cases, EM power is predominantly concentrated in specific branches of the network, while the field intensity in the remaining branches is strongly reduced. Moreover, multiple output ports can be simultaneously activated. When the output ports have equal *R*_S_, the input power is evenly split among them^36^, as demonstrated in Fig. 5d, e. In Fig. 5d, the power is equally divided between Port 5 and Port 6. In Fig. 5e, the power is split into three equal portions and delivered to Ports 2, 4, and 5. The corresponding transmission amplitudes are shown in Fig. 5f, g, with additional reference to the results in a vacuum-filled structure for comparison. We can also control the power distribution among the output ports by adjusting their widths, effectively tuning the impedance *R*_s_ associated with the power flow at each port, as illustrated in Fig. S10. In the presence of multiple effective branches, the power distribution between the two channels also follows Eq. (9), as illustrated in Fig. S11. These results show that the Ohm’s-law framework enables predictable and designable multi-port power redistribution in complex NZI structures through structural control of the effective impedances.

Furthermore, the reconfigurable multi-port power redistribution enabled by multi-branch supercoupling suggests potential applications in multi-port EM interconnects. Inspired by the “bus” concept widely used in electronic and photonic systems^38^, we introduce an EM interconnect device based on multi-branch supercoupling for waveguide circuits at mmW and THz frequencies. The conceptual prototype is illustrated in Fig. 5h, featuring ten ports designed to interconnect various functional devices, including representative examples such as waveguide feeding structures (Ports 1-3), Wilkinson power dividers (Ports 4 and 5), analog computing units (Ports 6 and 7), and horn antennas (Ports 8-10). Taking antennas as an example, such an interconnect could enable reconfigurable coupling among a variable number of elements to form arrays for wavefront shaping. Figure 5i exhibits the simulated electric field distributions for four different cases: a single antenna, a four-element antenna array, a three-element antenna array, and an eight-element antenna array. These results clearly demonstrate the system’s capacity to manipulate the radiated wavefront with high precision. Corresponding normalized radiation patterns are plotted in Fig. 5j. Furthermore, an example of a filter–divider interconnection is provided in the Supplementary Note 3. Conventional interconnect schemes for mmW and THz devices suffer from considerable transmission loss at high frequencies^39,40^ and pose significant challenges for unified on-chip integration due to mismatched device scales^41,42^. Unlike previous studies that focus on point-to-point supercoupling^4–7^, this device extends the paradigm to multi-port power redistribution governed by an Ohm’s law of ideal EM fluids. Besides, unlike guided-wave-driven metasurfaces, which focus on wavefront shaping and radiation, the proposed EMNZ bus is designed for passive interconnection of EM power^43^.

## Discussion

In this work, we extend NZI supercoupling from conventional point-to-point transport to complex multi-branch structures, and investigate the resulting quasi-static EM power-flow redistribution through an analogy to pressure-driven flow in fluidic networks. Specifically, EM power injected into complex multi-branch NZI structures is passively redistributed toward impedance-matched output ports, while power flow into strongly mismatched or PEC-terminated branches is strongly suppressed. Both simulations and experiments verify and directly visualize this multi-branch power-flow distribution. The behavior can be described by an Ohm’s law of ideal EM fluids, in which the power-flow intensity is determined by the Poynting-vector potential difference between ports and the effective impedance, which is set by the length-to-width ratio, thus providing a predictive description of passive and deterministic EM power redistribution in complex NZI structures. Building upon this mechanism, we show that by engineering the branch geometry and terminal conditions, supercoupling can be extended from isolated channels to multi-port NZI networks, thereby enabling a conceptual waveguide-based mmWave/THz bus-like EM interconnect for multi-port signal or power distribution through a shared NZI platform. Our findings not only offer new perspectives on supercoupling and quasi-static EM power-flow distribution in NZI media, but also suggest a potential pathway toward interconnection in complex photonic and microwave networks, including mmWave/THz integrated architectures.

## Methods

### Direct observation of electromagnetic fields

The measured results demonstrated in Fig. 3 are obtained according to the method of direct observation of EM fields^20^. As predicted by waveguide-emulated plasmonics, the central cross-section of a waveguide operating at the cutoff frequency of the TE_10_ mode possesses an effective permittivity *ε*_h_ ~ 0. Since the structure is axially symmetric with respect to the central cross-section (*z* = 0, where *z* represents the position of the field), the electric field in the waveguide can be expanded as a Fourier series that contains only the first order of cosine term according to the electric distribution of TE_10_ mode:

10 $${\mathbf {E}}(z)=\,\cos (\frac{\pi z}{h}){\mathbf {E}}(z=0)$$

where **E**(z = 0) is the vector electric field at the central cross-section without z components, and *h* is the height of the waveguide. Applying Faraday’s law ∇ × **E** = *iωμ***H** in Eq. (10), the magnetic field within the waveguide can be calculated as

11 $${\mathbf {H}}(z)=\frac{1}{i\omega {\mu }_{0}}\,\cos (\frac{\pi z}{h})(\nabla \times {\mathbf {E}}(z=0))-\frac{\pi }{i\omega {\mu }_{0}h}\,\sin (\frac{\pi z}{h}){\widehat{z}} \times {{\mathbf{E}}}(z=0)$$

On the top surface of the waveguide *z* = *h*/2, only the second term is retained, and we obtain

12 $${\mathbf {E}}(z=0)=\frac{i\omega {\mu }_{0}h}{\pi }{\mathbf {H}}(z=\frac{h}{2}) \times {\widehat{z}}$$

Consequently, the Poynting vector at the central cross-section of the waveguide can be written as

13 $${\mathbf {S}}(z=0)={\mathbf {E}}(z=0)\times {\mathbf {H}}\left(z=0\right)=\frac{i\omega {\mu }_{0}h}{\pi }{{\mathbf{{H}}}}(z=\frac{h}{2}) \times {\widehat{z}} \times {{\mathbf{H}}}(z=0)$$

For the TE_10_ mode, the magnetic fields only remain the *z*-component, i.e., **H**(*z* = 0) = *H*(*z* = 0) ẑ, the Poynting vector can be calculated as

14 $${{\mathbf{S}}}(z=0)=	 -\frac{i \omega {\mu }_{0}h}{\pi }{H}(z=0)\left[{{\mathbf{H}}}\left(z=\frac{h}{2}\right)(\hat{z}\cdot \hat{z})-\hat{z}\left(\hat{z}\cdot {{\mathbf{H}}}\left(z=\frac{h}{2}\right)\right)\right] \\=	 -\frac{i\omega {\mu }_{0}h}{\pi }{H}\left(z=0\right){{\mathbf{H}}}\left(z=\frac{h}{2}\right)$$

In the second equality, the orthogonality of **H**(*z* = h/2) and ẑ is utilized. Near the cutoff frequency, *H*(*z* = 0) is approximately uniform, meaning the Poynting vector at the central cross-section of the waveguide **S**(*z* = 0) can be approximately proportional to the relative value of the magnetic field measurement **H**(*z* = h/2) to the top surface, i.e.,

15 $${\mathrm{Re}}({\mathbf {S}}(z=0)) \propto {\mathrm{Re}}\left({\mathbf {H}}(z=\frac{h}{2})\right)$$

Therefore, to measure the power flow on the effective *ε*_r_ ~ 0 plane (i.e., the central cross-section of the waveguide), only the magnetic field on the top surface needs to be measured. Specifically, we develop an electrically small metal loop connected to a coaxial cable as a magnetic field probe to measure the complex-valued tangential components of the magnetic fields on the top surface, as demonstrated in Fig. 3b. When measuring the magnetic fields, the output port is connected to a 50-Ohm load for absorption and a vector network analyzer is used to measure the transmission coefficient from the input port to the probe. This coefficient corresponds to the relative amplitude and phase of **H**(*z* = h/2) at the top surface. Since the magnitude of this transmission coefficient is usually lower than −20 dB, indicating that no more than 1% of the power is coupled to the probe, it can be observed that interference with the field inside the cavity is small enough to be neglected.

### Full-wave simulation

The two-dimensional (2D) simulation results in Figs. 2c–f, 5b–g, i, j are conducted by the frequency-domain RF module of the finite-element-method (FEM) commercial software COMSOL Multiphysics^®^ v5.0 (available at www.comsol.com) in 2D cases. In these simulations, the boundaries of the structures are set as perfect electric conductors (PECs). The inputs are set as rectangular ports excited by transverse electromagnetic (TEM) wave. Specifically, the magnetic field is out-of-plane, while the electric field is in-plane. The specific dimensions could be determined by scaling measurements from the geometric drawing. The three-dimensional (3D) simulation results in Fig. 3e, h are performed using the ANSYS HFSS^®^. The simulated structures and materials are designed to be the same as that of experimental setups, which can be obtained in the next section.

### Experiment setup

The prototypes of the experiment setups presented in Fig. 3a, c, f are fabricated using the computer numerical control (CNC) techniques. The waveguides are made of aluminum metal alloy, with other metals sprayed on the surface to prevent oxidation. The top plate of the waveguide is replaced with a metallic mesh composed of 116 grids (i.e., a 10 × 10 mesh plus a 4 × 4 mesh) to facilitate the insertion of the magnetic field probe for measuring the relative magnetic field amplitude and phase, as illustrated in Fig. 3. Since the side length of the slot (4 mm × 4 mm) is much smaller than the operating wavelength (*λ* = 98.6 mm, which corresponds to *ω*_p_ = 2*π* × 3.04 × 10^9^ rad/s in our experiment setup), this structure can prevent EM wave leakage, ensuring the same performance as the intact upper surface. The height of the waveguide is set to *h* = 49.3 mm. A dielectric rod made of dielectric ceramics with relative permittivity *ε*_d_ = 37.8 + *i*0.0038 has dimensions of 11.5 mm × 11.5 mm and located in the waveguide to act as a dopant. This level of loss ensures that the ideal EM fluid behavior can be maintained in the experiment. According to the photonic doping theory^28,29^, the waveguide-emulated plasmonics has an effective permittivity *ε*_r_ ~ 0 and an effective permeability as follows:

16 $${\mu }_{{{\rm{eff}}}}(\omega )\approx 1+\frac{64{{l}_{d}}^{2}}{{\pi }^{4}A}\frac{{\omega }^{2}}{{{\omega }_{{{\rm{pmc}}}}}^{2}-{\omega }^{2}},\,{\omega }_{{{\rm{pmc}}}}=\frac{c}{\sqrt{{\varepsilon }_{{\rm{d}}}}}\sqrt{2{(\pi /{l}_{{\rm{d}}})}^{2}}$$

where *l*_d_ = 11.5 mm is the side length of the dopant, *A* = 3848 mm^2^ is the area of the structure, *ω* is the angular frequency, and *ε*_d_ = 37.8 + *i*0.0038 is the relative permittivity of the dopant. Under the parameter settings, the device exhibits near-zero permeability *μ*_d_ ~ 0 around *ω* = 2*π* × 3.04  × 10^9^ rad/s. In other words, at the experimentally tested frequency point, the waveguide-emulated plasmonics simultaneously achieves near-zero permittivity and permeability, corresponding to the NZI state. The magnetic field probe of the experimental setup has a radius of 1.5 mm and is connected to one of the ports of the vector network analyzer Agilent N9917A via a 50 Ω coaxial line. The other port is connected to the input waveguide to excite the input EM waves. The probe and mesh top plate are shown to exert a negligible impact on the fields within the structure, as illustrated in Fig. S3.

## Supplementary information

Supplementary Information

Description Of Additional Supplementary File

Supplementary Data 1

Supplementary Data 2

Supplementary Data 3

Supplementary Movie 1

Transparent Peer Review file

## Data Availability

The experimental data supporting the findings of this study are provided in Supplementary Data 1 and 2. The remaining data are provided as compressed files in Supplementary Data 3.
