# Supplementary material for: Ohm’s law of electromagnetic ideal fluids: impedance-governed supercoupling in complex near-zero-index networks
Source: Nat Commun. 2026 Jun 2;17:7131. doi: 10.1038/s41467-026-73657-1 (PMC13396760; doi:10.1038/s41467-026-73657-1)
Supplement: Supplementary file 1 — Supplementary Information [file 41467_2026_73657_MOESM1_ESM.pdf]

Supplementary Information for

Ohm's Law of Electromagnetic Ideal Fluids: Impedance-Governed Supercoupling in Complex Near-Zero-Index Networks

Wendi Yan, Peihang Li, Jiarui Liu, Kaifeng Li, Pengyu Fu, Shuyu Wang, Mingzhe Hu, and Yue Li*

Corresponding author: lyee@tsinghua.edu.cn

The PDF file includes:

Supplementary Fig. 1 to Supplementary Fig. 12

Supplementary Note 1 to Supplementary Note 4

Other Supplementary materials for this manuscript include the following:

Supplementary Movie 1

Supplementary Data 1 to Supplementary Data 3

Supplementary Figures

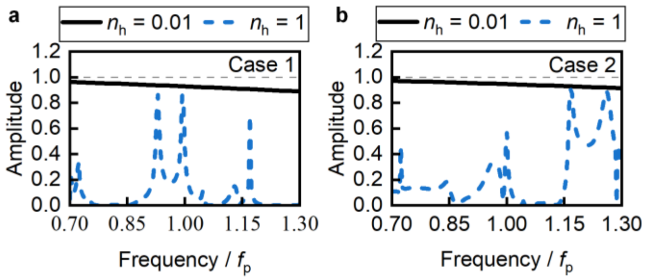

Supplementary Fig. S1 | Simulated transmission amplitude of supercoupling in complex NZI networks, which is demonstrated in Fig. 2 in the main text. a Case 1. **b** Case 2. The central frequency f_p is set as f_p = 3GHz

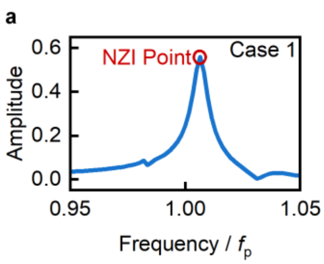

Supplementary Fig. S2 | Measured transmission amplitude of supercoupling in complex NZI networks in Fig. 3 in the main text. a Case 1. **b** Case 2. Both measured frequencies are near the central frequency f_p = 3.04 GHz (see Materials and methods).

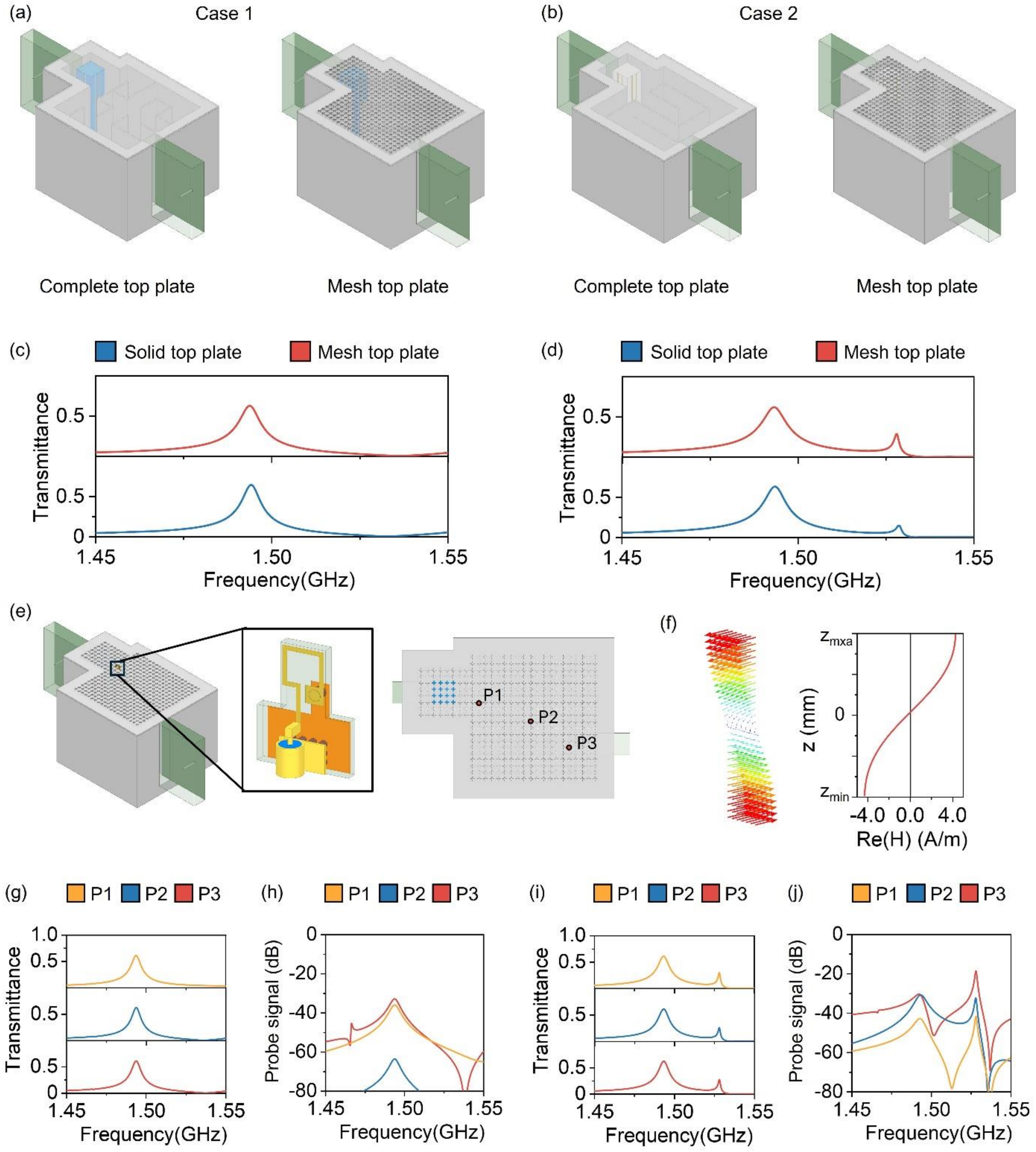

Supplementary Fig. S3 | Effect of test scenarios on waveguide EMNZ fields. a–b Schematic diagrams of the two configurations with a grid top plate and a solid top plate. **c** Influence of different top plates on the transmission spectrum for Case 1. (d) Influence of different top plates on the transmission spectrum for Case 2. **e** Schematic of probe signal detection, with three probe locations labeled P1, P2, and P3. **f** Schematic diagram of magnetic field distribution in the cross-section of the structure. **g** Transmission spectra of the Case 1 system for probes at P1, P2, and P3. **h** Measured probe signals at P1, P2, and P3 in Case 1. **i** Transmission spectra of the Case 2 system for probes at P1, P2, and P3. **j** Measured probe signals at P1, P2, and P3 in Case 2.

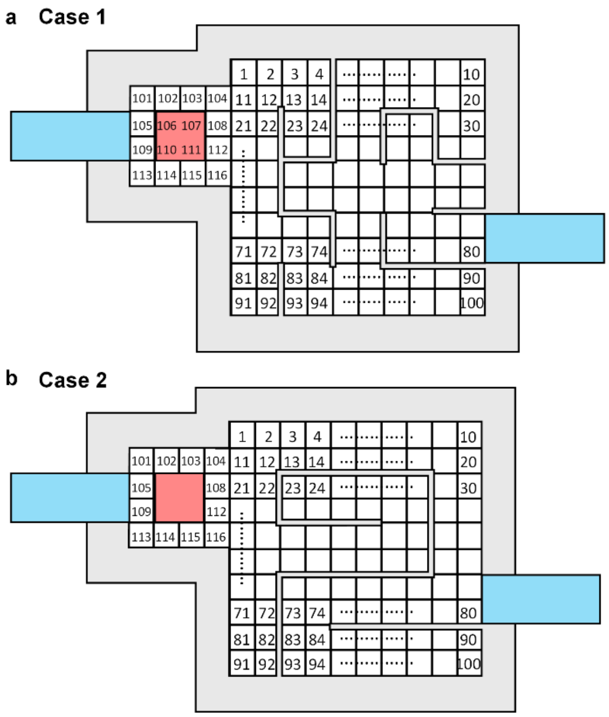

Supplementary Fig. S4 | The specific position numbers corresponding to measured locations. a Case 1. **b** Case 2. For detailed measured results, please refer to Supplementary Data 1.

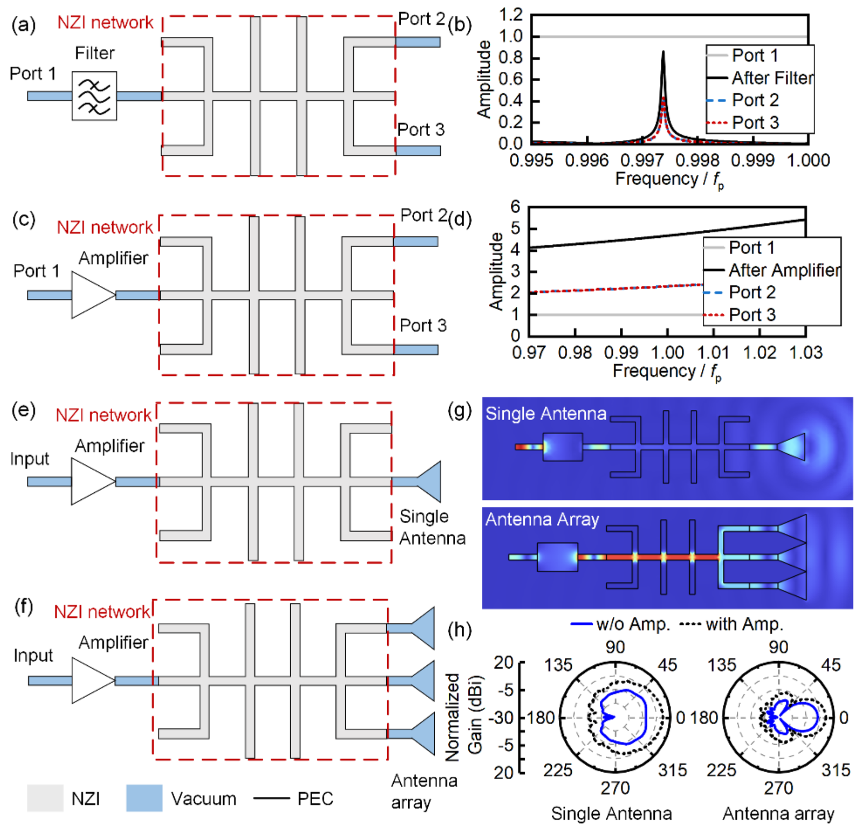

Supplementary Fig. S5 | Other examples of applications from NZI network in millimeter and terahertz. a Schematic of connecting the NZI network with filter. **b** Simulated transmission amplitude in (a). **c** Schematic of connecting the NZI network with amplifier. (d) Simulated transmission amplitude in (c). **e** and (f) Schematic of connecting the NZI network with amplifiers and antennas. In (e), the NZI network is connected with a single antenna. In (f), the NZI network is connected with an antenna array. **g** Simulated electric field distributions for examples in (e) and (f). **h** Simulated normalized gain for (e) and (f), together with the same cases without amplifier. w/o., without. Amp., amplifier. The central frequencies of all simulation are set as f_p = 60 GHz.

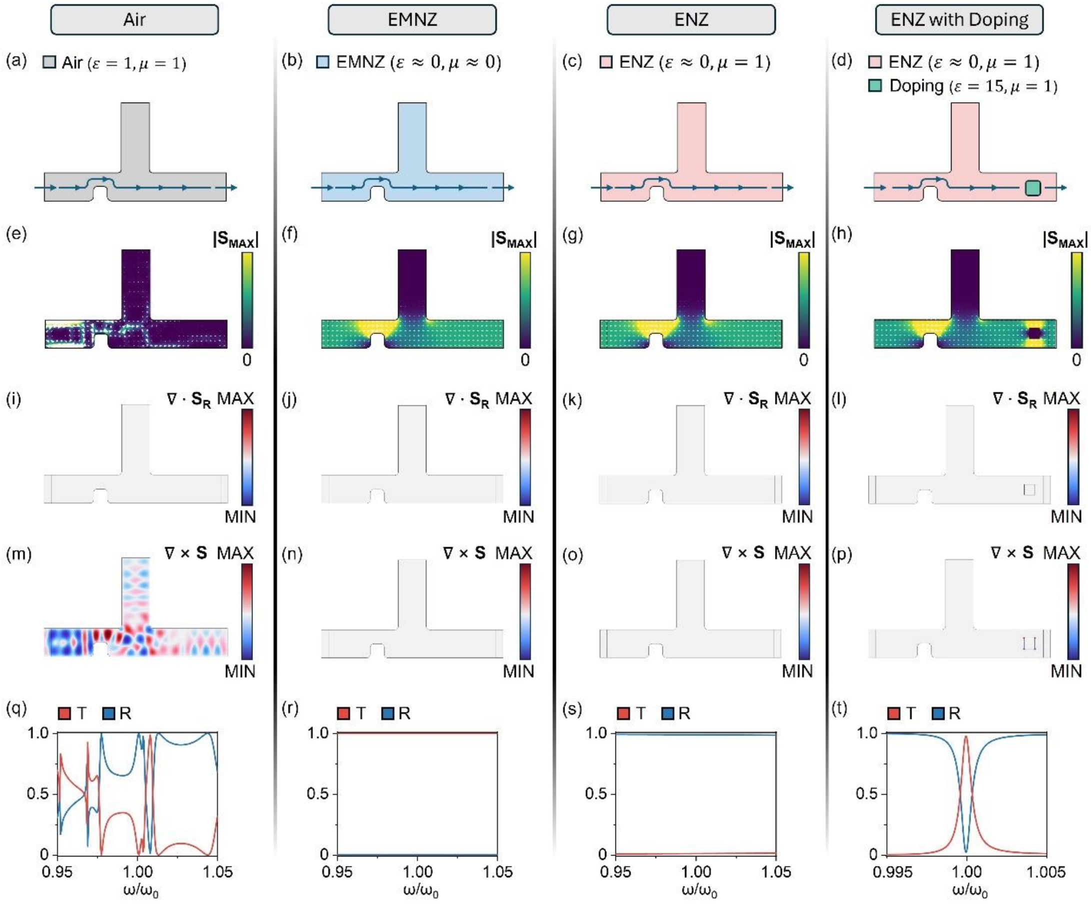

Supplementary Fig. S6 | Power-flow distribution of electromagnetic waves in different media. a–d Schematic diagrams of the structures with air, EMNZ, ENZ, and ENZ with doping, respectively. **e–h** Corresponding power-flow distributions in each medium, where the white arrows indicate the direction of power flow. **i–l** Distributions of the real part of the power-flow divergence for each medium. **m–p** Distributions of the real part of the power-flow curl for each medium. **q–t** Transmission spectra of electromagnetic waves in the four media.

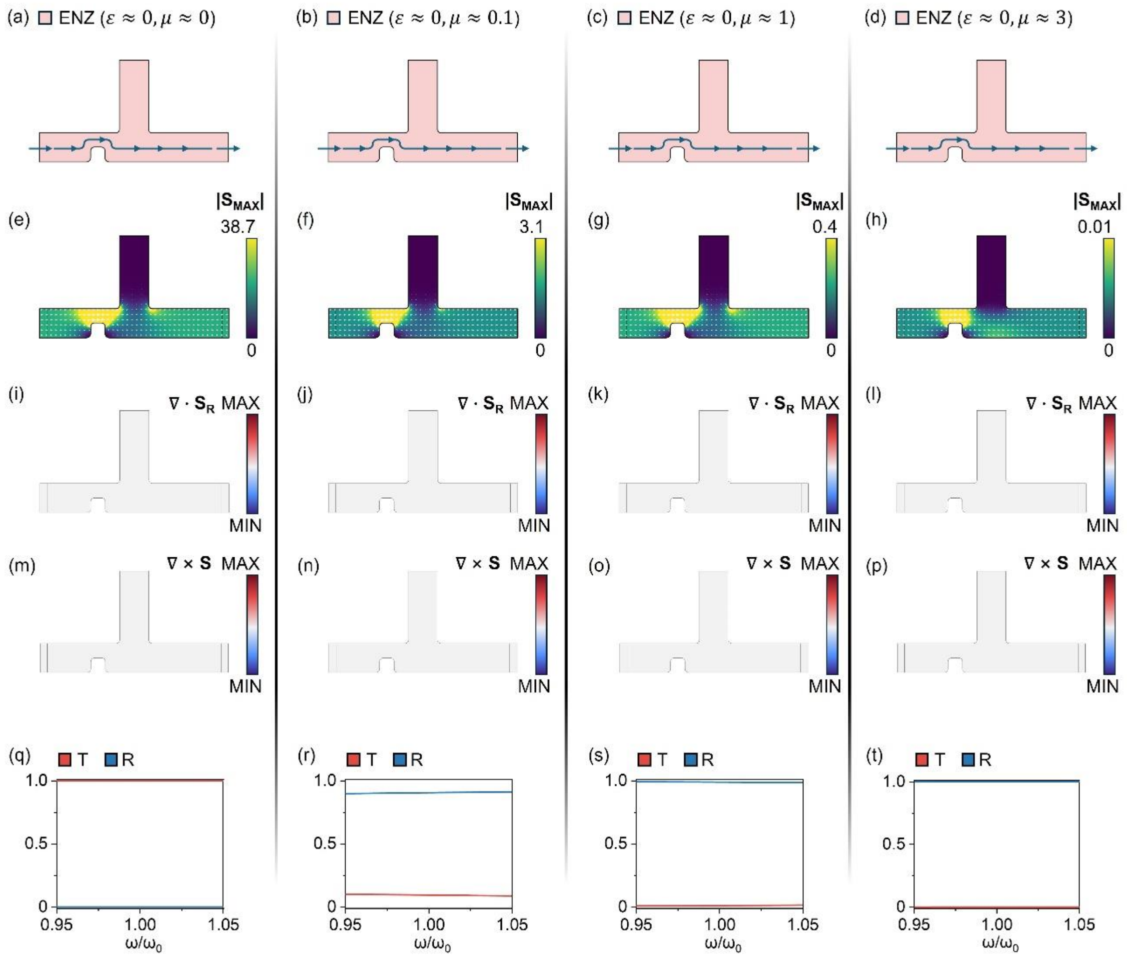

Supplementary Fig. S7 | Power-flow distribution of electromagnetic waves in ENZ media with different permeabilities. a–d Schematic diagrams of the structures with ENZ media of varying magnetic permeability. **e–h** Corresponding power-flow distributions in each medium, where white arrows indicate the direction of power flow. **i–l** Distributions of the real part of the power-flow divergence for each permeability. **m–p** Distributions of the real part of the power-flow curl for each permeability. **q–t** Transmission spectra of electromagnetic waves in ENZ media with different permeabilities.

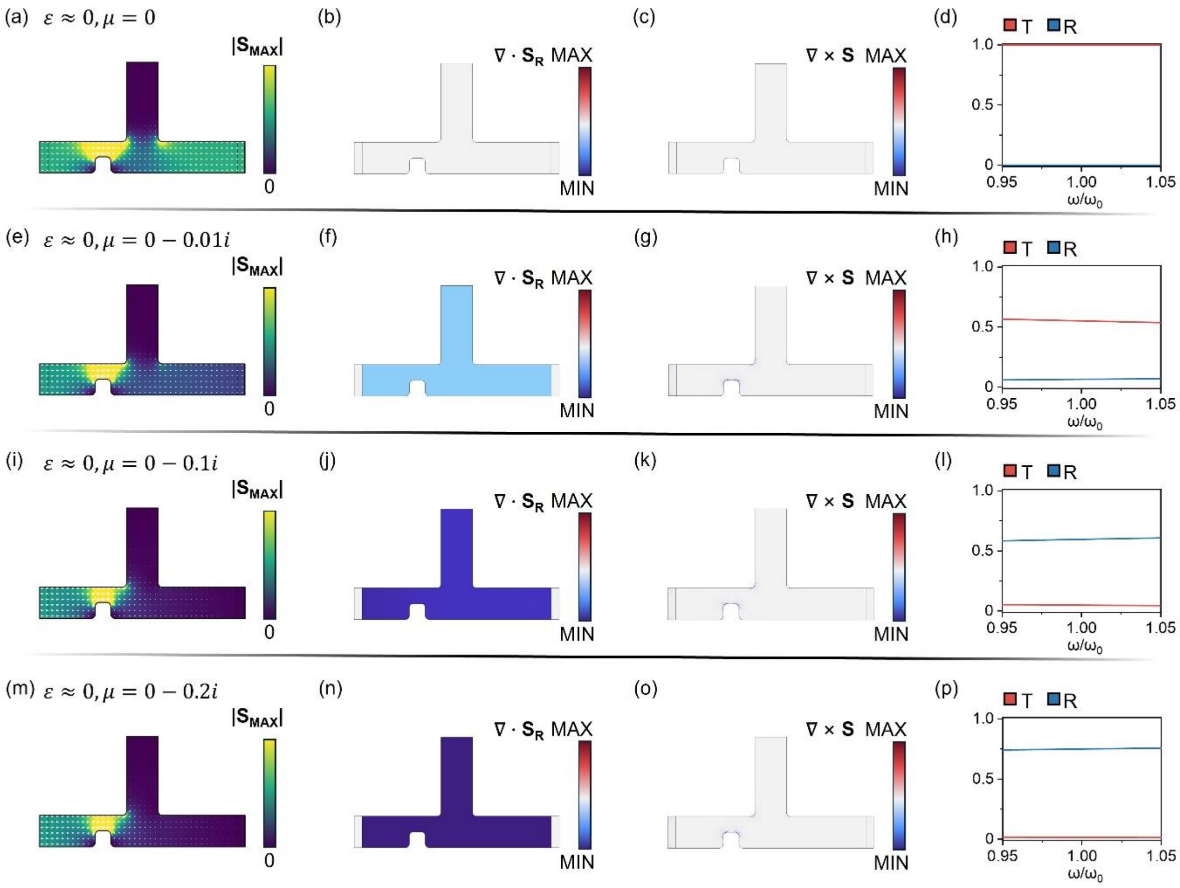

Supplementary Fig. S8 | Schematic diagram of power-flow distribution for electromagnetic waves in the ENZ medium under different permeability losses. a–d Power flow distributions and transmission spectra for $\mu'' = 0$. **e–h** Power flow distributions and transmission spectra for $\mu'' = 0.01$. **i–l** Power flow distributions and transmission spectra for $\mu'' = 0.1$. **m–p** Power flow distributions and transmission spectra for μ''=0.2.

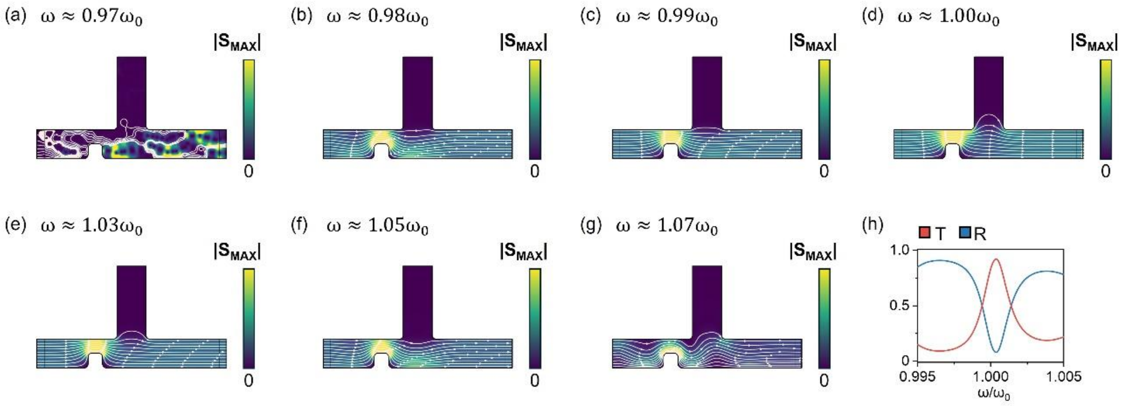

Supplementary Fig. S9 | Frequency response of electromagnetic power-flow distribution in the ENZ medium. Schematic diagrams of the power-flow distribution at different frequencies. **a** $\omega = 0.97\,\omega_0$, **b** $\omega = 0.98\,\omega_0$, **c** $\omega = 0.99\,\omega_0$, **d** $\omega = 1.00\,\omega_0$, **e** $\omega = 1.02\,\omega_0$, **f** $\omega = 1.05\,\omega_0$, **g** $\omega = 1.07\,\omega_0$. **h** corresponding transmission spectrum of the ENZ medium. **i** and **j** Equivalent ε μ spectrum of the medium.

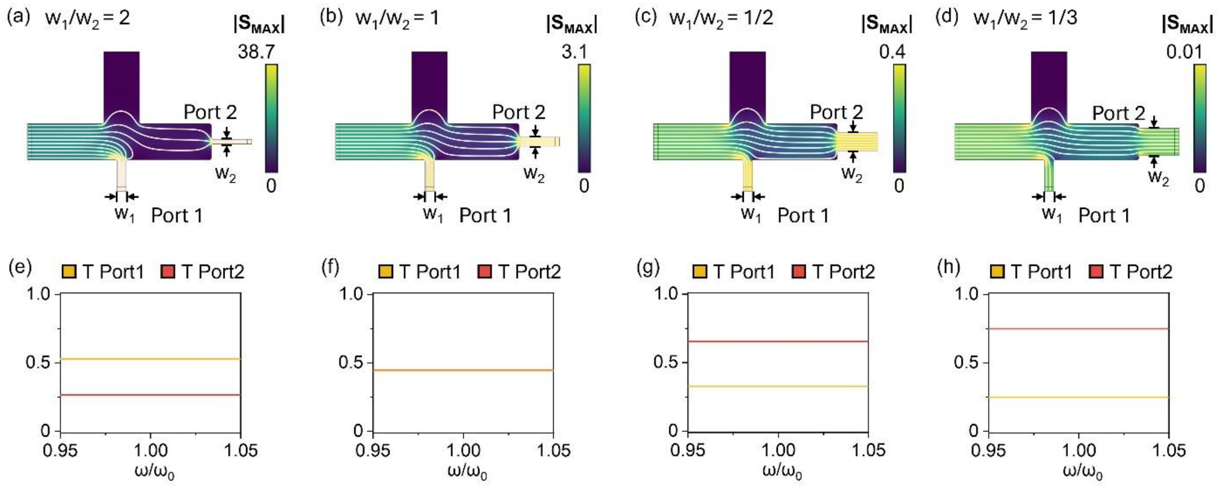

Supplementary Fig. S10 | Power-flow distribution in EMNZ media with multiple output ports. a-b Schematic illustrations of the power-flow distribution for different output-port width ratios. **e-h** Corresponding transmission spectra for the system under the same output-port width ratios.

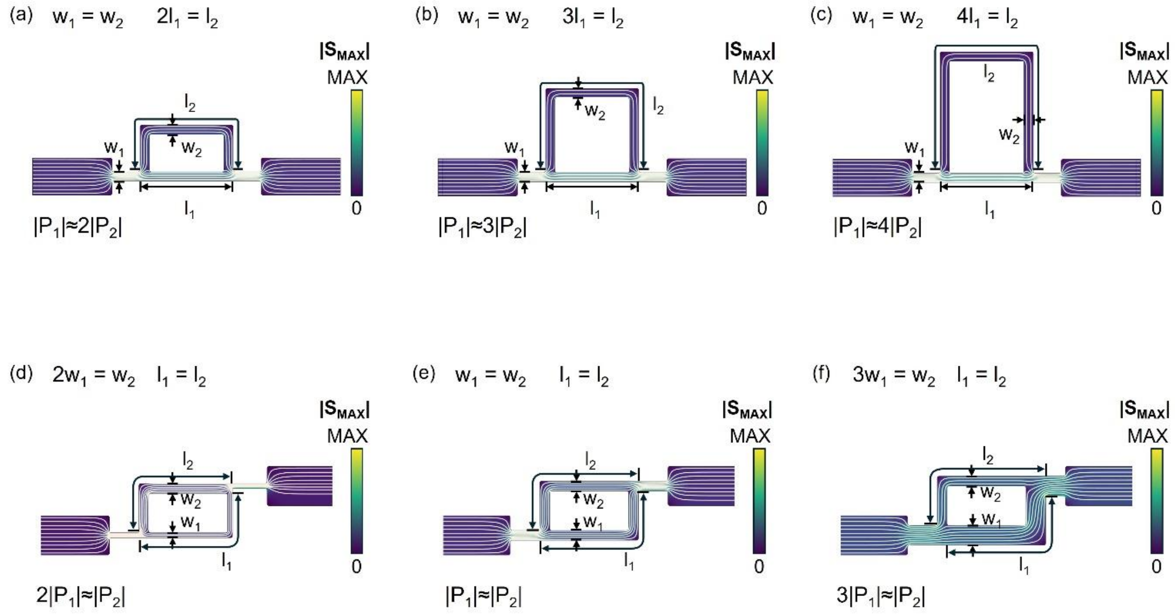

Supplementary Fig. S11 | Power-flow distribution in EMNZ media under multi-path conditions. Two paths, labeled Channel 1 and Channel 2, are considered, with P_1 and P_2 denoting the total power transmitted along each path. a-c Both paths have equal widths but different lengths; the power along each path is inversely proportional to its length, with longer paths carrying less power. **d-f** Both paths have equal lengths but different widths; the power along each path is proportional to its width, with wider paths carrying more power.

.

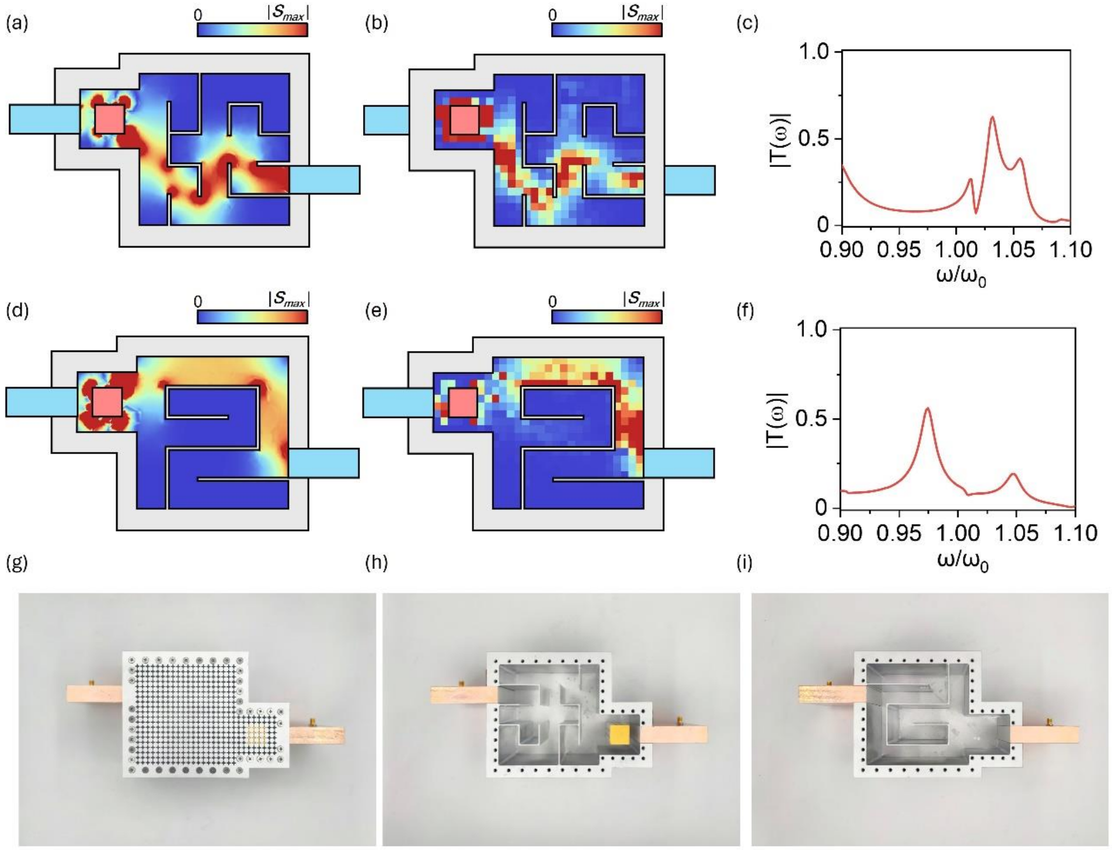

Supplementary Fig. S12 | Schematic diagram and test results of high-precision network. a Simulated power-flow distribution of the high-resolution Case 1. **b** Measured power-flow distribution of the high-resolution Case 1. **c** Measured transmission spectrum of the high-resolution Case 1. **d** Simulated power-flow distribution of the high-resolution Case 2. **e** Measured power-flow distribution of the high-resolution Case 2. **f** Measured transmission spectrum of the high-resolution Case 2. **g** Schematic of the mesh grid used in the newly fabricated high-resolution platform. **h** Internal view of the newly fabricated high-resolution Case 1. **i** Internal view of the newly fabricated high-resolution cases. For detailed measured results, please refer to Supplementary Data 2.

Supplementary Notes

Supplementary Note 1. Pressure-Driven Flow Redistribution in Branched Hydraulic Networks

In this section, we provide a hydraulic analogy to help visualize the physical picture discussed in the main text. Rather than representing any form of "maze solving" or autonomous path selection, the observed water-flow behavior should be understood as a passive and deterministic redistribution process in a branched hydraulic network, governed by boundary conditions, hydraulic head, branch geometry, and air-pressure effects. To illustrate this intuitive analogy through experimental observation, two public online videos are provided for references[1,2].

In video[1], the author constructed a physical model of a labyrinthine tube network using transparent channels. A reservoir at the inlet is filled with water, and, upon releasing the floodgate, the water flows under gravity into the interconnected branches. At the initial stage, the water can enter multiple parallel branches. As the flow develops, the branch with the most favorable hydraulic condition clears first, while transport in other branches is gradually suppressed by trapped air and the associated back pressure. In longer and more tortuous branches, slight elevation changes or bends can trap air through surface-tension effects, forming airlocks that strongly inhibit further water transport. When the hydraulic head is insufficient, even weak surface-tension forces can prevent the flow from advancing. As a result, the observed steady-state flow pattern is established by the combined effects of gravity, branch geometry, hydraulic head, and air-pressure constraints. In this sense, the video provides an intuitive demonstration of pressure-driven flow redistribution in a branched hydraulic network. In particular, it shows that the final flow distribution is strongly shaped by the inlet–outlet boundary conditions and branch-dependent hydraulic resistance, while trapped air and airlocks play a key role in suppressing transport in less favorable branches.

In video[2], a similar phenomenon could also be observed in circuit, which corresponds to the theoretical analysis presented in the Theoretical Analysis section in the main text.

Supplementary Note 2. Ohm's Law of Fluid Flow in Channels.

In this section, we illustrate the Ohm's law of fluid flow in channels and its role in governing liquid flow phenomena within the structure, as referenced in the Theoretical Analysis section in the main text[3]. Firstly, for steady-state, pressure-driven fluid flow in a circular channel, the volumetric flow rate (Q) is described by the Hagen-Poiseuille equation[4]:

$$Q = \frac{\pi R^4}{8L\eta} \Delta P \tag{S1}$$

Here, R is the radius of the channel, ΔP is the pressure difference between the inlet and the outlet, L is the length of the channel, and η is the dynamic viscosity of the fluid. Eq. (S1) is identical to Eq. (7) in the main text, which is referred to as the "Ohm's law of fluid flow in channels". Besides, the average flow velocity, $<v_p>$, can be expressed in terms of the volumetric flow rate as:

$$Q = < v_p > \pi R^2 \tag{S2}$$

Combining Eqs. (S1) and (S2), we get the following the equation

$$< v_p > = \frac{R^2}{8L\eta} \Delta P \tag{S3}$$

For noncircular channels, R can be approximated by the hydrodynamic radius, R_H, which can be obtained as follows:

$$R_H = \frac{wh}{w+h} \tag{S4}$$

where w and h are the width and height of the channel, respectively. From Eq. (S3), it follows that the average flow velocity is directly proportional to the pressure difference (ΔP). This confirms that water flow is pressure-driven, consistent with our theoretical analysis in the main text.

Supplementary Note 3. Additional Applications of the NZI network in Millimeter-Wave and Terahertz Systems

As discussed in the main text, the bus architecture has been demonstrated for interconnection with horn antennas. Here, we present further application examples of the NZI network in millimeter-wave (mmW) and terahertz (THz) systems, highlighting its versatility in device integration. As illustrated in Supplementary Fig. S5a, the network is connected to a frequency-selective filter. An input signal at Port 1 passes through the filter before entering the network, resulting in outputs at Ports 2 and 3 that exhibit strong frequency selectivity, as evidenced by the narrow transmission peak in Supplementary Fig. S5b. Notably, the transmission amplitudes at Ports 2 and 3 are equal, each carrying half the filtered signal power. This configuration effectively functions as a filter-divider module, enabling simultaneous frequency filtering and signal splitting.

A similar topology is shown in Supplementary Fig. S5c, where the filter is replaced by an amplifier. The input signal from Port 1 is amplified before distribution via the network. Simulation results in Supplementary Fig. S5d confirm a significant enhancement in output signal strength compared to the unamplified case, demonstrating the network's capability as an amplifier-divider network.

To further explore the network's applicability in radiating systems, we examine its integration with antenna elements in Supplementary Fig. S5e–f: In panel e, the network is connected to a single antenna, while in panel f, it is connected to an antenna array. These setups are designed to examine the radiation characteristics when powered by an amplified input. The simulated electric field distributions (Supplementary Fig. S5g) reveal distinct radiation patterns for the two configurations. Specifically, the antenna array produces a narrower beamwidth compared to the single antenna, as further validated by the normalized radiation gain patterns in Supplementary Fig. S5h. Additionally, we compare the gain performance with and without amplification. Results indicate an approximate 10 dB gain improvement in both configurations, underscoring the effectiveness of the amplifier- network-antenna integration.

In summary, referring to the results in the main text simultaneously, the proposed mmW/THz configuration enables seamless interconnection and near-lossless transmission for arbitrary devices, enabling the realization of various integrated devices and demonstrating significant potential for inter-device communication.

Supplementary Note 4. Electromagnetic Power Flow in Nonideal EMNZ Media

In our main text, the theoretical assumptions of ideal fluids, namely $\nabla \times \mathbf{S} = 0$ (irrotational) and $\nabla \cdot \mathbf{S} = 0$(incompressible), are idealized. However, in practical applications, particularly on waveguide EMNZ platforms, various non-ideal factors can lead to deviations from these assumptions. Here, we present a quantitative analysis of the robustness of this ideal medium, that is, an analysis of the EMNZ under non-ideal conditions. Specifically, we consider the EM power flow in a medium whose Poynting vector divergence and curl can be described as:

$$\nabla \cdot \mathbf{S} = -\frac{\omega}{2}[(\varepsilon_0\varepsilon''|\mathbf{E}|^2 + \mu_0\mu''|\mathbf{H}|^2) + i(\varepsilon_0\varepsilon'|\mathbf{E}|^2 + \mu_0\mu'|\mathbf{H}|^2)] \tag{S5}$$

$$\nabla \times \mathbf{S} = -\frac{1}{2}[(\mathbf{H}^* \cdot \nabla)\mathbf{E} - (\mathbf{E} \cdot \nabla)\mathbf{H}^* + \mathbf{E}(\nabla \cdot \mathbf{H}^*) - \mathbf{H}^*(\nabla \cdot \mathbf{E})] \tag{S6}$$

Here, the relative permittivity and permeability of the medium are expressed as $\varepsilon = \varepsilon' - i\varepsilon''$and $\mu = \mu' - i\mu''$. From Eq. (S5), for any ideal lossless media ($\varepsilon'', \mu'' \approx 0$), the real part of $\nabla \cdot \mathbf{S}$ is zero, indicating incompressible. This result simply indicates that there is no power sink in a lossless EMNZ medium. For Eq. (S6), when either ε or μ is zero, it can be readily concluded that the curl of the Poynting vector is zero, indicating irrotational. According to the boundary condition, $\mathbf{n} \cdot \mathbf{S}$ is naturally satisfied, indicating inviscid. These three conditions are the characteristics of ideal fluid, which also explains the distribution of EM waves.

For practical applications, however, a truly lossless EMNZ cannot be realized. Considering the EM field conditions in the waveguide-emulated plasmonic ENZ media[5,6], ENZ is emulated by the TE_{10} mode near the cutoff frequency. At this time, we have $\mathbf{H} = \mathbf{z}H_z$and $\mathbf{E} = \mathbf{x}E_x + \mathbf{y}E_y = (-i\omega\varepsilon_0\varepsilon)^{-1}\nabla H_z \times \mathbf{z}$. Accordingly, Eq. (S6) can be rewritten as:

$$\nabla \times \mathbf{S} = \frac{1}{2}(\mathbf{E} \cdot \nabla)\mathbf{H}^* = \frac{1}{2}(\mathbf{x}E_x + \mathbf{y}E_y) \cdot \nabla \cdot \mathbf{z}H_z \tag{S7}$$

For Eq. (S7), the curl of the Poynting vector depends solely on the homogeneity of the magnetic field within the ENZ medium. When $\varepsilon \approx 0$ in the ENZ medium, the magnetic field is uniformly distributed, and therefore $\nabla \times \mathbf{S} = 0$, indicating irrotational. Besides, according to Eq. (S5), for any lossless 2D ENZ medium with $\varepsilon \approx 0$ and $\mu = \mu' \neq 0$, $\nabla \cdot \mathbf{S} = i\mu_0\mu'|\mathbf{H}|^2$, which means $\nabla \cdot \mathbf{S}_{\boldsymbol{R}} = 0$ ($\mathbf{S}_{\boldsymbol{R}}$ represents the real part of Poynting Vector), also indicating incompressibility. Together with $\mathbf{n} \cdot \mathbf{S}_{\boldsymbol{R}} = 0$, the ideal power-flow condition also remains valid in lossless 2D ENZ media. The difference is that, since ε and μ are not equal in this case, the impedance-matching condition is no longer satisfied, leading to reduced transmission. In summary, these results demonstrate that variations in permeability do

not affect the ideal-fluid characteristics of the power flow within the ENZ medium; they only modify the overall system transmittance.

We further validate the above conclusions through simulation. We consider geometry containing dead ends and obstacles. In the EMNZ medium ($\varepsilon \approx 0, \mu \approx 0$), the power flow exhibits ideal fluid characteristics and achieves nearly 100% transmittance, with both $\nabla \times \mathbf{S} = 0$ (irrotational) and $\nabla \cdot \mathbf{S} = 0$ (incompressible) satisfied, as shown in Fig. S6 (b, f, j, n, r). In contrast, the power flow in a conventional air medium is chaotic and disordered, as illustrated in Fig. S6 (a, e, i, m, q). Next, we examine an ideal ENZ medium with a dielectric constant of approximately zero ($\varepsilon \approx 0$), while the permeability is set to $\mu = 1$. According to Eqs. (S5) and (S6), the real parts of both the divergence and the curl of the power flow are predicted to be zero, satisfying the characteristics of an ideal fluid. The power flow indeed exhibits ideal-fluid behavior, as shown in Fig. S6 (c, g, k, o). However, since the permeability μ is not zero, the impedance of this ENZ medium at the ports is mismatched, resulting in nearly zero transmittance, as shown in Fig. S6(s). By adjusting the permeability dopant, the ENZ medium can achieve full transmission while maintaining ideal power flow, as shown in Fig. S6 (d, h, l, p, t). Fig. S7 shows the normalized power-flow distribution in the ENZ medium for different permeability values. As observed, in both ideal ENZ and permeability-doped ENZ media, variations in permeability do not affect the ideal-fluid behavior of the power flow within the medium; they only modify the overall system transmittance.

We also analyze the impact of permeability losses on the EMNZ power flow. According to Eqs. (S5) and (S6), the presence of loss directly induces a non-zero divergence in the power flow, proportional to the imaginary part of the dielectric constant and permeability, which correlates with field attenuation. The effect of loss on the curl of the power flow is more indirect: as the magnetic field becomes non-uniform, analytical prediction becomes difficult. As shown in Fig. S8, non-zero divergence of the power flow increases with μ''. In contrast, the curl of the power flow remains largely robust, as permeability loss does not significantly affect the uniform distribution of the magnetic field in the EMNZ medium. This behavior is also discussed in Ref. 22.

Next, we examine the effect of the permittivity of the medium on the power flow. Eqs. (S5) and (S6) indicate that variations in the permittivity influence the homogeneity of the magnetic field within the medium, and consequently,

the curl of the power flow. To illustrate this, we provide a numerical analysis of the frequency response of the power-flow distribution in the EMNZ medium, as shown in Fig. S9. The permittivity is modeled using a Drude dispersion to match the dispersion characteristics of the waveguide ENZ medium, where the waveguide plasma satisfies $\varepsilon_{\mathrm{eff}} = 1 - (\lambda/2h)^2$. The permeability of the medium is described by a Lorentzian model, $\mu_{\mathrm{eff}} = (f^2 - f_p^2)/(f^2 - f_0^2)$with $f_0 = 0.97 f_p$, to simulate photonic doping in the ENZ medium[3,7]. As shown in Fig. S9, the system exhibits a degree of robustness to frequency variations, maintaining relatively stable ideal-fluid characteristics within a 7% bandwidth. When the frequency offset becomes too large ($\varepsilon \neq 0, \mu \neq 0$), noticeable turbulence and vortices appear.

In general, the permittivity of the medium follows Eqs. (S5) and (S6) and affects the curl and divergence of the power flow. In the system proposed in this manuscript, we construct an ENZ environment using the cutoff frequency of the waveguide. Frequency deviations from the cutoff frequency affect the dielectric constant of the medium, thus influencing the power flow. The permeability of the medium, through doping modulation, only affects the transmittance of the system within the $\varepsilon \approx 0$ frequency band, without affecting the curl and divergence of the power flow. The presence of losses directly causes non-zero divergence in the power flow, proportional to the imaginary parts of the dielectric constant and/or permeability and can be correlated with field attenuation.

References:

1. Steve Mould. Can water solve a maze?. *https://www.youtube.com/watch?v=81ebWToAnvA* (2023).
2. AplhaPhoenix. How does electricity find the "Path of Least Resistance"?. *https://www.youtube.com/watch?v=C3gnNpYK3lo* (2023).
3. Liberal, I., Mahmoud, A. M., Li, Y., Edwards, B. & Engheta, N. Photonic doping of epsilon-near-zero media. *Science* **355**, 1058-1062 (2017).
4. Landau, L. D. & Lifshitz, E. M. *Fluid Mechanics*, Vol. 6 (Pergamon, New York, 1987).
5. Rotman, W. Plasma simulation by artificial dielectrics and parallel-plate media. *IRE Trans. Antennas Propag.* **10**, 82-95 (1962).
6. Li, Y., Liberal, I. & Engheta, N. Structural dispersion-based reduction of loss in epsilon-near-zero and surface plasmon polariton waves. *Sci. Adv.* **5**, eaav3764 (2019).
7. Zhou, Z. *et al.* Substrate-integrated photonic doping for near-zero-index devices. *Nat. Commun.* **10**, 4132 (2019).
