# Supplementary material for: Ohm’s law of electromagnetic ideal fluids: impedance-governed supercoupling in complex near-zero-index networks
Source: Nat Commun. 2026 Jun 2;17:7131. doi: 10.1038/s41467-026-73657-1 (PMC13396760; doi:10.1038/s41467-026-73657-1)
Supplement: Supplementary file 2 — Description Of Additional Supplementary File [file 41467_2026_73657_MOESM2_ESM.pdf]

Description of Additional supplementary files

Supplementary Data 1. Source data used to generate Fig. 3 of the main text.

Supplementary Data 2. Source data used to generate Supplementary Fig. 12.

Supplementary Data 3. Additional simulationgenerated data supporting the conclusions of this study. These data are presented in the main text and Supplementary Information as line plots, scatter plots, and related graphical forms.

Supplementary Movie 1. Visual analogy of the physical phenomenon discussed in the main text, provided to aid visualization and intuitive understanding of the quasi-static power-flow distribution.
