# Supplementary material for: Ohm’s law of electromagnetic ideal fluids: impedance-governed supercoupling in complex near-zero-index networks
Source: Nat Commun. 2026 Jun 2;17:7131. doi: 10.1038/s41467-026-73657-1 (PMC13396760; doi:10.1038/s41467-026-73657-1)
Supplement: Supplementary file 7 — Transparent Peer Review file [file 41467_2026_73657_MOESM7_ESM.pdf]

nature portfolio

Peer Review File

Ohm's Law of Electromagnetic Ideal Fluids: Impedance-Governed Supercoupling in Complex Near-Zero-Index Networks

Corresponding Author: Professor Yue Li

This file contains all reviewer reports in order by version, followed by all author rebuttals in order by version.

Version 0:

Reviewer comments:

Reviewer #1

(Remarks to the Author)
1. Summary of the Manuscript

The authors report an experimental and theoretical study in which electromagnetic waves propagating through near-zero-index (NZI) media appear to "spontaneously escape" maze-like structures by selecting the correct route while avoiding "incorrect branches". They draw a detailed analogy with pressure-driven water navigating mazes and interpret the observed field confinement in terms of an "Ohm's law for EM power flow" in ideal ENZ/EMNZ media.

The manuscript includes full-wave simulations, waveguide-based experimental verification (using a ENZ-like cutoff mode combined with photonic doping), and conceptual extensions toward "extendable supercoupling" and routing architectures.

2. General Assessment

While the experimental implementation is solid and the ENZ waveguide + photonic doping technique is credible, the interpretation of the observed behavior is overstretched, and several claims appear romanticized or framed for dramatic effect ("light solves mazes", "incorrect branches", "spontaneous escape"), which may mislead non-expert readers.

The phenomenon reported—strong field confinement along particular channels in ENZ media—is not new and aligns with known ENZ supercoupling behavior. What the authors present as an emergent, almost intelligent selection mechanism is more rigorously understood as boundary-conditioned quasi-static field distribution in a medium with near-zero effective permittivity (and tuned permeability), resulting in strong impedance-based suppression of dead-end or mismatched paths.

The manuscript therefore requires major revisions, mainly to (i) moderate claims; (ii) clarify the physical mechanisms; and (iii) avoid suggesting novelty where the core principles are already well established.

3. Strengths

Experimental quality
The waveguide-based EMNZ implementation is credible, technically accurate, and well documented. The direct measurement of the Poynting vector is a notable strength.

Clear simulations
The COMSOL/HFSS results convincingly reproduce the expected ENZ confinement and supercoupling behavior across different geometries.

Potential technological relevance
The extension to multi-port supercoupling networks and routing architectures is interesting and might be practically useful.

4. Major Concerns (Require Substantial Revision)

4.1 Overinterpretation of the Physical Mechanism

The central narrative—that light “spontaneously escapes” mazes by rejecting “incorrect branches”—is misleading. In electromagnetic systems, there is no physical notion of a “wrong turn”. The field distribution is fully determined by:

- boundary conditions,
- ENZ quasi-static behavior (phase velocity → ∞),
- impedance mismatches at terminations,
- the lack of allowed modes in dead-end branches.

Thus, what the authors describe as an emergent, pathfinding-like phenomenon is simply ENZ-mediated impedance domination. Calling some branches “incorrect” introduces anthropomorphic language that disconnects the explanation from standard EM theory and may confuse non-specialists.

4.2 Lack of Novelty in the Core Phenomenon

The reported effect appears to be a reformulation of supercoupling and geometry-invariant field transport in ENZ media, extensively explored for nearly twenty years (Silveirinha, Engheta, Edwards, Alù, Liberal, Mahmoud, etc.). The maze geometry does not fundamentally add a new phenomenon; it merely reshapes the ENZ channel network. The field confinement along the lowest-impedance route is well known and expected. The paper currently presents this as a new discovery, whereas the underlying physics is not fundamentally new.

4.3 Theoretical Derivation Relies on Idealized Limits

The derivation of the “Ohm’s law for EM power flow” assumes:

- $\varepsilon \to 0$ exactly,
- $\mu \to 0$ exactly,
- irrotational Poynting vector ($\nabla \times S = 0$),
- incompressibility ($\nabla \cdot S = 0$),
- Neumann boundary conditions on all PEC interfaces,
- perfectly uniform fields along each branch.

These assumptions hold only in the mathematical limit of ideal ENZ and do not rigorously apply to real ENZ/EMNZ media, especially those created via waveguide cutoff, where:

- ENZ behavior occurs only in a narrow band,
- μ_eff tuning depends on the dopant’s subwavelength resonance with ENZ host,
- significant field leakage into side branches is observed (as the authors acknowledge).

Thus the theoretical section, while appealing, is not fully general and should be reframed accordingly.

4.4 Ambiguity in the Dopant’s Role

The description of the photonic dopant could be clearer.
As written, it may suggest that the dopant independently generates $\mu \approx 0$, whereas in reality:

- $\varepsilon \approx 0$ is provided by the waveguide (cutoff TE_10 mode),
- the dopant perturbs the magnetic field in the ENZ host and shifts μ_eff to near zero via the “photonic doping” mechanism,
- the effect depends strongly on local field amplification typical of ENZ environments.

A clearer explanation is needed to avoid misinterpretation, especially by readers unfamiliar with Engheta’s photonic doping literature.

5. Specific Comments and Clarifications Needed

1) Clarify the origin and limitations of ENZ behavior.The physical mechanism behind $\varepsilon \approx 0$ in waveguide cutoff should be made explicit early in the manuscript.
2) Explicitly state that the effect is not “maze solving”.The water analogy is useful for intuition but must be clearly labeled as such, not as a literal physical correspondence.
3) Explain that the “correct path” is the least-impedance route.The current description suggests active selection rather than passive boundary-conditioned distribution.
4) Moderate claims of novelty. The results confirm and nicely visualize known ENZ supercoupling physics, but do not constitute a fundamentally new physical phenomenon.
5) Clarify the frequency sensitivity. EMNZ behavior is realized only at a specific tuned frequency.
The manuscript’s phrasing implies that the effect is broadband, which is not accurate.
6) Avoid strong claims about topology-independence. The results depend on impedance ratios (length/width) and do not guarantee “optimal path selection” in all geometries.

6. Minor Points

- Improve the figure captions to clearly indicate simulated vs. measured fields.
- Avoid introducing new terminology (“spontaneous escape”) without firmly placing it within existing ENZ literature.
- Include quantitative transmission efficiencies for all maze geometries, not only selective examples.
- Consider adding a supplementary note on limitations of the model.

7. Recommendation

Major revision.
The manuscript presents interesting experimental work and a visually appealing demonstration of ENZ supercoupling in complex geometries. However, the current interpretation relies heavily on metaphors and idealized assumptions, and risks misleading readers unfamiliar with ENZ physics. A clearer, more rigorous, and less romanticized description is essential to ensure scientific accuracy and proper contextualization within existing literature.

Reviewer #2

(Remarks to the Author)
The authors report that electromagnetic power flow in a PEC maze filled with a near-zero-index (NZI) / effective epsilon-and-mu-near-zero (EMNZ) medium becomes strongly route-selective: In simulations, the amplitude of the real Poynting vector Re{S} is confined to the (claimed) shortest input-output path with transmission approaching ~100%, whereas a vacuum-filled maze shows broadly distributed/chaotic power flow and very weak delivery to the exit. Experimentally, the authors emulate EMNZ using a parallel-plate waveguide operated at the TE10 cutoff (effective epsilon_r ~ 0) combined with 'photonic doping' (a dielectric inclusion) to tune the effective mu_r toward ~0, and they reconstruct a power-flow map by scanning the magnetic field through a mesh top plate. Two maze layouts show a dominant input-to-output route and suppressed intensity in dead-end branches, broadly consistent with their simulations and their 'Ohm's law for EM power flow' circuit analogy.

I note however that the broader idea that Laplace's equation can 'solve' mazes by producing a harmonic potential whose gradient yields a navigation field is well known in the potential-field / harmonic-function 'path-planning' literature, and it is also widely demonstrated even in popular form (see, e.g.: https://www.youtube.com/shorts/kyYcME2sBws).

Viewed through that lens, the present paper reads as a wave-physics implementation that mimics the static/harmonic case via NZI/EMNZ physics (spatiotemporal decoupling and the 'ideal EM fluid' picture that enables writing S = nabla phi and (under their assumptions) nabla^2 phi = 0 inside the maze). The strongest potentially new contribution is therefore, from my perspective, less the conceptual 'maze solving' itself, and more the maze-network framing of NZI routing plus an experimental visualization/confirmation of route-dominant power flow in a nontrivial maze geometry implemented with a cutoff-based EMNZ platform.

To keep the manuscript's claims maximally precise, I would encourage the authors to tighten what is meant by 'optimal' (shortest geometric length vs. minimum effective 'resistance' a/b vs. something else), quantify robustness (loss, detuning from the EMNZ point, tolerances), and clarify the intended meaning of 'topological path selection' (graph/topology of a network vs. 'topological photonics' with invariants). In particular, the statement that the simulation phenomenon is 'inherently frequency-independent' should be reconciled with the inherently dispersive/narrowband nature of a cutoff-based EMNZ realization and with any residual leakage into dead ends visible in the experimental maps.

In all cases, the authors are asked to carefully address the following questions:

1. The authors' theory uses the 'ideal EM fluid' assumptions (irrotationality and incompressibility) to argue that nabla x S = 0 => S = nabla phi and nabla . S = 0 => nabla^2 phi = 0, in the maze. I want to ask, for the implemented cutoff-based EMNZ platform, what quantitative conditions (loss tangent, distance from cutoff, modal purity, anisotropy introduced by the waveguide emulation, etc.) ensure that nabla x S ~ 0 and nabla . S ~ 0 remain accurate to within (say) a few percent, especially at T-junctions and sharp corners where higher-order evanescent 'content' is expected?

2. What objective is actually being minimized by the selected route? You introduce an effective branch 'resistance' R_S ~ a/b and an Ohm-like relation I_SR_S = Delta_phi with I_S = Sb and Delta_phi = phi_B - phi_A. In mazes with nonuniform widths and multiple competing routes, does the selected route minimize geometric length, minimize sum(a/b), or minimize a more complete effective impedance that includes junction/corner contributions (and possibly loss)? Can you provide a _quantitative_ benchmark comparing predicted route choice to a graph shortest-path computed with weights extracted from full-wave port-to-port admittance/impedance?

3. The experiment reconstructs the relative Re{S} map from measured magnetic fields through a mesh top plate. How sensitive are the inferred direction and magnitude of Re{S} to: (i) probe loading, (ii) finite mesh leakage/perturbation, and (iii) deviations from the assumed TE10 symmetry used to relate fields at z = h/2 to the midplane flow? An uncertainty budget (or calibration against a geometry with analytically predictable S) would strengthen the experimental claim.

4. How does the proposed NZI/EMNZ 'bus' compare to, and inter-operate with, other on-chip wave control and ENZ/NZI toolkits? You propose an extendable supercoupling / 'intelligence bus' for plug-and-play interconnects. Could you quantitatively position this against: (a) guided-wave-driven wavefront-control architectures, such as e.g. in Nature Communications 15, 5992 (2024), and (b) established ENZ/NZI building blocks including ENZ tunneling/supercoupling

theory and experiments and EMNZ/ideal-fluid frameworks (e.g., Phys. Rev. Lett. 97, 157403 (2006); Phys. Rev. Lett. 100, 033903 (2008); Science 355, 1058-1062 (2017); PNAS 117, 24050-24054 (2020))? Specifically, can your maze/bus network serve as a low-loss feed network for guided-wave metasurfaces, and what are the bandwidth and insertion-loss tradeoffs versus a metasurface-native guided-wave distribution network?

5. The authors' simulations state that the effect is 'inherently frequency-independent,' but the EMNZ experimental platform relies on TE10 cutoff and dopant tuning, which are necessarily dispersive. What is the measured operational bandwidth (e.g., 3 dB transmission band and a 'route selectivity' metric band), and how does route selectivity degrade as n departs from near-zero, or as loss increases? A compact scaling law or parametric sweep versus Re(n), Im(n), and maze-junction density would be helpful in translating the concept to mmW/THz/optical implementations.

Reviewer #3

(Remarks to the Author)
Summary:This paper presents a fascinating electromagnetic analogue to the fluid dynamics phenomenon where pressure-driven water spontaneously solves mazes. By utilizing Near-Zero-Index (NZI) media, the authors demonstrate—both theoretically and experimentally—that light can "spontaneously escape" complex maze structures by finding the optimal path and rejecting erroneous turns without external guidance. The work derives an "Ohm’s law for EM power flow," linking potential differences to energy propagation, and proposes a practical application in the form of an "extendable supercoupling" bus for photonic and millimeter-wave circuits.

While the core concept is novel and the potential applications for geometry-agnostic routing are promising, I have several specific questions and concerns regarding the experimental validation, simulation details, and robustness of the proposed mechanism.

Specific Comments and Questions:

1. Mechanism and Multi-path Behavior (Figure 1)
1) Multiple Valid Paths: The current demonstration focuses on scenarios with a single "correct" exit. What happens if there is more than one valid path to an exit? Does the energy split equally, or does it favor the topologically shorter path?
2) Switching Capabilities: Is it possible to tune the system dynamically to select between multiple valid paths, effectively acting as an optical switch? Expanding on this could significantly enhance the practical relevance of the work.

2. Simulation Consistency and Robustness (Figure 2)
1) Intensity Discontinuities: In Figures 2c and 2e, there appear to be discontinuities in the wave path intensity1111. Theoretically, a continuous flow should conserve energy flux across cross-sections (ignoring loss). The visual representation suggests non-uniformity. Could the authors clarify what causes these intensity variations? Are they simulation artifacts or physical effects of the NZI medium?
2) Sensitivity to Index Variations: How critical is the exact refractive index value ($n \approx 0$)? What happens if there is a small deviation or perturbation in the index of the NZI medium? A sensitivity analysis showing how robust the routing is to small changes in n would strengthen the paper.
3) Path Selection in Complex Scenarios: An open question regarding the "intelligence" of the escape mechanism: What happens in a maze with two possible solutions—one "easy" (shorter/direct) and one "harder" (longer/more tortuous)? Does the system prioritize the shortest path, or does energy distribute based on path impedance? Clarifying this behavior is essential for understanding the limits of the proposed "maze-solving" capability.
4) Typographical Errors: There are minor typographical errors in the figure annotations/text (e.g., "topo" notations) that should be corrected for the final manuscript.

3. Experimental Validation and Measurement (Figure 3)
1) Data Resolution: The resolution of the experimental field scans (Figs. 3d, 3g) appears quite low compared to the simulations2222. Is it possible to improve the spatial resolution of the magnetic field probe measurements? Higher-resolution data would more solidly prove the correspondence between the theoretical prediction and physical reality.
2) Dopant Uniformity: The experiment relies on a dielectric ceramic dopant to tune the effective permeability3. How uniform is the effective permeability achieved by this dopant across the waveguide? Non-uniformity could explain discrepancies between simulation and experiment. Can the authors provide a map or estimate of the field uniformity?
3) Probe Perturbation: The experimental results show a non-zero noise floor in regions that should be "dark." This could be induced by the measurement probe itself. Can the authors estimate the magnitude of the perturbation introduced by the magnetic field probe? Quantifying this invasiveness is important for validating the "spontaneous escape" claim.

Conclusion:This paper introduces a high-impact concept with strong theoretical grounding. However, addressing the questions regarding experimental fidelity (resolution, probe perturbation, material uniformity) and the robustness of the physical mechanism (sensitivity to index, multi-path behavior) is necessary to fully validate the claims. I recommend publication subject to these revisions.

Version 1:

Reviewer comments:

Reviewer #1

(Remarks to the Author)
Dear Authors,
I thank you for their detailed response and for the revisions made to the manuscript. The changes have improved clarity in several places, particularly regarding the role of ENZ/EMNZ conditions and the experimental implementation.
However, the central conceptual concern raised in my original report remains only partially addressed. While some terminology has been moderated, the manuscript still conveys an interpretation that may be misleading, especially for readers not deeply familiar with ENZ physics.
The manuscript continues to describe the observed behavior using language that suggests an emergent "pathfinding" or "self-guiding" capability of electromagnetic waves.
Even in its revised form, the narrative still implies that light:
• selects a path,
• avoids certain branches,
• or exhibits an intrinsic routing capability.
This interpretation is not physically accurate.
The observed behavior is more rigorously understood as:
a quasi-static field distribution in an ENZ/EMNZ medium, where the Poynting vector is governed by boundary conditions and impedance contrasts, and where dead-end branches do not support propagating modes.
In this regime, the field configuration is the solution of a boundary-value problem (effectively Laplacian), not the result of any dynamic or selective process.

As such, I strongly recommend that the authors:
1. Explicitly state that the effect is not a form of "maze solving" or path selection, but a consequence of ENZ-induced quasi-static behavior.

2. Clarify the role of impedance in determining the observed routing, making clear that:
o branch geometry (length/width) sets effective impedance,
o the resulting field distribution is passive and deterministic.

3. Avoid residual anthropomorphic or suggestive terminology, even if softened.

4. Clearly state the limitations of the phenomenon, namely that:
o it occurs only under carefully engineered ENZ/EMNZ conditions,
o it is not representative of general electromagnetic propagation.

About Novelty, the revised manuscript still presents the results in a way that may suggest a fundamentally new physical mechanism. I encourage the authors to more clearly position their contribution as:
• an application and visualization of known ENZ supercoupling and quasi-static transport,
rather than:
• a fundamentally new paradigm in wave propagation.

In conclusion, the work is technically sound and experimentally well executed. However, further clarification and reframing of the physical interpretation are necessary to avoid misleading conclusions and to ensure proper contextualization within the ENZ literature.
For these reasons, I still recommend major revision.

Reviewer #2

(Remarks to the Author)
I have read the revised version of the manuscript, and the authors' responses to the three referees.

From my perpspective, the authors have done a good job in carefully addressing all of the referees' questions, and have suitably 'reflected' these amendments in the revised manuscript.

Although there are still some doubts as to the novelty of the presented concept (as indicated by the first referee, too), I am satisfied that the revised work is a solid advance, now (even marginally) meriting publication in NCOMMs, as is.

Reviewer #3

(Remarks to the Author)
I thank the authors for their careful and substantial revisions. They have addressed my previous concerns well, and I find the revised manuscript significantly improved in both clarity and technical support. In particular, the additional explanations, simulations, and experimental clarifications have satisfactorily resolved the main issues I raised.

Overall, I am now supportive of publication.

I only noticed one minor issue: in Fig. R12, the colorbar labeling appears to be reversed. If this figure, or a related version of it, is to be included in the main text or supplementary materials, I suggest that the authors correct the colorbar annotation to

avoid possible confusion.

Other than this small point, I have no further concerns.

Version 2:

Reviewer comments:

Reviewer #1

(Remarks to the Author)
Dear Authors,

As for the actual version of the manuscript, I have noticed all your efforts in following my advices and improving the manuscript that I think now ready for publication without additional modifications.

Kind regards

Open Access This Peer Review File is licensed under a Creative Commons Attribution 4.0 International License, which permits use, sharing, adaptation, distribution and reproduction in any medium or format, as long as you give appropriate credit to the original author(s) and the source, provide a link to the Creative Commons license, and indicate if changes were made.
In cases where reviewers are anonymous, credit should be given to 'Anonymous Referee' and the source.
The images or other third party material in this Peer Review File are included in the article's Creative Commons license, unless indicated otherwise in a credit line to the material. If material is not included in the article's Creative Commons license and your intended use is not permitted by statutory regulation or exceeds the permitted use, you will need to obtain permission directly from the copyright holder.
To view a copy of this license, visit https://creativecommons.org/licenses/by/4.0/

Our Response to the Reviewers' Comments on our Manuscnaript NCOMMS-25-88698 "Spontaneous Escape of Light from Near-Zero-Index Mazes" now entitled "Ohm's Law of Electromagnetic Ideal Fluids: Routing Light in Complex Near-Zero-Index Networks"

Authors: *Wendi Yan, Peihang Li, Jiarui Liu, Kaifeng Li, Pengyu Fu, Shuyu Wang, Mingzhe Hu, and Yue Li*

Response to Reviewer #1:

Reviewer 1 states**: "*****1. Summary of the Manuscript***

The authors report an experimental and theoretical study in which electromagnetic waves propagating through near-zero-index (NZI) media appear to "spontaneously escape" maze-like structures by selecting the correct route while avoiding "incorrect branches". They draw a detailed analogy with pressure-driven water navigating mazes and interpret the observed field confinement in terms of an "Ohm's law for EM power flow" in ideal ENZ/EMNZ media.

The manuscript includes full-wave simulations, waveguide-based experimental verification (using a ENZ-like cutoff mode combined with photonic doping), and conceptual extensions toward "extendable supercoupling" and routing architectures."

Our Response:

We thank the Reviewer for the careful reading of our manuscript and for the concise and accurate summary of our work. We address the specific comments and suggestions in detail below.

Reviewer 1 states**: "*****2. General Assessment***

While the experimental implementation is solid and the ENZ waveguide + photonic doping technique is credible, the interpretation of the observed behavior is overstretched, and several claims appear romanticized or framed for dramatic effect ("light solves mazes", "incorrect branches", "spontaneous escape"), which may mislead non-expert readers.

The phenomenon reported—strong field confinement along particular channels in ENZ media—is not new and aligns with known ENZ supercoupling behavior. What the authors present as an emergent, almost intelligent selection mechanism is more rigorously understood as boundary-conditioned quasi-static field distribution in a medium with near-zero effective permittivity (and tuned permeability), resulting in strong impedance-based suppression of dead-end or mismatched paths.

The manuscript therefore requires major revisions, mainly to (i) moderate claims; (ii) clarify the physical mechanisms; and (iii) avoid suggesting novelty where the core principles are already well established."

Our Response:

We thank the Reviewer for this thoughtful and constructive assessment. We fully acknowledge the concern that parts of the original manuscript employed overly dramatic or anthropomorphic language, which may have led to an overstretched interpretation of the underlying physics.

We agree that the observed route-dominant power transport in the maze structures is fundamentally rooted in well-established ENZ supercoupling and impedance-controlled, boundary-conditioned quasi-static field behavior, rather than in any autonomous or intelligent selection mechanism. In the revised manuscript, we have therefore carefully moderated the narrative to avoid romanticized descriptions (e.g., "light solves mazes" or "incorrect branches") and replaced them with physically precise terminology that emphasizes passive impedance suppression and boundary-conditioned energy flow.

In addition, we have clarified the physical mechanism throughout the Abstract, Introduction, and Discussion, explicitly positioning our results within the existing ENZ/EMNZ literature. The novelty of this work is not the discovery of a new physical effect, but rather an extension of the well-established ENZ supercoupling phenomenon to complex, maze-like geometries. **Specifically, we (1) extend supercoupling from point-to-point configurations to complex networked systems, (2) establish an Ohm's law of ideal electromagnetic fluids as a governing framework for energy redistribution, and (3) experimentally visualize ENZ-mediated power routing.**

We believe these revisions substantially improve the scientific clarity of the manuscript and bring its presentation into closer alignment with established electromagnetic theory and the expectations of the broader readership.

Reviewer 1 states*: "*3. Strengths

Experimental quality
The waveguide-based EMNZ implementation is credible, technically accurate, and well documented. The direct measurement of the Poynting vector is a notable strength.

Clear simulations
The COMSOL/HFSS results convincingly reproduce the expected ENZ confinement and supercoupling behavior across different geometries.

Potential technological relevance
The extension to multi-port supercoupling networks and routing architectures is interesting and might be practically useful."

Our Response:
We thank the Reviewer for the positive assessment of the experimental quality, the clarity of the full-wave simulations, and the potential technological relevance of extending near-zero-index supercoupling to multi-port networks and routing architectures. We appreciate these encouraging comments.

Reviewer 1 states*: "*4. Major Concerns (Require Substantial Revision)
4.1 Overinterpretation of the Physical Mechanism

The central narrative—that light "spontaneously escapes" mazes by rejecting "incorrect branches"—is misleading. In electromagnetic systems, there is no physical notion of a "wrong turn". The field distribution is fully determined by:

- boundary conditions,
- ENZ quasi-static behavior (phase velocity → ∞),
- impedance mismatches at terminations,
- the lack of allowed modes in dead-end branches.

Thus, what the authors describe as an emergent, pathfinding-like phenomenon is simply ENZ-mediated impedance domination. Calling some branches "incorrect" introduces

anthropomorphic language that disconnects the explanation from standard EM theory and may confuse non-specialists."

Our Response: We thank the reviewer for this important comment and fully agree that electromagnetic power transport does not involve any notion of "decision-making" or "wrong turns". In the original manuscript, we used these analogies only as a pedagogical aid to help convey an intuitive understanding of the observed power redistribution. We acknowledge that such analogies may introduce misleading. The field distribution in our system is governed by boundary conditions, the quasi-static nature of ENZ media, and impedance mismatches at branch terminations.

According to the comment, we have carefully revised our manuscript, to avoid any anthropomorphic language and perhaps misleading. We have systematically revised the manuscript to address this concern. Relevant phrases have been rephrased as follows:
"Spontaneously escapes" -> "Routing".
"Incorrect branches"/"Wrong turns" -> "PEC terminated branches"/"Mismatched branches"
"Correct branches"/"Right turns"-> "Impedance-matched outputs"
"Pathfinding" -> "Directional power redistribution"
"Maze solving" -> "Network Routing"
"Solution path" -> "Dominant transmission path"
"Reject branches" -> "Exhibits strongly suppressed power transport"
"Intelligent routing" -> "Impedance-governed routing"

Following these refinements, we have systematically revised the Abstract, Introduction, Results, Figure captions, and Conclusion to ensure that the routing behavior is consistently framed as passive, deterministic, and impedance-dominated, without any implication of active decision-making or intelligence.

Reviewer 1 states*: "*4.2 Lack of Novelty in the Core Phenomenon

The reported effect appears to be a reformulation of supercoupling and geometry-invariant field transport in ENZ media, extensively explored for nearly twenty years (Silveirinha, Engheta, Edwards, Alù, Liberal, Mahmoud, etc.). The maze geometry does not fundamentally add a new phenomenon; it merely reshapes the ENZ channel network. The field confinement along the lowest-impedance route is well known and expected.

The paper currently presents this as a new discovery, whereas the underlying physics is not fundamentally new.”

Our Response: We thank the reviewer for this important comment. We agree that the underlying physics is not fundamentally new, and that the maze geometry serves as a more complex setting for established ENZ transport behavior. In response, we have revised the manuscript to more clearly define the scope of our contribution and to avoid any implication that we are claiming a new underlying mechanism.
In the revised manuscript, we clarify that the contribution of the present work lies primarily in the following three aspects:

(1) Extension of ENZ supercoupling to complex networks:
We extend ENZ supercoupling from conventional isolated point-to-point channel configurations to complex, multi-branch networked systems, and demonstrate how ENZ-mediated transport manifests at the system level under nontrivial boundary conditions.

(2) Establishment of the Ohm’s law of ideal EM fluids:
We derive an Ohm’s law of ideal electromagnetic power flow, in which the Poynting-vector potential difference between ports and the effective resistance $R_S \sim a / b$ jointly determines the power-flow distribution. This provides a unified theoretical framework for describing and predicting energy redistribution in complex NZI structures.

(3) Direct experimental visualization of power-flow routing:
We provide direct experimental visualization of electromagnetic power flow in ENZ networks by reconstructing the Poynting-vector distribution across maze-like structures, enabling direct observation of routing behavior under identical geometries.

To reflect these points and more clearly articulate the nature of our contributions, we have revised the Introduction, Results, and Conclusion to moderate claims of novelty and to clearly position the work as a system-level extension, theoretical derivation, and experimental visualization of established ENZ supercoupling physics, rather than the discovery of a new underlying mechanism.

Based on this comment, we have revised the descriptions in the third paragraph of **Introduction** as followings:

"In this work, we investigate the redistribution and routing of EM power flow in complex NZI structures, and draw an intuitive analogy to pressure-driven flow in fluidic networks. Specifically, power injected into NZI-filled maze-like networks in preferentially redistributed toward impedance-matched output ports, while power flow into strongly mismatched or terminated branches is intrinsically suppressed. Both simulations and experimental results validate this striking behavior. The mechanism is governed by an Ohm's law of ideal EM fluids, in which the Poynting-vector potential difference between ports and the effective impedance width-to-length raito determines the power-flow intensity, providing a predictive framework for guiding EM power redistribution in complex NZI structures. Building upon this mechanism, we extend supercoupling from isolated channels to multi-port NZI networks and discuss a waveguide-based mmWave/THz "bus" as a conceptual, passive EM interconnect for distributing signals or power among multiple components through a shared NZI platform. Our findings not only offer new perspectives on supercoupling and electromagnetic-fluid-like transport in NZI media, and suggest a potential pathway toward geometry-tolerant routing and interconnection in complex photonic and microwave networks, including mmWave/THz integrated architectures."

Reviewer 1 states*: "*4.3 Theoretical Derivation Relies on Idealized Limits

The derivation of the "Ohm's law for EM power flow" assumes:

- $\varepsilon \rightarrow 0$ exactly,
- $\mu \rightarrow 0$ exactly,
- irrotational Poynting vector ($\nabla \times S = 0$),
- incompressibility ($\nabla \cdot S = 0$),
- Neumann boundary conditions on all PEC interfaces,
- perfectly uniform fields along each branch.

These assumptions hold only in the mathematical limit of ideal ENZ and do not rigorously apply to real ENZ/EMNZ media, especially those created via waveguide cutoff, where:

- ENZ behavior occurs only in a narrow band,
- μ_eff tuning depends on the dopant's subwavelength resonance with ENZ host,
- significant field leakage into side branches is observed (as the authors acknowledge).

Thus the theoretical section, while appealing, is not fully general and should be reframed accordingly."

Our Response: We thank the reviewer for this insightful and technically accurate comment. We fully agree that the derivation of the "Ohm's law for electromagnetic power flow" is formulated under idealized conditions corresponding to the mathematical limit of ENZ/EMNZ media, and that these assumptions do not rigorously hold in real, experimentally implemented systems.

Following the reviewer's concerns, we have now explicitly discussed the three key non-ideal factors relevant to practical implementations: (1) the theoretical derivation when the permittivity and/or permeability are not exactly zero, (2) the dopant-induced dispersion associated with tuning the effective permeability. (3) the finite bandwidth of the ENZ response near waveguide cutoff frequency, and (4) residual field leakage into side branches. These effects and their influence on the considered structures are now discussed in Supplementary Note 4, which addressed the impact of non-ideal conditions on the proposed framework.

(1) Theoretical derivation when permittivity and/or permeability are not exactly zero: Specifically, we consider the electromagnetic power flow in a medium, where the divergence and curl of the Poynting vector are described by [R1, R2]:

$$\nabla \cdot \mathbf{S} = -\frac{\omega}{2}[(\varepsilon_0\varepsilon''|\mathbf{E}|^2 + \mu_0\mu''|\mathbf{H}|^2) + i(\varepsilon_0\varepsilon'|\mathbf{E}|^2 + \mu_0\mu'|\mathbf{H}|^2)] \quad \text{(R1)}$$

$$\nabla \times \mathbf{S} = -\frac{1}{2}[(\mathbf{H}^* \cdot \nabla)\mathbf{E} - (\mathbf{E} \cdot \nabla)\mathbf{H}^* + \mathbf{E}(\nabla \cdot \mathbf{H}^*) - \mathbf{H}^*(\nabla \cdot \mathbf{E})] \quad \text{(R2)}$$

The dielectric constant and permeability of the medium are expressed as $\varepsilon = \varepsilon' - i\varepsilon''$ and $\mu = \mu' - i\mu''$, respectively. First, according to Eq. (R1), it follows that in any lossless EMNZ medium ($\varepsilon'', \mu'' \approx 0$), the real part of $\nabla \cdot \mathbf{S}$ is zero, indicating incompressible. From Eq. (R2), when either ε or μ approaches zero, $\nabla \times \mathbf{S}$ is also zero, indicating irrotational. According to boundary condition, $\mathbf{n} \cdot \mathbf{S}$ is naturally satisfied, indicating inviscid. This is why electromagnetic power flow in an ideal EMNZ medium exhibits the same characteristics as an ideal fluid, and it also explains the physical mechanism by which electromagnetic waves can achieve routing. Second, for lossless 2D ENZ medium with $\varepsilon \approx 0$ and $\mu = \mu' \neq 0$, according to Eq. (R1), $\nabla \cdot \mathbf{S} = i\mu_0\mu'|\mathbf{H}|^2$, which means $\nabla \cdot \mathbf{S}_{\boldsymbol{R}} = 0$ ($\mathbf{S}_{\boldsymbol{R}}$ represents the real part of Poynting Vector), also indicating incompressible. Besides, in ENZ medium with TM excited, the magnetic field $\mathbf{H}_z$ must become spatially constant to avoid a divergent electric field, which makes the curl term vanish as well. As a result, $\nabla \times \mathbf{S}_{\boldsymbol{R}} = 0$ is also satisfied, indicating irrotational. Together with $\mathbf{n} \cdot \mathbf{S}_{\boldsymbol{R}} = 0$, the ideal power-flow condition also remains valid in lossless 2D ENZ media. The difference is that, since ε and μ are not equal in this case, the impedance-matching condition is no longer satisfied,

leading to reduced transmission. For TE-polarized waves in MNZ media, an entirely analogous derivation can be obtained by electromagnetic duality. The corresponding conclusions have already been validated in Fig. R1. According to Fig. R1(c, g, k, o, t), for ENZ medium, the power-flow distribution in the system remains the same as that of the ideal EMNZ case in Fig. R1(b, f, j, n, s), whereas the transmission drops to zero. **In summary, these results demonstrate that variations in permeability do not affect the ideal-fluid characteristics of the power flow within the ENZ medium; they only modify the overall system transmittance.**

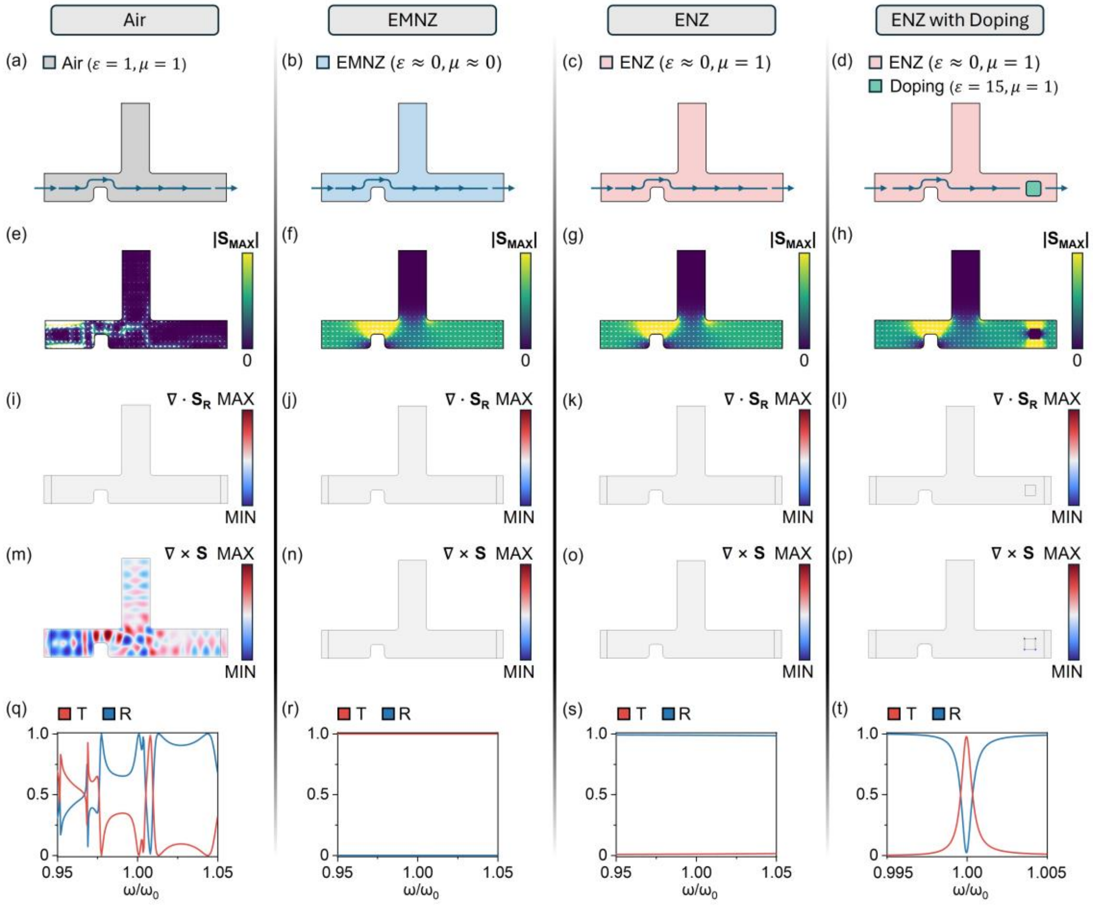

Fig. R1. Power-flow routing of electromagnetic waves in different media. (a–d) Schematic diagrams of the structures with air, EMNZ, ENZ, and ENZ with doping, respectively. (e–h) Corresponding power-flow distributions in each medium, where the white arrows indicate the direction of power flow. (i–l) Distributions of the real part of the power-flow divergence for each medium. (m–p) Distributions of the real part of the power-flow curl for each medium. (q–t) Transmission spectra of electromagnetic waves in the four media.

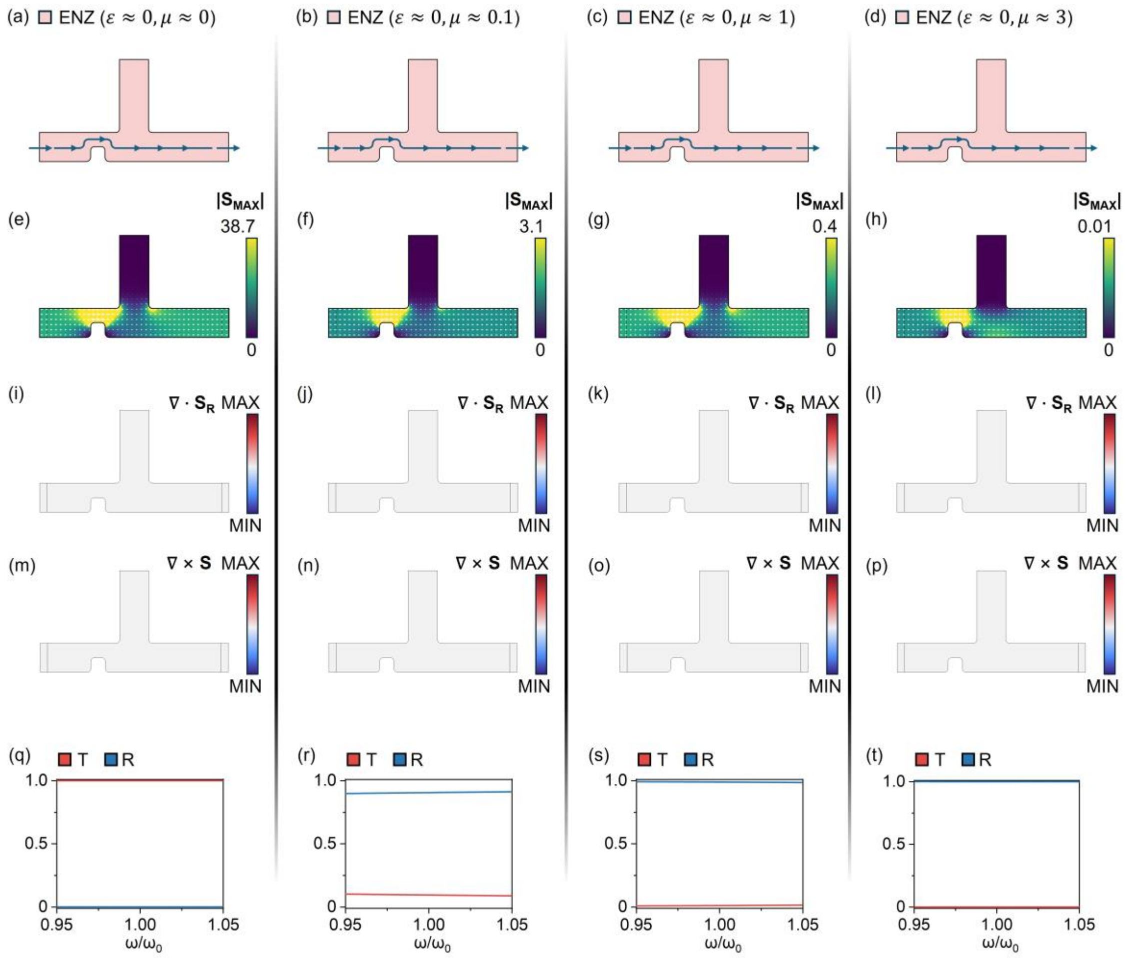

Fig. R2. Power-flow routing of electromagnetic waves in ENZ media with different permeabilities. (a–d) Schematic diagrams of the structures with ENZ media of varying magnetic permeability. (e–h) Corresponding power-flow distributions in each medium, where white arrows indicate the direction of power flow. (i–l) Distributions of the real part of the power-flow divergence for each permeability. (m–p) Distributions of the real part of the power-flow curl for each permeability. (q–t) Transmission spectra of electromagnetic waves in ENZ media with different permeabilities.

(2) The dopant-induced dispersion associated with tuning the effective permeability:

According to what we have said in first part of this response, the **variations in permeability do not affect the ideal-fluid characteristics of the power flow within the ENZ medium; they only modify the overall system transmittance.** According to Ref. R3, introducing a dopant into an ENZ medium can be fully interpreted as an effective tuning of the overall permeability. The purpose of this tuning is to achieve impedance matching and thereby enhance the system transmittance. Apart from the ideal power-flow behavior of circumventing obstacles, which has already been discussed in Refs. R1 and R4, the dopant does not introduce any additional effect on the power-flow distribution. This phenomenon is also verified by the simulations, as shown in Fig. R1 (d, h, l, p, t). According to Fig. R1 (h), no additional power enters the side branch. Moreover, as shown in Fig. R1

(l) and Fig. R1 (p), the curl and divergence of $\mathbf{S}_{\boldsymbol{R}}$ remain zero. In summary, the dopant-induced dispersion mainly affects the impedance-matching condition and thus the overall transmittance, but does not alter the ideal-fluid characteristics or routing behavior of the power flow in the ENZ medium.

(3) The finite bandwidth of the ENZ response near waveguide cutoff frequency:

Next, we investigate the effect of frequency on the power flow. Eqs. (R1) and (R2) indicate that changes in the dielectric constant alter the magnetic field uniformity within the medium, thereby affecting the curl of the power flow. To illustrate this, we perform a numerical analysis of the frequency response of the power flow distribution in the ENZ medium, as shown in Fig. R3. The dielectric constant is modeled using the Drude dispersion model to match the waveguide ENZ medium, where the waveguide-emulated plasmonic effective permittivity satisfies $\varepsilon_{\mathrm{eff}} = 1 - (\lambda/2h)^2$. The medium's permeability is described using a Lorentzian model, $\mu_{\mathrm{eff}} = (f^2 - f_p^2)/(f^2 - f_0^2)$, with $f_0 = 0.97 f_p$, to simulate photonic doping in the ENZ medium (Refs. R1 and R4). As shown in Figure R3, the system exhibits robustness to frequency variations, maintaining nearly ideal-fluid characteristics within a 7% bandwidth. When the frequency deviates further ($\varepsilon \neq 0, \mu \neq 0$), significant turbulence and vortices appear.

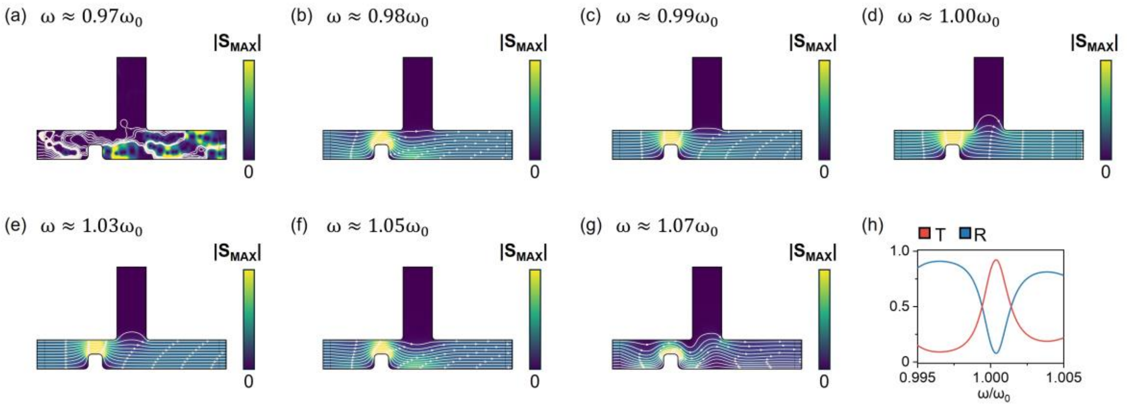

Fig. R3. Frequency response of electromagnetic power-flow distribution in the ENZ medium. Schematic diagrams of the power-flow distribution at different frequencies: (a) $\omega = 0.97\ \omega_0$, (b) $\omega = 0.98\ \omega_0$, (c) $\omega = 0.99\ \omega_0$, (d) $\omega = 1.0\ \omega_0$, (e) $\omega = 1.02\ \omega_0$, (f) $\omega = 1.05\ \omega_0$, (g) $\omega = 1.07\ \omega_0$; (h) corresponding transmission spectrum of the ENZ medium.

(4) Residual field leakage into side branches.

We thank the reviewer for pointing out this issue. The reported field leakage actually arose from a misunderstanding in one of our simulations. In Fig. 5 (b) of the original manuscript, we presented a field distribution with noticeable leakage; this was because the plotted

quantity was the electric field obtained from a simulation with $\varepsilon = 0.01$ and $\mu = 0.01$. In an ideal EMNZ medium, since the magnetic field is uniform, the relative distributions of **E** and **S** are the same. In the actual case, however, because the medium is not perfectly ideal EMNZ and the electrical length is relatively large, the magnetic field is spatially distributed, and therefore **E** can no longer fully represent **S**. In fact, as discussed in Part 3 of this response, our structure is quite robust against frequency variations (i.e., under non-ideal effective $\varepsilon = 0$ conditions), and the leakage is in reality very weak, while the power flow still follows the ideal distribution. In Fig. R4, we have therefore replaced **E** with **S**, from which the leakage is essentially eliminated. We sincerely apologize again for this mistake in the original presentation.

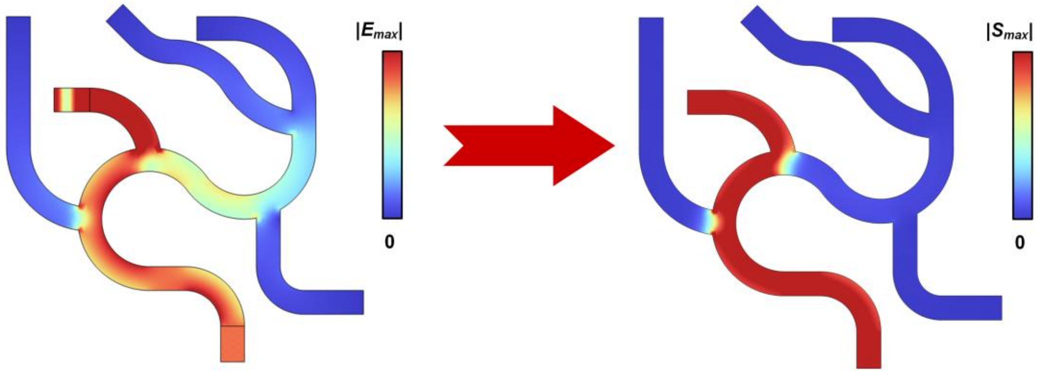

Fig. R4. Updated version of Fig. 5(b) in the main text.

In summary, when either the permittivity or the permeability approaches zero (ENZ or MNZ media), the power flow exhibits ideal-fluid–like behavior, satisfying both $\nabla \times \mathbf{S}_{\boldsymbol{R}} = 0$ and $\nabla \cdot \mathbf{S}_{\boldsymbol{R}} = 0$. In this manuscript, we employ waveguide-based equivalent ENZ media with permeability controlled via doping to emulate an EMNZ medium, and numerical calculations confirm the validity of this equivalence. Specifically, for the waveguide ENZ medium considered here, the irrotational and divergence-free power flow is maintained over a 7% frequency bandwidth, although full transmission occurs only within a comparatively narrow frequency range.

Based on this comment, we have revised our manuscript as follows:
In the **Experimental verifications**:

"As predicted by waveguide-emulated plasmonics, the central cross-section of a waveguide excited in the TE_{10} mode possesses an effective relative permittivity given by $\varepsilon_h = 1 - (\lambda/2h)^2$. Accordingly, a waveguide with height $h = \lambda/2$ can emulate an effectively lossless ENZ medium, yielding an approximate condition of $\varepsilon_r \sim 0$ within a finite bandwidth in our setup. In addition, photonic doping

offers a powerful mechanism for tailoring effective permeability in ENZ media. Benefiting from the intrinsic field-enhancement effect of ENZ environments, embedding a square dielectric inclusion within the ENZ background enables controlled modification of the effective permeability, which can be expressed as:

$$\mu_{\mathrm{eff}} = 1 + \frac{64 l_d^2}{\pi^4 A} \frac{\omega^2}{\omega_{\mathrm{pmc}}{}^2 - \omega^2}, \quad \omega_{\mathrm{pmc}} = \frac{c}{\sqrt{\varepsilon_d}} \sqrt{2(\pi / l_d)^2} \tag{1}$$

"It should be noted that, because both the permittivity and permeability are dispersive, this effect can only be observed in a narrowband structure. In fact, the corresponding bandwidth is about 7%, as shown in Fig. S9"

In the **Theoretical Analysis**:

"In addition, although the above theoretical derivation was carried out for the EMNZ case, the underlying mechanism is in fact highly robust against variations in both permittivity and permeability. It therefore applies to a broad range of NZI implementations, including the waveguide-emulated plasmonic used in our experiments. A detailed discussion can be found in Supplementary Note 4."

In the **Multi-port supercoupling and bus in millimeter and terahertz frequencies:**

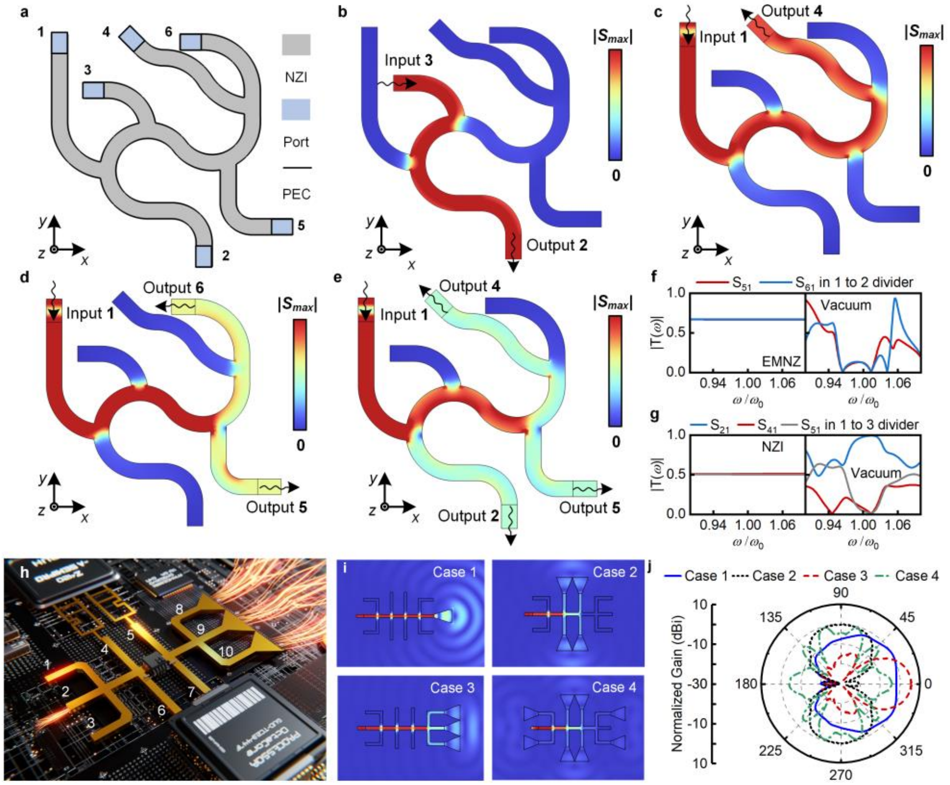

Fig. 5. Multi-port Supercoupling and bus in millimeter and terahertz frequencies.

In **Supplementary Note 4**:

Supplementary Note 4. Electromagnetic Power Flow in Nonideal EMNZ Media

In our main text, the theoretical assumptions of ideal fluids, namely $\nabla \times \mathbf{S} = 0$(irrotational) and $\nabla \cdot \mathbf{S} = 0$(incompressible), are idealized. However, in practical applications, particularly on waveguide EMNZ platforms, various non-ideal factors can lead to deviations from these assumptions. Here, we present a quantitative analysis of the robustness of this ideal medium, that is, an analysis of the EMNZ under non-ideal conditions. Specifically, we consider the EM power flow in a medium whose Poynting vector divergence and curl can be described as:

$$\nabla \cdot \mathbf{S} = -\frac{\omega}{2}\left[(\varepsilon_0\varepsilon''|\mathbf{E}|^2 + \mu_0\mu''|\mathbf{H}|^2) + i(\varepsilon_0\varepsilon'|\mathbf{E}|^2 + \mu_0\mu'|\mathbf{H}|^2)\right] \quad \text{(S5)}$$

$$\nabla \times \mathbf{S} = -\frac{1}{2}\left[(\mathbf{H}^* \cdot \nabla)\mathbf{E} - (\mathbf{E} \cdot \nabla)\mathbf{H}^* + \mathbf{E}(\nabla \cdot \mathbf{H}^*) - \mathbf{H}^*(\nabla \cdot \mathbf{E})\right] \quad \text{(S6)}$$

Here, the relative permittivity and permeability of the media are expressed as $\varepsilon = \varepsilon' - i\varepsilon''$and $\mu = \mu' - i\mu''$. From Eq. (S5), for any ideal lossless media ($\varepsilon'', \mu'' \approx 0$), the real part of $\nabla \cdot \mathbf{S}$ is zero, indicating incompressible. This result simply indicates that there is no power sink in a lossless EMNZ medium. For Eq. (S6), when either ε or μ is zero, it can be readily concluded that the curl of the Poynting vector is zero, indicating irrotational. According to boundary condition, $\mathbf{n} \cdot \mathbf{S}$ is naturally satisfied, indicating inviscid. These three conditions are the characteristics of ideal fluid, which also explains the routing behavior of EM waves in the maze.

For practical applications, however, a truly lossless EMNZ cannot be realized. Considering the EM field conditions in the waveguide-emulated plasmonic ENZ media[35,36], ENZ is emulated by the TE_{10} mode near the cutoff frequency. At this time, we have $\mathbf{H} = \mathbf{z}H_z$ and $\mathbf{E} = \mathbf{x}E_x + \mathbf{y}E_y = (-i\omega\varepsilon_0\varepsilon)^{-1}\nabla H_z \times \mathbf{z}$. Accordingly, Eq (S6) can be rewritten as:

$$\nabla \times \mathbf{S} = \frac{1}{2}(\mathbf{E} \cdot \nabla)\mathbf{H}^* = \frac{1}{2}\left(\mathbf{x}E_x + \mathbf{y}E_y\right) \cdot \nabla \cdot \mathbf{z}H_z \quad \text{(S7)}$$

For Eq. (S7), the curl of the Poynting vector depends solely on the homogeneity of the magnetic field within the ENZ medium. When $\varepsilon \approx 0$ in the ENZ medium, the magnetic field is uniformly distributed, and therefore $\nabla \times \mathbf{S} = 0$, indicating irrotational. Besides, according to Eq. (S5), for any lossless 2D ENZ medium with $\varepsilon \approx 0$ and $\mu = \mu' \neq 0$, $\nabla \cdot \mathbf{S} = i\mu_0\mu'|\mathbf{H}|^2$, which means $\nabla \cdot \mathbf{S}_{\boldsymbol{R}} = 0$ ($\mathbf{S}_{\boldsymbol{R}}$ represents the real part of Poynting Vector), also indicating imcompressible. Together with $\mathbf{n} \cdot \mathbf{S}_{\boldsymbol{R}} = 0$, the ideal power-flow condition also remains valid in lossless 2D ENZ media. The difference is that, since ε and μ are not equal in this case, the impedance-matching condition is no longer satisfied, leading to reduced transmission. In summary, these results demonstrate that variations in permeability do not affect the ideal-fluid characteristics of the power flow within the ENZ medium; they only modify the overall system transmittance.

We further validate the above conclusions through simulation. We consider geometry containing dead ends and obstacles. In the EMNZ medium ($\varepsilon \approx 0, \mu \approx 0$), the power flow exhibits ideal fluid characteristics and achieves nearly 100% transmittance, with both $\nabla \times \mathbf{S} = 0$ (irrotational) and $\nabla \cdot \mathbf{S} = 0$ (incompressible) satisfied, as shown in Fig. S6 (b, f, j, n, r). In contrast, the power flow in a conventional air medium is chaotic and disordered, as illustrated in Fig. S6 (a, e, i, m, q). Next, we examine an ideal ENZ medium with a dielectric constant of approximately zero ($\varepsilon \approx 0$), while the permeability is set to $\mu = 1$. According to Eqs. (S5) and (S6), the real parts of both the divergence and

the curl of the power flow are predicted to be zero, satisfying the characteristics of an ideal fluid. The power flow indeed exhibits ideal-fluid behavior, as shown in Fig. S6 (c, g, k, o). However, since the permeability μ is not zero, the impedance of this ENZ medium at the ports is mismatched, resulting in nearly zero transmittance, as shown in Fig. S6(s). By adjusting the permeability dopant, the ENZ medium can achieve full transmission while maintaining ideal power flow, as shown in Fig. S6 (d, h, l, p, t). Fig. S7 shows the normalized power-flow distribution in the ENZ medium for different permeability values. As observed, in both ideal ENZ and permeability-doped ENZ media, variations in permeability do not affect the ideal-fluid behavior of the power flow within the medium; they only modify the overall system transmittance.

We also analyzed the impact of permeability losses on the EMNZ power flow. According to Eqs. (S5) and (S6), the presence of loss directly induces a non-zero divergence in the power flow, proportional to the imaginary part of the dielectric constant and permeability, which correlates with field attenuation. The effect of loss on the curl of the power flow is more indirect: as the magnetic field becomes non-uniform, analytical prediction becomes difficult. As shown in Fig. S8, non-zero divergence of the power flow increases with μ''. In contrast, the curl of the power flow remains largely robust, as permeability loss does not significantly affect the uniform distribution of the magnetic field in the EMNZ medium. This behavior is also discussed in Ref. 22.

Next, we examine the effect of the permittivity of the medium on the power flow. Eqs. (S4) and (S5) indicate that variations in the permittivity influence the homogeneity of the magnetic field within the medium, and consequently, the curl of the power flow. To illustrate this, we provide a numerical analysis of the frequency response of the power-flow distribution in the EMNZ medium, as shown in Fig. S9. The permittivity is modeled using a Drude dispersion to match the dispersion characteristics of the waveguide ENZ medium, where the waveguide plasma satisfies $\varepsilon_{\text{eff}} = 1 - (\lambda/2h)^2$. The permeability of the medium is described by a Lorentzian model, $\mu_{\text{eff}} = (f^2 - f_p^2)/(f^2 - f_0^2)$with $f_0 = 0.97 f_p$, to simulate photonic doping in the ENZ medium[7, 22]. As shown in Fig. S9, the system exhibits a degree of robustness to frequency variations, maintaining relatively stable ideal-fluid characteristics within a 7% bandwidth. When the frequency offset becomes too large ($\varepsilon \neq 0, \mu \neq 0$), noticeable turbulence and vortices appear.

In general, the permittivity of the medium follows Eqs. (S4) and (S5) and affect the curl and divergence of the power flow. In the system proposed in this manuscript, we construct an ENZ environment using the cutoff frequency of the waveguide. Frequency deviations from the cutoff frequency affect the dielectric constant of the medium, thus influencing the power flow. The permeability of the medium, through doping modulation, only affects the transmittance of the system within the $\varepsilon \approx 0$ frequency band, without affecting the curl and divergence of the power flow. The presence of losses directly causes non-zero divergence in the power flow, proportional to the imaginary parts of the dielectric constant and/or permeability and can be correlated with field attenuation.

[R1] Liberal, I., Lobet, M., Li, Y., & Engheta, N. (2020). Near-zero-index media as electromagnetic ideal fluids. Proceedings of the National Academy of Sciences, 117(39), 24050-24054.Zhou, Z.,
[R2] Li, H., Zhou, Z., Sun, W., Lobet, M., Engheta, N., Liberal, I., & Li, Y. (2022). Direct observation

of ideal electromagnetic fluids. Nature Communications, 13(1), 4747.
[R3] Liberal, I., Mahmoud, A. M., Li, Y., Edwards, B., & Engheta, N. (2017). Photonic doping of epsilon-near-zero media. *Science*, *355*(6329), 1058-1062.
[R4] Zhou, Z., Li, Y., Li, H., Sun, W., Liberal, I., & Engheta, N. (2019). Substrate-integrated photonic doping for near-zero-index devices. Nature communications, 10(1), 4132.
[R5] Liberal, I., Lobet, M., Li, Y., & Engheta, N. (2020). Near-zero-index media as electromagnetic ideal fluids. Proceedings of the National Academy of Sciences, 117(39), 24050-24054.

***Reviewer 1 states*: "4.4 Ambiguity in the Dopant's Role**

The description of the photonic dopant could be clearer.
As written, it may suggest that the dopant independently generates $\mu \approx 0$***, whereas in reality:***
- $\varepsilon \approx 0$ ***is provided by the waveguide (cutoff TE_10 mode),***
- the dopant perturbs the magnetic field in the ENZ host and shifts μ_eff to near zero via the "photonic doping" mechanism,
- the effect depends strongly on local field amplification typical of ENZ environments.
A clearer explanation is needed to avoid misinterpretation, especially by readers unfamiliar with Engheta's photonic doping literature."

Our Response: We sincerely thank the reviewer for this insightful and technically accurate comment. In response, we have revised the manuscript to provide a clearer and more rigorous discussion of the effective permittivity associated with the TE_{10} mode in the waveguide configuration. We have also expanded the discussion on photonic doping in ENZ media, explicitly clarifying that its contribution arises from the field enhancement enabled by the ENZ environment rather than from an intrinsic modification of material parameters.

Based on this comment, we have revised the descriptions in the first paragraph of **Experimental verifications** as followings:

"As predicted by waveguide-emulated plasmonics, the central cross-section of a waveguide excited in the TE_{10} mode possesses an effective relative permittivity given by $\varepsilon_h = 1 - (\lambda/2h)^2$. Accordingly, a waveguide with height $h = \lambda/2$ can emulate an effectively lossless ENZ medium, yielding an approximate condition of $\varepsilon_r \sim 0$ within a finite bandwidth in our setup[35,36]. In addition, photonic doping offers a powerful mechanism for tailoring the effective permeability in ENZ media. Benefiting from the intrinsic field-enhancement effect of ENZ environments, embedding a square dielectric inclusion within the ENZ background enables controlled modification of the effective permeability, which can be expressed as:

$$\mu_{\text{eff}} = 1 + \frac{64 l_d^2}{\pi^4 A} \frac{\omega^2}{\omega_{\text{pmc}}{}^2 - \omega^2}, \ \omega_{\text{pmc}} = \frac{c}{\sqrt{\varepsilon_d}} \sqrt{2(\pi / l_d)^2} \quad (1)$$

where A is the area of the ENZ background, and l_d is the size of the square dopant. The frequency dependance of the effective μ_{eff} is shown in Fig. S9 according to Eq. (1). When the area of the ENZ host is much larger than that of the dopant, the interior of the composite system can be approximately treated as having a uniform effective permeability distribution.. A metal shorting strip, placed parallel to the wide side of the waveguide on the dopant surface, is used to suppress non- TE_{10} modes. Therefore, by precisely designing the size of the dopant, $\mu_r \sim 0$ can be also achieved, thus EMNZ can be simultaneously achieved at the cutoff frequency."

***Reviewer 1 states*: "5. Specific Comments and Clarifications Needed**

1) Clarify the origin and limitations of ENZ behavior. The physical mechanism behind ε ≈ 0 in waveguide cutoff should be made explicit early in the manuscript."

Our Response: We thank the reviewer for this important comment. We agree that clarifying the physical origin and limitations of the $\varepsilon \approx 0$ behavior is essential, especially for readers not deeply familiar with waveguide-emulated near-zero-index media.

In our work, the near-zero permittivity does not arise from a material resonance, but form structural dispersion in a waveguide operated near the cutoff frequency. Specifically, for the dominant TE_{10} mode in a rectangular waveguide filled with vacuum, the propagation constant k can be written as:

$$k = \sqrt{k_0^2 - (\pi/d)^2} = \omega\sqrt{\varepsilon_{\text{eff}}\varepsilon_0} \quad \text{(R3)}$$

Here, k_0 is the free-space wavenumber, d denotes the width of the longer side of the rectangular waveguide, ε_{eff} is the effective permittivity. Within an effective-medium description, the waveguide dispersion relation can be formally mapped to that of a medium with an effective permittivity ε_{eff}. According to Eq. (R1), the relative permittivity is given by

$$\varepsilon_{\text{eff}} = 1 - c^2\pi^2/\omega^2 d^2 \quad \text{(R4)}$$

Where c is the speed of light in free space. Near the condition $d = c\pi / \omega = \lambda/2$, $\varepsilon_{\text{eff}} \sim 0$. Therefore, a waveguide operated near its cutoff frequency, with the TE_{10} mode as the dominant mode, exhibits an effective ENZ response.

Based on this comment, we have revised the descriptions in the first paragraph of **Experimental verifications** as followings:

"As predicted by waveguide-emulated plasmonics, the central cross-section of a waveguide excited in the TE_{10} mode possesses an effective relative permittivity given by $\varepsilon_h = 1 - (\lambda/2h)^2$. Accordingly, a waveguide with height $h = \lambda/2$ can emulate an effectively lossless epsilon-near-zero (ENZ) medium, yielding an approximate condition of $\varepsilon_r \sim 0$ within a finite bandwidth in our setup[35,36]."

Reviewer 1 states*: "*2) Explicitly state that the effect is not "maze solving". The water analogy is useful for intuition but must be clearly labeled as such, not as a literal physical correspondence."

Our Response: We thank the reviewer for this clarification. We fully agree that the reported phenomenon should not be interpreted as "maze solving" in a literal or intelligent sense. In the revised manuscript, we have removed or rephrased all such descriptions and now explain the effect strictly as boundary-conditioned, impedance-dominated electromagnetic power redistribution in near-zero-index media. The pressure-driven water-flow analogy is retained solely as an intuitive aid and is explicitly stated not to represent a literal physical correspondence. Revisions are as follows:
"Spontaneously escapes" -> "Routing".
"Maze solving" -> "Network Routing"

Reviewer 1 states*: "*3) Explain that the "correct path" is the least-impedance route. The current description suggests active selection rather than passive boundary-conditioned distribution."

Our Response: We agree with the reviewer. In the revised manuscript, the term "correct path" has been removed and replaced by a physically precise description: "impedance-matched transport path". We now explicitly clarify that EM power follows impedance-matched routes as a passive consequence of boundary-conditioned redistribution, rather than any form of active path selection.

Based on this comment, we have revised the descriptions as follows:
"correct path" -> "impedance-matched transport path"

Reviewer 1 states*: "*4) Moderate claims of novelty. The results confirm and nicely visualize known ENZ supercoupling physics, but do not constitute a fundamentally new physical phenomenon."

Our Response: We thank the reviewer for this constructive comment and agree that ENZ supercoupling itself is a well-established physical phenomenon. Our intention is not to claim a fundamentally new electromagnetic effect.

The novelty of this work can be summarized in three aspects.

First, we extend ENZ supercoupling from conventional single-channel or single-port configurations to multi-port, multi-branch networks, revealing a system-level, boundary-conditioned routing behavior.

Second, we provide direct experimental visualization of electromagnetic power flow in complex ENZ mazes, which offers intuitive insight into how supercoupling manifests in complex topologies.

Third, we demonstrate the application potential of this mechanism by proposing extendable supercoupling and an NZI-based multi-port bus architecture for plug-and-play energy routing.

Following the reviewer's suggestion, we have moderated the claims of novelty throughout the manuscript, emphasizing that our contribution represents an extension and system-level application of ENZ supercoupling rather than a fundamentally new physical phenomenon. We have revised the descriptions in the third paragraph of **Introduction** as followings

"In this work, we extend NZI supercoupling from isolated channels to complex, multi-branch EM networks, and systematically investigate how EM power distributed and routed boundary-conditioned and impedance-dominated transport. Specifically, we demonstrate that in maze-like structures composed of PECs and filled with NZI media, EM power flow injected at an input port is deterministically redirected toward impedance-matched output ports, while power transport into PEC-terminated branches is intrinsically suppressed[22,23]. This multi-ports routing behavior is directly visualized experimentally using a waveguide-emulated plasmonic platform combined with photonic doping[24-27]. By contrast, in structures filled with vacuum, EM power flow exhibits strong sensitivity to geometry, leading to inefficient energy transport and negligible transmission to the output. Inspired by the analogies to pressure-driven water flow in maze-like networks and to current distribution in direct-current circuits, we derive an Ohm's law of ideal electromagnetic power flow[22,28-30], which provides a simple framework for explaining the observed routing behavior in NZI media. Benefiting from routing in NZI networks, near-total transmission of energy between arbitrary input and output ports can be achieved through any input-output engineering in complex system. This open up new opportunities to implementing a "bus" architecture in optics and microwave circuit[31,32], enabling the integration of plug-

and-play devices for future millimeter-wave (mmW) and terahertz (THz) applications, with significant implications for inter-device communication and routing management."

Reviewer 1 states: "***5) Clarify the frequency sensitivity. EMNZ behavior is realized only at a specific tuned frequency.***"

Our Response: We thank the reviewer for this question. The waveguide exhibits an ENZ response only within a limited frequency range. In the revised manuscript, we have revised the descriptions in the first paragraph of **Experimental verifications** as followings:

"It should be noted that, because both the permittivity and permeability are dispersive, this effect can only be observed in a narrowband structure. In fact, the corresponding bandwidth is about 7%, as shown in Fig. S9"

Reviewer 1 states: "***6) Avoid strong claims about topology-independence. The results depend on impedance ratios (length/width) and do not guarantee "optimal path selection" in all geometries.***"

Our Response: We thank the reviewer for this important comment. In response, we have removed anthropomorphic expressions and clarified any potentially misleading wording in the revised manuscript. In the system under consideration, the electromagnetic field distribution and routing behavior are governed by boundary conditions, the quasi-static characteristics of the ENZ medium, and impedance mismatches at the branch terminations.

Under ideal or near-ideal EMNZ conditions, the final power-flow distribution of the EM wave follows Eq. (6) in the manuscript:

$$SR_S = \Delta\phi \qquad \text{(R5)}$$

Where $R_S = a/b$, and $\Delta\phi = \phi_B - \phi_A$.

Following the revisions, we have systematically amended the Abstract, Introduction, and Results sections to ensure the objectivity of the findings and to eliminate potential misunderstandings or ambiguities.

Reviewer 1 states: "**6. Minor Points**

- Improve the figure captions to clearly indicate simulated vs. measured fields.

- Avoid introducing new terminology ("spontaneous escape") without firmly placing it within existing ENZ literature.

- Include quantitative transmission efficiencies for all maze geometries, not only selective examples.

- Consider adding a supplementary note on limitations of the model."

Our Response: We thank the reviewer for this valuable comment. According to the reviewer's suggestions, we have made the following revisions to the manuscript.

(1) Improve the figure captions: we have improve the figure captions of Fig. 2, Fig. 3, and Fig. 5 to clearly indicate simulated *vs* measured fields.

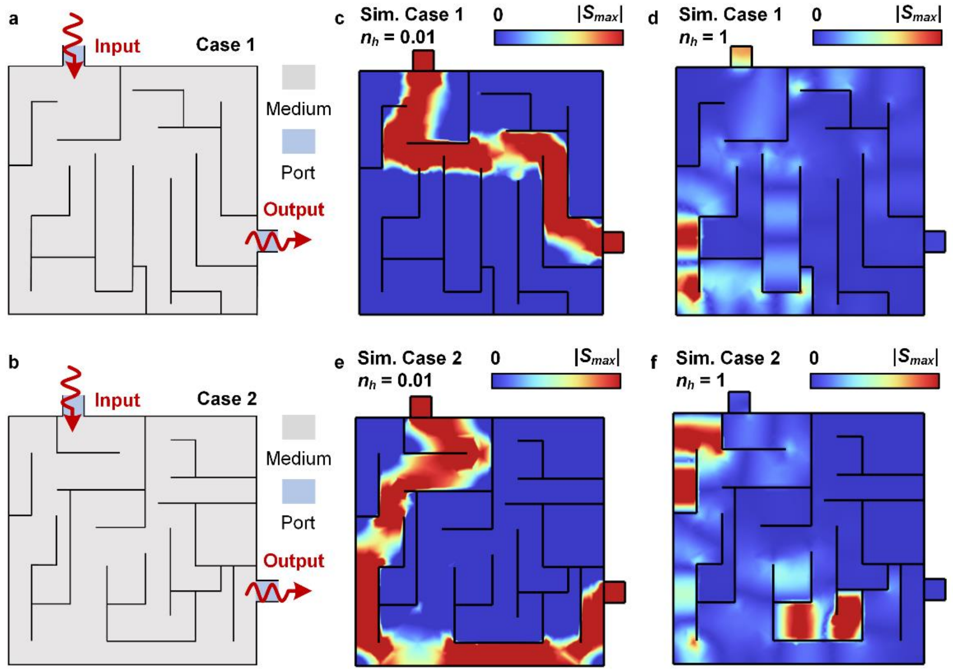

Fig. 2. **Simulated results of boundary-conditioned EM power routing in NZI mazes. a** and (b) Geometries of two randomly generated maze structures, used to demonstrate the generality of the routing behavior. **c** and (e) Simulated amplitude of the real part of Poynting vector field in the maze with different input ports, respectively. The simulated results in vacuum are demonstrated in **d** and (f) for comparison. Sim., Simulated.

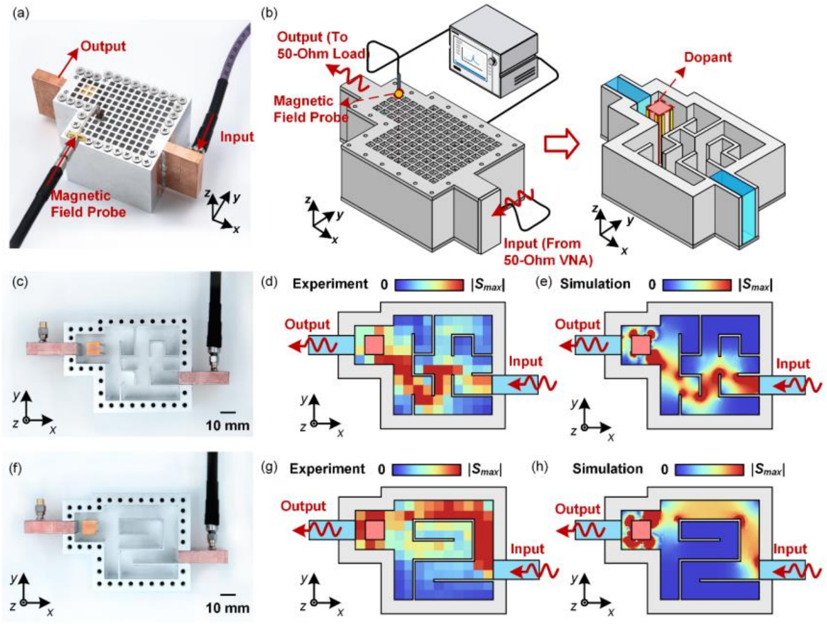

Fig. 3. **Experimental verification. a** A Photograph of the experimental setup. Schematic of the experimental design. **c-e** Top view of the experimental setup (with the cover oriented), along with experimental and simulated power flow results for two different experiments. **d** and (g), experimental results. **e** and (h), simulated results.

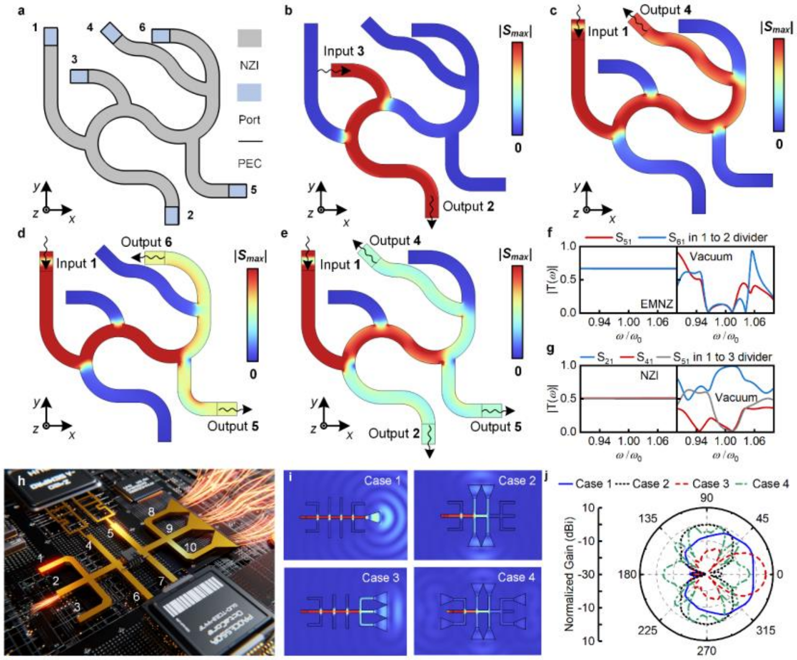

Fig. 5. **Multi-port Supercoupling and bus in millimeter and terahertz frequencies. a** Conceptual illustration of a multi-port supercoupling network. The network is filled with NZI media and is equipped with six ports. **b** and **c**, Simulated amplitude of the real part of Poynting vector field in a multi-port supercoupling network for different input-output configurations, respectively. **d** and (e) Simulated

amplitude of the real part of Poynting vector field with two outputs or three outputs, respectively. These designs can be applied to 1 to 2 or 1 to 3 divider. **f** and (g) Simulated transmission amplitude in (d) and (e). For comparison, transmission amplitudes in vacuum cases are also demonstrated. **h** Conceptual sketch of a multi-port supercoupling bus at millimeter-wave and terahertz frequencies. The bus features ten ports that can interface with different EM components. **i** The simulated electric field distributions of the bus with different integrated horns for 4 representative cases. **j** Corresponding simulated radiation patterns for 4 different cases in (i).

(2) Avoid introducing new terminology ("spontaneous escape") without firmly placing it within existing ENZ literature: We have revised the manuscript to remove this terminology and align the description with established ENZ literature.

(3) Include quantitative transmission efficiencies for all maze geometries, not only selective examples: We have included the transmission efficiencies for all maze geometries; the efficiencies corresponding to Figs. 2 and 3 in the main text are now provided in Figs. S1 and S2:

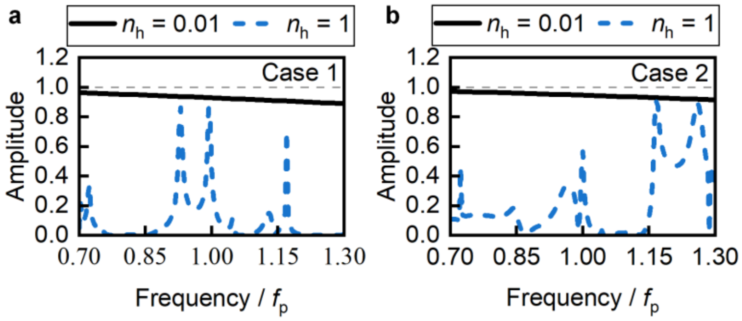

Supplementary Fig. S1 | Simulated transmission amplitude of spontaneous escape of light from NZI mazes, which is demonstrated in Fig. 2 in the main text. a Case 1. **b** Case 2. The central frequency f_p is set as f_p = 3GHz

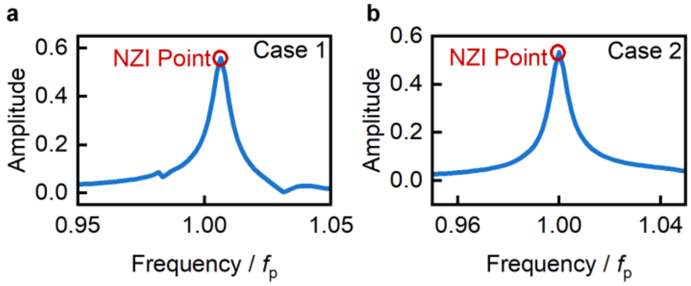

Supplementary Fig. S2 | Measured transmission amplitude of spontaneous escape of light from NZI mazes in Fig. 3 in the main text. a Case 1. **b** Case 2. Both measured frequencies are near the central frequency f_p = 3.04 GHz (see Materials and methods).

(4) Consider adding a supplementary note on limitations of the model: The supplementary note is provide in Supplementary Note 4:

"**Supplementary Note 4. Electromagnetic Power Flow in Nonideal EMNZ Media**

In our main text, the theoretical assumptions of ideal fluids, namely $\nabla \times \mathbf{S} = 0$ (irrotational) and $\nabla \cdot \mathbf{S} = 0$ (incompressible), are idealized. However, in practical applications, particularly on waveguide EMNZ platforms, various non-ideal factors can lead to deviations from these assumptions. Here, we present a quantitative analysis of the robustness of this ideal medium, that is, an analysis of the EMNZ under non-ideal conditions. Specifically, we consider the EM power flow in a medium whose Poynting vector divergence and curl can be described as:

$$\nabla \cdot \mathbf{S} = -\frac{\omega}{2}[(\varepsilon_0\varepsilon''|\mathbf{E}|^2 + \mu_0\mu''|\mathbf{H}|^2) + i(\varepsilon_0\varepsilon'|\mathbf{E}|^2 + \mu_0\mu'|\mathbf{H}|^2)] \quad \text{(S5)}$$

$$\nabla \times \mathbf{S} = -\frac{1}{2}[(\mathbf{H}^* \cdot \nabla)\mathbf{E} - (\mathbf{E} \cdot \nabla)\mathbf{H}^* + \mathbf{E}(\nabla \cdot \mathbf{H}^*) - \mathbf{H}^*(\nabla \cdot \mathbf{E})] \quad \text{(S6)}$$

Here, the relative permittivity and permeability of the media are expressed as $\varepsilon = \varepsilon' - i\varepsilon''$and $\mu = \mu' - i\mu''$. From Eq. (S5), for any ideal lossless media ($\varepsilon'', \mu'' \approx 0$), the real part of $\nabla \cdot \mathbf{S}$ is zero, indicating incompressible. This result simply indicates that there is no power sink in a lossless EMNZ medium. For Eq. (S6), when either ε or μ is zero, it can be readily concluded that the curl of the Poynting vector is zero, indicating irrotational. According to boundary condition, $\mathbf{n} \cdot \mathbf{S}$ is naturally satisfied, indicating inviscid. These three conditions are the characteristics of ideal fluid, which also explains the routing behavior of EM waves in the maze.

For practical applications, however, a truly lossless EMNZ cannot be realized. Considering the EM field conditions in the waveguide-emulated plasmonic ENZ media[35,36], ENZ is emulated by the TE_{10} mode near the cutoff frequency. At this time, we have $\mathbf{H} = \mathbf{z}H_z$ and $\mathbf{E} = \mathbf{x}E_x + \mathbf{y}E_y = (-i\omega\varepsilon_0\varepsilon)^{-1}\nabla H_z \times \mathbf{z}$. Accordingly, Eq (S6) can be rewritten as:

$$\nabla \times \mathbf{S} = \frac{1}{2}(\mathbf{E} \cdot \nabla)\mathbf{H}^* = \frac{1}{2}\left(\mathbf{x}E_x + \mathbf{y}E_y\right) \cdot \nabla \cdot \mathbf{z}H_z \quad \text{(S7)}$$

For Eq. (S7), the curl of the Poynting vector depends solely on the homogeneity of the magnetic field within the ENZ medium.When $\varepsilon \approx 0$ in the ENZ medium, the magnetic field is uniformly distributed, and therefore $\nabla \times \mathbf{S} = 0$, indicating irrotational. Besides, according to Eq. (S5), for any lossless 2D ENZ medium with $\varepsilon \approx 0$ and $\mu = \mu' \neq 0$, $\nabla \cdot \mathbf{S} = i\mu_0\mu'|\mathbf{H}|^2$, which means $\nabla \cdot \mathbf{S}_R = 0$ ($\mathbf{S}_R$ represents the real part of Poynting Vector), also indicating imcompressible. Together with $\mathbf{n} \cdot \mathbf{S}_R = 0$, the ideal power-flow condition also remains valid in lossless 2D ENZ media. The difference is that, since ε and μ are not equal in this case, the impedance-matching condition is no longer satisfied, leading to reduced transmission. In summary, these results demonstrate that variations in permeability do not affect the ideal-fluid characteristics of the power flow within the ENZ medium; they only modify the overall system transmittance.

We further validate the above conclusions through simulation. We consider geometry containing dead ends and obstacles. In the EMNZ medium ($\varepsilon \approx 0, \mu \approx 0$), the power flow exhibits ideal fluid characteristics and achieves nearly 100% transmittance, with both $\nabla \times \mathbf{S} = 0$ (irrotational) and $\nabla \cdot \mathbf{S} = 0$ (incompressible) satisfied, as shown in Fig. S6 (b, f, j, n, r). In contrast, the power flow in a conventional air medium is chaotic and disordered, as illustrated in Fig. S6 (a, e, i, m, q). Next, we

examine an ideal ENZ medium with a dielectric constant of approximately zero ($\varepsilon \approx 0$), while the permeability is set to $\mu = 1$. According to Eqs. (S5) and (S6), the real parts of both the divergence and the curl of the power flow are predicted to be zero, satisfying the characteristics of an ideal fluid. The power flow indeed exhibits ideal-fluid behavior, as shown in Fig. S6 (c, g, k, o). However, since the permeability μ is not zero, the impedance of this ENZ medium at the ports is mismatched, resulting in nearly zero transmittance, as shown in Fig. S6(s). By adjusting the permeability dopant, the ENZ medium can achieve full transmission while maintaining ideal power flow, as shown in Fig. S6 (d, h, l, p, t). Fig. S7 shows the normalized power-flow distribution in the ENZ medium for different permeability values. As observed, in both ideal ENZ and permeability-doped ENZ media, variations in permeability do not affect the ideal-fluid behavior of the power flow within the medium; they only modify the overall system transmittance.

We also analyzed the impact of permeability losses on the EMNZ power flow. According to Eqs. (S5) and (S6), the presence of loss directly induces a non-zero divergence in the power flow, proportional to the imaginary part of the dielectric constant and permeability, which correlates with field attenuation. The effect of loss on the curl of the power flow is more indirect: as the magnetic field becomes non-uniform, analytical prediction becomes difficult. As shown in Fig. S8, non-zero divergence of the power flow increases with μ''. In contrast, the curl of the power flow remains largely robust, as permeability loss does not significantly affect the uniform distribution of the magnetic field in the EMNZ medium. This behavior is also discussed in Ref. 22.

Next, we examine the effect of the permittivity of the medium on the power flow. Eqs. (S4) and (S5) indicate that variations in the permittivity influence the homogeneity of the magnetic field within the medium, and consequently, the curl of the power flow. To illustrate this, we provide a numerical analysis of the frequency response of the power-flow distribution in the EMNZ medium, as shown in Fig. S9. The permittivity is modeled using a Drude dispersion to match the dispersion characteristics of the waveguide ENZ medium, where the waveguide plasma satisfies $\varepsilon_{\text{eff}} = 1 - (\lambda/2h)^2$. The permeability of the medium is described by a Lorentzian model, $\mu_{\text{eff}} = (f^2 - f_p^2)/(f^2 - f_0^2)$with $f_0 = 0.97 f_p$, to simulate photonic doping in the ENZ medium[7, 22]. As shown in Fig. S9, the system exhibits a degree of robustness to frequency variations, maintaining relatively stable ideal-fluid characteristics within a 7% bandwidth. When the frequency offset becomes too large ($\varepsilon \neq 0, \mu \neq 0$), noticeable turbulence and vortices appear.

In general, the permittivity of the medium follows Eqs. (S4) and (S5) and affect the curl and divergence of the power flow. In the system proposed in this manuscript, we construct an ENZ environment using the cutoff frequency of the waveguide. Frequency deviations from the cutoff frequency affect the dielectric constant of the medium, thus influencing the power flow. The permeability of the medium, through doping modulation, only affects the transmittance of the system within the $\varepsilon \approx 0$ frequency band, without affecting the curl and divergence of the power flow. The presence of losses directly causes non-zero divergence in the power flow, proportional to the imaginary parts of the dielectric constant and/or permeability and can be correlated with field attenuation.

***Reviewer 1 states*:** **"Major revision. The manuscript presents interesting experimental work and a visually appealing demonstration of ENZ supercoupling in complex geometries. However, the current interpretation relies heavily on metaphors and idealized assumptions and risks misleading readers unfamiliar with ENZ physics. A clearer, more rigorous, and less romanticized description is essential to ensure scientific accuracy and proper contextualization within existing literature."**

Our Response: We thank the reviewer for this valuable comment. We have carefully revised the manuscript to remove any anthropomorphic language and to eliminate the potential misunderstandings such wording may cause. These revisions have been implemented systematically throughout the manuscript. We have revised the Abstract, Introduction, Results, figure captions, and Conclusion to ensure that the routing behavior is consistently described as passive, deterministic, and governed by impedance, without implying any active decision-making or intelligence.

Overall, the revised manuscript places greater emphasis on scientific rigor and quantitative analysis while retaining intuitive illustrations to facilitate understanding. We believe these changes have substantially improved the clarity and accuracy of the manuscript and have made it more accessible and informative to readers in both the photonics and microwave communities.

Response to Reviewer #2:

Reviewer 2 states*: "The authors report that electromagnetic power flow in a PEC maze filled with a near-zero-index (NZI) / effective epsilon-and-mu-near-zero (EMNZ) medium becomes strongly route-selective: In simulations, the amplitude of the real Poynting vector Re{S} is confined to the (claimed) shortest input-output path with transmission approaching ~100%, whereas a vacuum-filled maze shows broadly distributed/chaotic power flow and very weak delivery to the exit. Experimentally, the authors emulate EMNZ using a parallel-plate waveguide operated at the TE10 cutoff (effective epsilon_r ~ 0) combined with 'photonic doping' (a dielectric inclusion) to tune the effective mu_r toward ~0, and they reconstruct a power-flow map by scanning the magnetic field through a mesh top plate. Two maze layouts show a dominant input-to-output route and suppressed intensity in dead-end branches, broadly consistent with their simulations and their 'Ohm's law for EM power flow' circuit analogy."

Our Response: We thank the Reviewer for the careful reading of our manuscript and for the concise and accurate summary of our work. We address the specific comments and suggestions in detail below.

Reviewer 2 states*:** ***"I note however that the broader idea that Laplace's equation can 'solve' mazes by producing a harmonic potential whose gradient yields a navigation field is well known in the potential-field / harmonic-function 'path-planning' literature, and it is also widely demonstrated even in popular form (see, e.g.: https://www.youtube.com/shorts/kyYcME2sBws).

Viewed through that lens, the present paper reads as a wave-physics implementation that mimics the static/harmonic case via NZI/EMNZ physics (spatiotemporal decoupling and the 'ideal EM fluid' picture that enables writing S = nabla phi and (under their assumptions) nabla^2 phi = 0 inside the maze). The strongest potentially new contribution is therefore, from my perspective, less the conceptual 'maze solving' itself, and more the maze-network framing of NZI routing plus an experimental visualization/confirmation of route-dominant power flow in a nontrivial maze geometry implemented with a cutoff-based EMNZ platform.

To keep the manuscript's claims maximally precise, I would encourage the authors to tighten what is meant by 'optimal' (shortest geometric length vs. minimum effective 'resistance' a/b vs. something else), quantify robustness (loss, detuning from the EMNZ point, tolerances), and clarify the intended meaning of 'topological path selection' (graph/topology of a network vs. 'topological photonics' with invariants). In particular, the statement that the simulation phenomenon is 'inherently frequency-independent' should be reconciled with the inherently dispersive/narrowband nature of a cutoff-based EMNZ realization and with any residual leakage into dead ends visible in the experimental maps."

Our Response: We would like to express our sincere gratitude to the reviewer for their constructive feedback on our manuscript. The reviewer has raised important points, and we are grateful for the opportunity to clarify and strengthen our contributions.

We fully agree with the reviewer's opinion. Indeed, our approach can be interpreted as a wave-physics implementation of this idea, utilizing the unique properties of near-zero-index (NZI) media, particularly their spatiotemporal decoupling and the "ideal EM fluid"

framework. We aim to highlight the distinctive contribution of our work, which **extends the classical analogy into electromagnetic wave dynamics by demonstrating how EM power routing occurs in complex NZI structures through a novel multi-port supercoupling approach**. To clarify, while the analogy with Laplace's equation and harmonic potentials is indeed present, the key novelty of our work lies in the application of NZI media for energy redistribution in complex networks. The implementation of this concept in a nontrivial, experimentally verified maze geometry, supported by a rigorous theoretical framework based on EMNZ physics, represents what we believe to be the primary contribution of our study. We agree that the strength of the paper lies in the experimental visualization of route-dominant power flow in a maze geometry and the exploration of how NZI physics can enable this effect. We have clarified this framing in the revised manuscript and explicitly emphasized the importance of the multi-port network analogy for future applications in photonic systems.

In response to the reviewers' suggestions, we made the following revisions:

1. 'Optimal' and Path Length: We have clarified the definition of "optimal" in the context of the manuscript. Specifically, we distinguish between geometric shortest path lengths and the effective "resistance Rs", which is defined by the impedance matching between the ports. In our setup, power flow is optimized based on the impedance of the output ports and the characteristic properties of NZI media, rather than strictly by the shortest geometric distance.

2. Robustness Quantification: We have expanded the discussion on the robustness of the system. Specifically, we now quantify the impact of loss and detuning from the ideal EMNZ point, addressing the tolerance to fabrication variations and potential deviations from the ideal theoretical model. These revisions have been incorporated into the Supplementary Note 4.

3. Topological Path Selection: We have revised our terminology to distinguish between classical network topology (graph-based pathfinding) and "topological photonics," thereby eliminating potential ambiguity. We now emphasize that the term "topology" specifically refers to the routing of power through impedance-matched paths in NZI media, rather than to topological invariants used in certain areas of photonics.

4. Frequency Dependence and Leaky Paths: We appreciate the reviewer’s concern regarding the statement that the simulation phenomenon is "inherently frequency-

independent." While the routing phenomenon itself is designed to be robust against geometric variation, we acknowledge that the cutoff-based EMNZ platform inherently exhibits frequency dependence, particularly near the cutoff frequency. In the revised manuscript, we have included a more detailed discussion of the dispersion and narrowband behavior associated with the specific EMNZ platform used in the experiments. Furthermore, we investigated the residual leakage to dead-end branches observed in the experimental data. This phenomenon arises from non-ideal dielectric constant and permeability, primarily due to frequency offsets and experimental errors. Additional details are provided in Supplementary Note 4. To address this, we have conducted more refined tests.

For detailed responses to the above comments, please refer to the following point-by-point replies.

***Reviewer 2 states*:** **"1. *The authors' theory uses the 'ideal EM fluid' assumptions (irrotationality and incompressibility) to argue that nabla ∇×S = 0 => S = ∇φ and ∇·S = 0 => ∇²φ = 0, in the maze. I want to ask, for the implemented cutoff-based EMNZ platform, what quantitative conditions (loss tangent, distance from cutoff, modal purity, anisotropy introduced by the waveguide emulation, etc.) ensure that nabla x S ~ 0 and nabla . S ~ 0 remain accurate to within (say) a few percent, especially at T-junctions and sharp corners where higher-order evanescent 'content' is expected?*"**

Our Response: We sincerely thank the reviewers for their insightful comment. We fully acknowledge that the theoretical assumptions, namely $\nabla \times \mathbf{S} = 0$ (irrotationality) and $\nabla \cdot \mathbf{S} = 0$ (incompressibility), are idealized, and that in practical applications, particularly on waveguide EMNZ platforms, numerous non-ideal factors may cause deviations from these assumptions. In response to the reviewers' questions and concerns, we have quantitatively analyzed the robustness of this EMNZ equivalent medium.

First, we begin with a theoretical analysis. Specifically, we consider the electromagnetic power flow in a medium, where the divergence and curl of the Poynting vector are described by [R6]:

$$\nabla \cdot \mathbf{S} = -\frac{\omega}{2}[(\varepsilon_0\varepsilon''|\mathbf{E}|^2 + \mu_0\mu''|\mathbf{H}|^2) + i(\varepsilon_0\varepsilon'|\mathbf{E}|^2 + \mu_0\mu'|\mathbf{H}|^2)] \quad \text{(R1)}$$

$$\nabla \times \mathbf{S} = -\frac{1}{2}[(\mathbf{H}^* \cdot \nabla)\mathbf{E} - (\mathbf{E} \cdot \nabla)\mathbf{H}^* + \mathbf{E}(\nabla \cdot \mathbf{H}^*) - \mathbf{H}^*(\nabla \cdot \mathbf{E})] \quad \text{(R2)}$$

The dielectric constant and permeability of the medium are expressed as $\varepsilon = \varepsilon' - i\varepsilon''$ and $\mu = \mu' - i\mu''$, respectively. First, according to Eq. (R1), it follows that in any lossless

EMNZ medium ($\varepsilon'', \mu'' \approx 0$), the real part of $\nabla \cdot \mathbf{S}$ is zero, indicating incompressible. From Eq. (R2), when either ε or μ approaches zero, $\nabla \times \mathbf{S}$ is also zero, indicating irrotational. According to boundary condition, $\mathbf{n} \cdot \mathbf{S}$ is naturally satisfied, indicating inviscid. This is why electromagnetic power flow in an ideal EMNZ medium exhibits the same characteristics as an ideal fluid, and it also explains the physical mechanism by which electromagnetic waves can achieve routing. Second, for lossless 2D ENZ medium with $\varepsilon \approx 0$ and $\mu = \mu' \neq 0$, according to Eq. (R1), $\nabla \cdot \mathbf{S} = i\mu_0\mu'|\mathbf{H}|^2$, which means $\nabla \cdot \mathbf{S}_{\boldsymbol{R}} = 0$ ($\mathbf{S}_{\boldsymbol{R}}$ represents the real part of Poynting Vector), also indicating incompressible. Besides, in ENZ medium with TM excited, the magnetic field $\mathbf{H}_{\mathrm{z}}$ must become spatially constant to avoid a divergent electric field, which makes the curl term vanish as well. As a result, $\nabla \times \mathbf{S}_{\boldsymbol{R}} = 0$ is also satisfied, indicating irrotational. Together with $\mathbf{n} \cdot \mathbf{S}_{\boldsymbol{R}} = 0$, the ideal power-flow condition also remains valid in lossless 2D ENZ media. The difference is that, since ε and μ are not equal in this case, the impedance-matching condition is no longer satisfied, leading to reduced transmission. For TE-polarized waves in MNZ media, an entirely analogous derivation can be obtained by electromagnetic duality. **This means that when only one of the parameters (either ε or μ) deviates from zero, the ideal-fluid assumption described in the main text still holds, and the routing behavior in the maze remains essentially unchanged.**

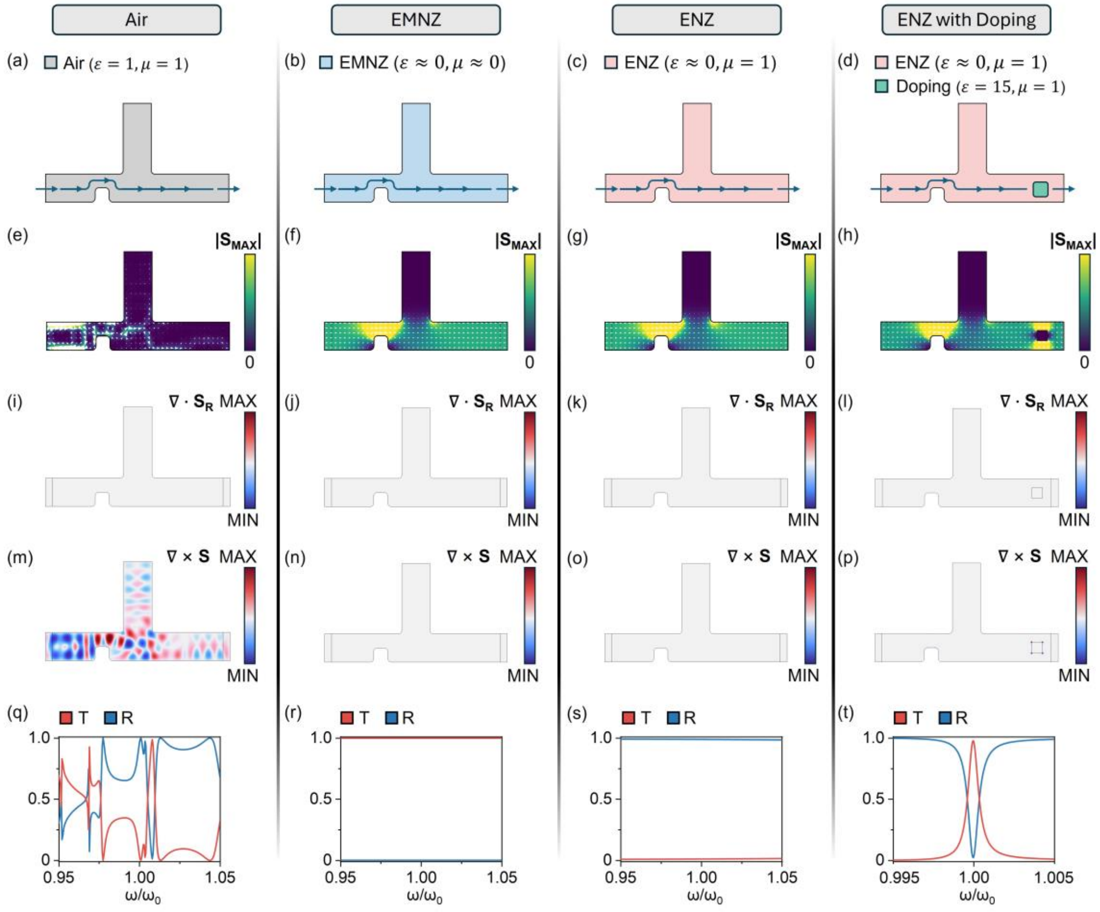

Fig. R5. Power-flow routing of electromagnetic waves in different media. (a–d) Schematic diagrams of the structures with air, EMNZ, ENZ, and ENZ with doping, respectively. (e–h) Corresponding power-flow distributions in each medium, where the white arrows indicate the direction of power flow. (i–l) Distributions of the real part of the power-flow divergence for each medium. (m–p) Distributions of the real part of the power-flow curl for each medium. (q–t) Transmission spectra of electromagnetic waves in the four media.

We further validate the above conclusions through simulation. We consider geometry containing dead ends and obstacles. In the EMNZ medium ($\varepsilon \approx 0, \mu \approx 0$), the power flow exhibits ideal fluid characteristics and achieves nearly 100% transmittance, with both $\nabla \times \mathbf{S} = 0$ (irrotational) and $\nabla \cdot \mathbf{S} = 0$ (incompressible) satisfied, as shown in Fig. R5 (b, f, j, n, r). In contrast, the power flow in a conventional air medium is chaotic and disordered, as illustrated in Fig. R5 (a, e, i, m, q). Next, we examine an ideal ENZ medium with a permittivity of approximately zero ($\varepsilon \approx 0$), while the permeability is set to $\mu = 1$. According to Eqs. (R4) and (R5), the real parts of both the divergence and the curl of the power flow are predicted to be zero, satisfying the characteristics of an ideal fluid. The power flow indeed exhibits ideal-fluid behavior, as shown in Fig. R5 (c, g, k, o). However, since the permeability μ is not zero, the impedance of this ENZ medium at the ports is mismatched, resulting in nearly zero transmittance, as shown in Fig. R5 (s). By adjusting

the permeability dopant, the ENZ medium can achieve full transmission while maintaining ideal power flow, as shown in Fig. R5 (d, h, l, p, t). Fig. R6 shows the normalized power-flow distribution in the ENZ medium for different permeability values. **As observed, in both ideal ENZ and permeability-doped ENZ media, variations in permeability do not affect the ideal-fluid behavior of the power flow within the medium; they only modify the overall system transmittance.** Based on these simulations, we demonstrate that under the ENZ condition, $\nabla \times \mathbf{S}_{\boldsymbol{R}} = 0$ and $\nabla \cdot \mathbf{S}_{\boldsymbol{R}} = 0$ remain valid regardless of the value of the permeability.

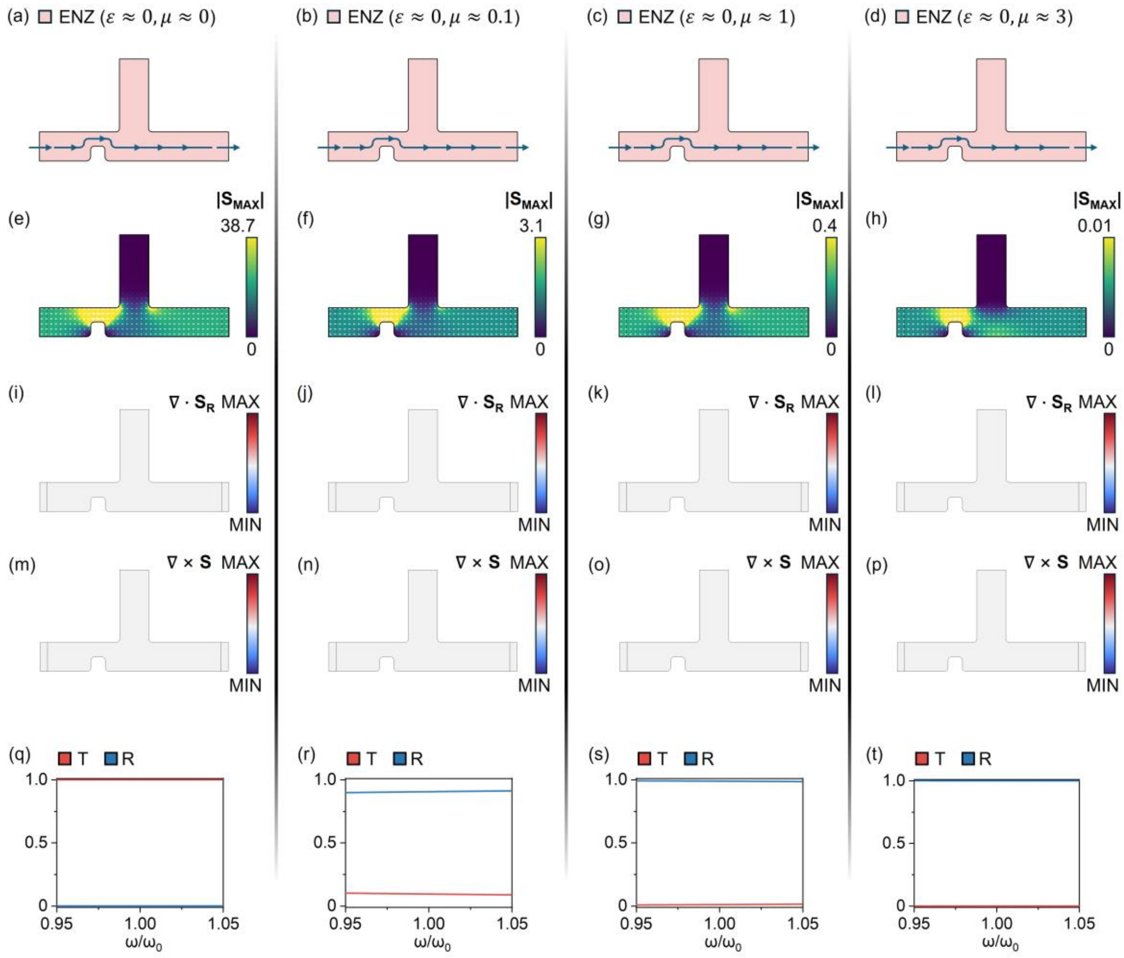

Fig. R6. Power-flow routing of electromagnetic waves in ENZ media with different permeabilities. (a–d) Schematic diagrams of the structures with ENZ media of varying magnetic permeability. (e–h) Corresponding power-flow distributions in each medium, where white arrows indicate the direction of power flow. (i–l) Distributions of the real part of the power-flow divergence for each permeability. (m–p) Distributions of the real part of the power-flow curl for each permeability. (q–t) Transmission spectra of electromagnetic waves in ENZ media with different permeabilities.

Second, we analyze the impact of **permeability losses** on the EMNZ power flow. According to Eqs. (R6) and (R7), the presence of loss directly induces a non-zero

divergence in the power flow, proportional to the imaginary part of the permittivity and permeability, which correlates with field attenuation. The effect of loss on the curl of the power flow is more indirect: as the magnetic field becomes non-uniform, analytical prediction becomes difficult. As shown in Fig. R7, non-zero divergence of the power flow increases with μ''. In contrast, the curl of the power flow remains largely robust, as permeability loss does not significantly affect the uniform distribution of the magnetic field in the EMNZ medium. This behavior is also discussed in Ref. 22. From the streamline distribution, **it can be observed that even when the loss reaches 0.2*i*, the ideal EM power-flow behavior of avoiding mismatched ports is still preserved, indicating that the conclusions exhibit strong robustness.**

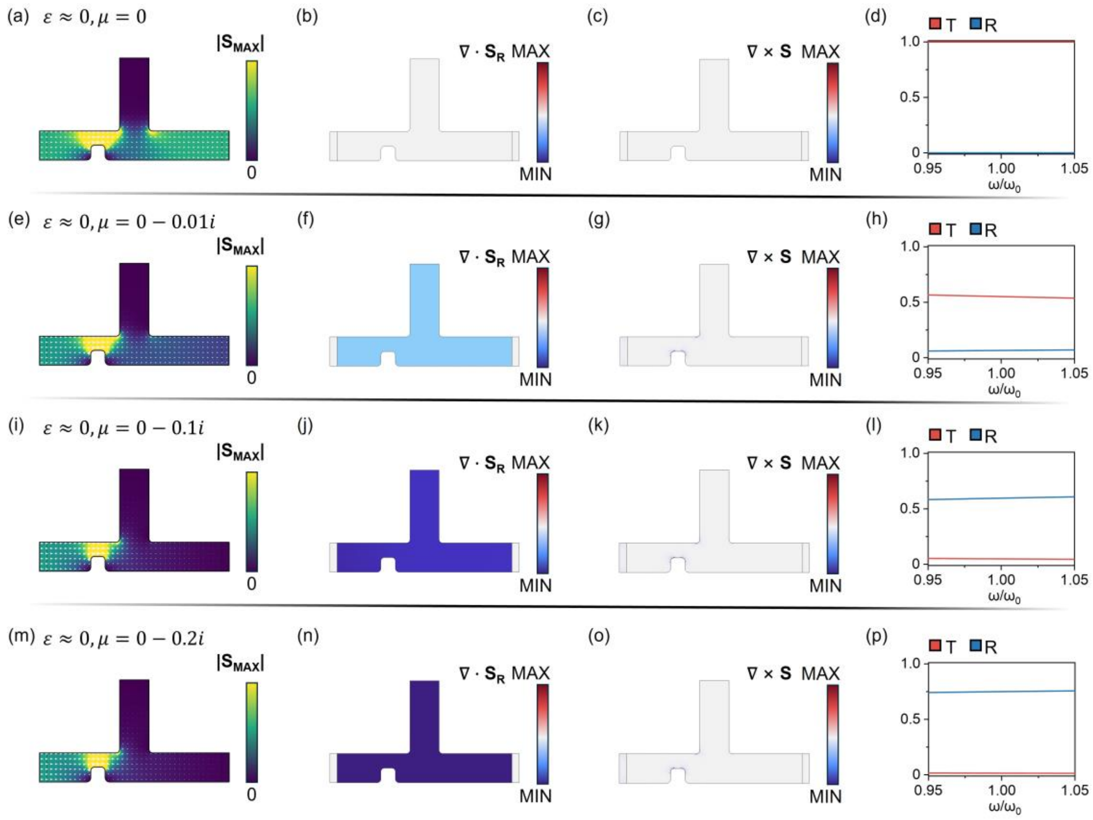

Fig. R7. Schematic diagram of power-flow routes for electromagnetic waves in the ENZ medium under different permeability losses. (a)–(d) Power flow distributions and transmission spectra for $\mu'' = 0$. (e)–(h) Power flow distributions and transmission spectra for $\mu'' = 0.01$. (i)–(l) Power flow distributions and transmission spectra for $\mu'' = 0.1$. (m)–(p) Power flow distributions and transmission spectra for $\mu'' = 0.2$.

Third, we examine the effect of the permittivity of the medium on the power flow. Eqs. (R6) and (R7) indicate that variations in the permittivity influence the homogeneity of the magnetic field within the medium, and consequently, the curl of the power flow. To illustrate this, we provide a numerical analysis of the frequency response of the power-flow

distribution in the EMNZ medium, as shown in Fig. R8. The permittivity is modeled using a Drude dispersion to match the dispersion characteristics of the waveguide ENZ medium, where the waveguide plasma satisfies $\varepsilon_{\text{eff}} = 1 - (\lambda/2h)^2$. The permeability of the medium is described by a Lorentzian model, $\mu_{\text{eff}} = (f^2 - f_p^2)/(f^2 - f_0^2)$ with $f_0 = 0.97 f_p$, to simulate photonic doping in the ENZ medium (Refs. R6 and R7). As shown in Fig. R8, the system exhibits a degree of robustness to frequency variations, **maintaining relatively stable ideal-fluid characteristics within a 7% bandwidth**. When the frequency offset becomes too large ($\varepsilon \neq 0, \mu \neq 0$), noticeable turbulence and vortices appear.

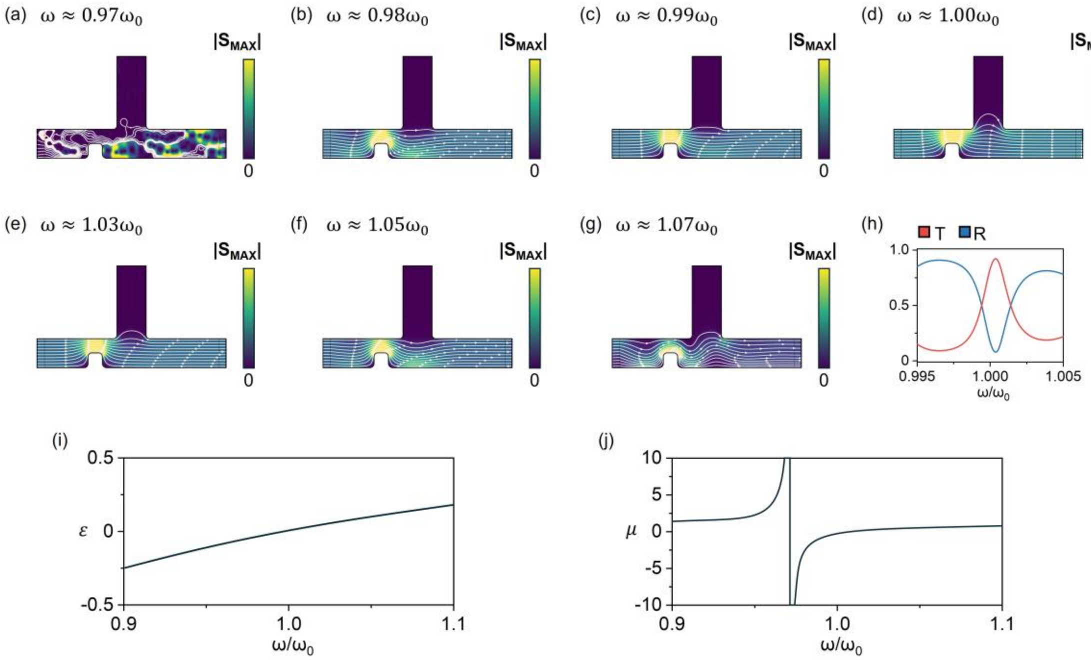

Fig. R8. Frequency response of electromagnetic power-flow distribution in the ENZ medium. Schematic diagrams of the power-flow distribution at different frequencies: (a) $\omega = 0.97\ \omega_0$, (b) $\omega = 0.98\ \omega_0$, (c) $\omega = 0.99\ \omega_0$, (d) $\omega = 1.0\ \omega_0$, (e) $\omega = 1.02\ \omega_0$, (f) $\omega = 1.05\ \omega_0$, (g) $\omega = 1.07\ \omega_0$; (h) corresponding transmission spectrum of the ENZ medium. (i) and (j) Equivalent ε μ spectrum of the medium.

Finally, the issues of modal purity and the anisotropy introduced by the waveguide emulation, as raised by the reviewer, are effectively mitigated by the shorting wires shown in Fig. R9. In our structure, the metallic waveguide boundaries inherently serve the role of these shorting wires, thereby suppressing evanescent waves and maintaining mode purity at corners and junctions.

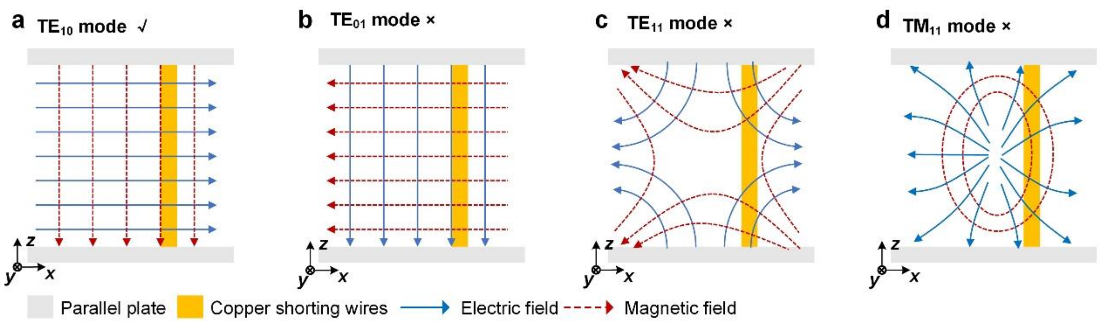

Fig. R9 Electromagnetic mode distribution inside the parallel-plate waveguide. (a) TE_{10} mode. (b) TE_{01} mode. (c) TE_{11} mode. (d) TM_{11} mode. Only modes with electric fields perpendicular to the copper pillars can exist; therefore, this parallel-plate waveguide supports only the TE_{10} mode.

Therefore, we can summarize the quantitative conditions required to satisfy the "ideal EM fluid" assumption raised by the reviewer as follows:

(1) Theoretically, in a lossless medium where either ε or μ alone approaches zero, the ideal-fluid assumption can still hold.

(2) For the loss tangent introduced by the dielectric dopant, simulation results indicate that when the imaginary part of μ satisfies $\mathrm{Im}(\mu) < 0.2$, the ideal-fluid condition remains valid; this requirement can be readily achieved using the zirconia ceramic dopant employed in our experiments.

(3) For the distance from cutoff, simulation results show that the ideal-fluid condition can be maintained within a frequency deviation of 7%.

(4) For the modal purity and anisotropy, these have been minimized as much as possible through the design of shorting wires in the experimental setup.

Based on this comment, we have revised the descriptions in **Experimental verification, Theoretical Analysis, Methods** and **Supplementary Note 4** as followings:

"A metal shorting strip, placed parallel to the wide side of the waveguide on the dopant surface, is used to suppress non-TE_{10} modes."

"It should be noted that, because both the permittivity and permeability are dispersive, this effect can only be observed in a narrowband structure. In fact, the corresponding bandwidth is about 7%, as shown in Fig. S9."

"In other words, within the ideal EMNZ approximation ($\varepsilon_h = 0$, $\mu_h = 0$), the Poynting vector satisfies the conditions of a harmonic field inside NZI media. For the case in non-ideal conditions, please refer to Supplementary Note 4."

"A dielectric rod made of dielectric ceramics with relative permittivity $\varepsilon_d = 37.8 - i0.0037$ has sizes of 11.5 mm × 11.5 mm, locating in the maze to act as a dopant. This level of loss ensures that the ideal electromagnetic fluid behavior can be maintained in the experiment."

"**Supplementary Note 4. Electromagnetic Power Flow in Nonideal EMNZ Media**

In our main text, the theoretical assumptions of ideal fluids, namely $\nabla \times \mathbf{S} = 0$(irrotational) and $\nabla \cdot \mathbf{S} = 0$(incompressible), are idealized. However, in practical applications, particularly on waveguide EMNZ platforms, various non-ideal factors can lead to deviations from these assumptions. Here, we present a quantitative analysis of the robustness of this ideal medium, that is, an analysis of the EMNZ under non-ideal conditions. Specifically, we consider the EM power flow in a medium whose Poynting vector divergence and curl can be described as:

$$\nabla \cdot \mathbf{S} = -\frac{\omega}{2}[(\varepsilon_0\varepsilon''|\mathbf{E}|^2 + \mu_0\mu''|\mathbf{H}|^2) + i(\varepsilon_0\varepsilon'|\mathbf{E}|^2 + \mu_0\mu'|\mathbf{H}|^2)] \tag{S5}$$

$$\nabla \times \mathbf{S} = -\frac{1}{2}[(\mathbf{H}^* \cdot \nabla)\mathbf{E} - (\mathbf{E} \cdot \nabla)\mathbf{H}^* + \mathbf{E}(\nabla \cdot \mathbf{H}^*) - \mathbf{H}^*(\nabla \cdot \mathbf{E})] \tag{S6}$$

Here, the relative permittivity and permeability of the media are expressed as $\varepsilon = \varepsilon' - i\varepsilon''$and $\mu = \mu' - i\mu''$. From Eq. (S5), for any ideal lossless media ($\varepsilon'', \mu'' \approx 0$), the real part of $\nabla \cdot \mathbf{S}$ is zero, indicating incompressible. This result simply indicates that there is no power sink in a lossless EMNZ medium. For Eq. (S6), when either ε or μ is zero, it can be readily concluded that the curl of the Poynting vector is zero, indicating irrotational. According to boundary condition, $\mathbf{n} \cdot \mathbf{S}$ is naturally satisfied, indicating inviscid. These three conditions are the characteristics of ideal fluid, which also explains the routing behavior of EM waves in the maze.

For practical applications, however, a truly lossless EMNZ cannot be realized. Considering the EM field conditions in the waveguide-emulated plasmonic ENZ media[35,36], ENZ is emulated by the TE_{10} mode near the cutoff frequency. At this time, we have $\mathbf{H} = \mathbf{z}H_z$ and $\mathbf{E} = \mathbf{x}E_x + \mathbf{y}E_y = (-i\omega\varepsilon_0\varepsilon)^{-1}\nabla H_z \times \mathbf{z}$. Accordingly, Eq (S6) can be rewritten as:

$$\nabla \times \mathbf{S} = \frac{1}{2}(\mathbf{E} \cdot \nabla)\mathbf{H}^* = \frac{1}{2}(\mathbf{x}E_x + \mathbf{y}E_y) \cdot \nabla \cdot \mathbf{z}H_z \tag{S7}$$

For Eq. (S7), the curl of the Poynting vector depends solely on the homogeneity of the magnetic field within the ENZ medium.When $\varepsilon \approx 0$ in the ENZ medium, the magnetic field is uniformly distributed, and therefore $\nabla \times \mathbf{S} = 0$, indicating irrotational. Besides, according to Eq. (S5), for any lossless 2D ENZ medium with $\varepsilon \approx 0$ and $\mu = \mu' \neq 0$, $\nabla \cdot \mathbf{S} = i\mu_0\mu'|\mathbf{H}|^2$, which means $\nabla \cdot \mathbf{S_R} = 0$ ($\mathbf{S_R}$ represents the real part of Poynting Vector), also indicating imcompressible. Together with $\mathbf{n} \cdot \mathbf{S_R} = 0$, the ideal power-flow condition also remains valid in lossless 2D ENZ media. The difference is that, since ε and μ are not equal in this case, the impedance-matching condition is no longer satisfied, leading to reduced transmission. In summary, these results demonstrate that variations in permeability do not affect the ideal-fluid characteristics of the power flow within the ENZ medium; they only modify the overall system transmittance.

We further validate the above conclusions through simulation. We consider geometry containing dead ends and obstacles. In the EMNZ medium ($\varepsilon \approx 0, \mu \approx 0$), the power flow exhibits ideal fluid characteristics and achieves nearly 100% transmittance, with both $\nabla \times \mathbf{S} = 0$ (irrotational) and $\nabla \cdot \mathbf{S} = 0$ (incompressible) satisfied, as shown in Fig. S6 (b, f, j, n, r). In contrast, the power flow in a conventional air medium is chaotic and disordered, as illustrated in Fig. S6 (a, e, i, m, q). Next, we examine an ideal ENZ medium with a dielectric constant of approximately zero ($\varepsilon \approx 0$), while the permeability is set to $\mu = 1$. According to Eqs. (S5) and (S6), the real parts of both the divergence and the curl of the power flow are predicted to be zero, satisfying the characteristics of an ideal fluid. The power flow indeed exhibits ideal-fluid behavior, as shown in Fig. S6 (c, g, k, o). However, since the permeability μ is not zero, the impedance of this ENZ medium at the ports is mismatched, resulting in nearly zero transmittance, as shown in Fig. S6(s). By adjusting the permeability dopant, the ENZ medium can achieve full transmission while maintaining ideal power flow, as shown in Fig. S6 (d, h, l, p, t). Fig. S7 shows the normalized power-flow distribution in the ENZ medium for different permeability values. As observed, in both ideal ENZ and permeability-doped ENZ media, variations in permeability do not affect the ideal-fluid behavior of the power flow within the medium; they only modify the overall system transmittance.

We also analyzed the impact of permeability losses on the EMNZ power flow. According to Eqs. (S5) and (S6), the presence of loss directly induces a non-zero divergence in the power flow, proportional to the imaginary part of the dielectric constant and permeability, which correlates with field attenuation. The effect of loss on the curl of the power flow is more indirect: as the magnetic field becomes non-uniform, analytical prediction becomes difficult. As shown in Fig. S8, non-zero divergence of the power flow increases with μ''. In contrast, the curl of the power flow remains largely robust, as permeability loss does not significantly affect the uniform distribution of the magnetic field in the EMNZ medium. This behavior is also discussed in Ref. 22.

Next, we examine the effect of the permittivity of the medium on the power flow. Eqs. (S4) and (S5) indicate that variations in the permittivity influence the homogeneity of the magnetic field within the medium, and consequently, the curl of the power flow. To illustrate this, we provide a numerical analysis of the frequency response of the power-flow distribution in the EMNZ medium, as shown in Fig. S9. The permittivity is modeled using a Drude dispersion to match the dispersion characteristics of the waveguide ENZ medium, where the waveguide plasma satisfies $\varepsilon_{\text{eff}} = 1 - (\lambda/2h)^2$. The permeability of the medium is described by a Lorentzian model, $\mu_{\text{eff}} = (f^2 - f_p^2)/(f^2 - f_0^2)$with $f_0 = 0.97 f_p$, to simulate photonic doping in the ENZ medium[7, 22]. As shown in Fig. S9, the system exhibits a degree of robustness to frequency variations, maintaining relatively stable ideal-fluid characteristics within a 7% bandwidth. When the frequency offset becomes too large ($\varepsilon \neq 0, \mu \neq 0$), noticeable turbulence and vortices appear.

In general, the permittivity of the medium follows Eqs. (S4) and (S5) and affect the curl and divergence of the power flow. In the system proposed in this manuscript, we construct an ENZ environment using the cutoff frequency of the waveguide. Frequency deviations from the cutoff frequency affect the dielectric constant of the medium, thus influencing the power flow. The permeability of the medium,

through doping modulation, only affects the transmittance of the system within the $\varepsilon \approx 0$ frequency band, without affecting the curl and divergence of the power flow. The presence of losses directly causes non-zero divergence in the power flow, proportional to the imaginary parts of the dielectric constant and/or permeability and can be correlated with field attenuation.

[R6] Liberal, I., Lobet, M., Li, Y., & Engheta, N. (2020). Near-zero-index media as electromagnetic ideal fluids. Proceedings of the National Academy of Sciences, 117(39), 24050-24054.Zhou, Z.,

[R7] Li, Y., Li, H., Sun, W., Liberal, I., & Engheta, N. (2019). Substrate-integrated photonic doping for near-zero-index devices. Nature communications, 10(1), 4132.

Reviewer 2 states**: "2. What objective is actually being minimized by the selected route? You introduce an effective branch 'resistance' R_S ~ a/b and an Ohm-like relation I_SR_S = Delta_phi with I_S = Sb and Delta_phi = phi_B - phi_A. In mazes with nonuniform widths and multiple competing routes, does the selected route minimize geometric length, minimize sum(a/b), or minimize a more complete effective impedance that includes junction/corner contributions (and possibly loss)? Can you provide a _quantitative_ benchmark comparing predicted route choice to a graph shortest-path computed with weights extracted from full-wave port-to-port admittance/impedance?"**

Our Response: We thank the reviewer for this important and highly insightful comment. In our framework, routing in EMNZ media is not governed simply by the geometrically shortest path, but rather by the path with the lowest effective impedance. Specifically, under the ideal or near-ideal EMNZ approximation, each branch behaves analogously to a resistive channel in a DC circuit, with an effective resistance that scales as: $R_S = a/b$, where a and b denote the branch length and width, respectively. For a route composed of multiple connected branches, the relevant quantity is therefore the total effective resistance, namely the sum of the resistances of all branches along that route. In this sense, the preferred route is the one that minimizes the overall effective resistance, rather than the geometric length alone.

It is particularly important in mazes with non-uniform widths and multiple competing routes. For example, a geometrically longer route may still be favored if it contains wider branches and thus has a lower total effective resistance. Conversely, a shorter but narrower route may carry less power. This behavior is fully consistent with the Ohm-like relation introduced in the manuscript, analogous to conventional resistive systems in which

resistance decreases with increasing cross-sectional area and increases with length.

To clarify this point quantitatively, we now provide a multi-path analysis in **Fig. R10**, which agrees well with the theoretical prediction:

(i) When the two paths have equal widths, the power carried by each path is inversely proportional to its length.

(ii) When the two paths have equal lengths, the power carried by each path is proportional to its width.

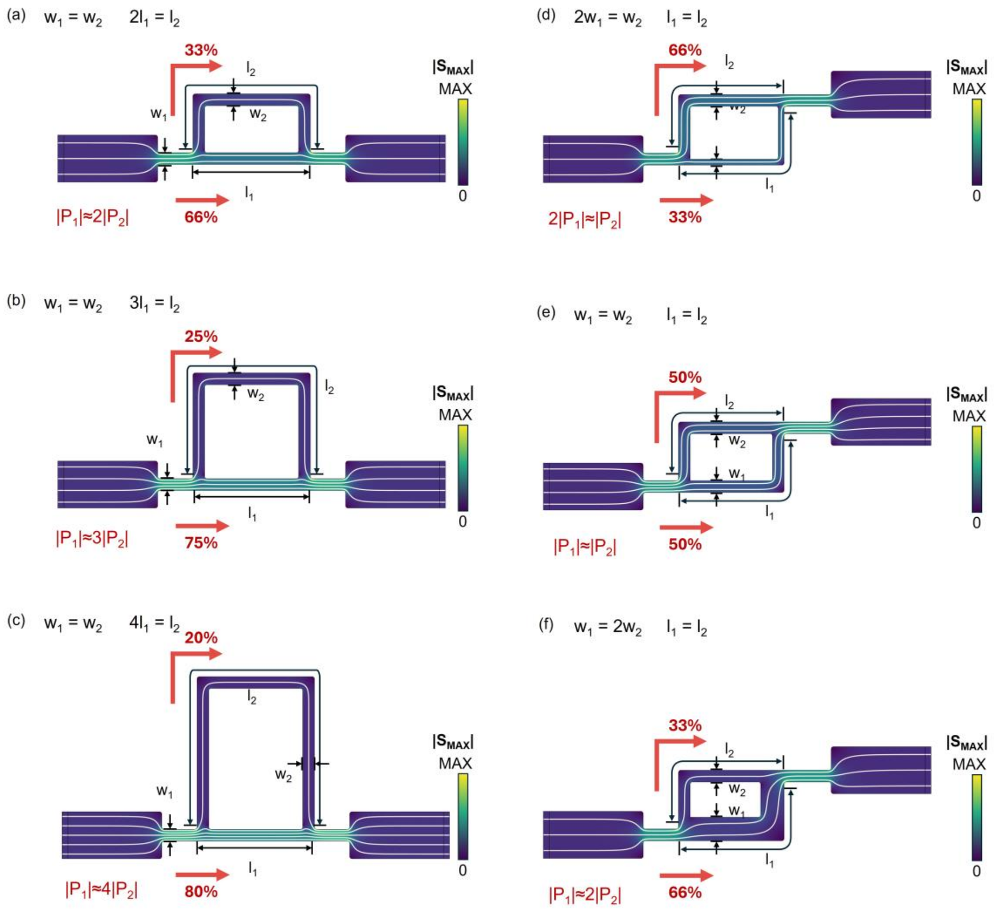

Fig. R10. Power-flow routing in EMNZ media under multi-path conditions. Two paths, labeled Channel 1 and Channel 2, are considered, with P_1 and P_2 denoting the total power transmitted along each path. a-c Both paths have equal widths but different lengths; the power along each path is inversely proportional to its length, with longer paths carrying less power. **d-f** Both paths have equal lengths but different widths; the power along each path is proportional to its width, with wider paths carrying more power.

At the same time, we identified a typographical error in the main text; the quantity corresponding to the current term in Ohm's law should be the Poynting vector S, rather than $I_S = \mathbf{S} / b$. Therefore, we have revised the corresponding equations in the manuscript.

$$\int_a \int_b \vec{S} dxdy = \phi_B - \phi_A \rightarrow b \int_a \vec{S} dy = \phi_B - \phi_A \quad (6)$$

$$S R_S = \Delta\phi \quad (7)$$

Ideal EM power	↔	DC circuit	↔	Ideal fluid
S	↔	Current I	↔	Q
$\Delta\phi$	↔	Voltage U	↔	ΔP
$R_S = a/b$	↔	Resistance R	↔	$8a\eta/\pi r^4$

(9)

Besides, based on this comment, we have revised the descriptions in the first paragraph of **Multi-port supercoupling and bus in millimeter and terahertz frequencies** and **Supplementary Fig. S11** as followings:

"We can also control the power distribution among the output ports by adjusting their widths, effectively tuning the impedance R_s associated with the power flow at each port, as illustrated in Fig. S10. In the presence of multiple effective paths, the power distribution between the two channels also follows Eq. (9), as illustrated in Fig. S11. These results show that the Ohm's law of ideal electromagnetic power flow provides a unified framework for understanding and designing multi-port supercoupling in complex NZI structures."

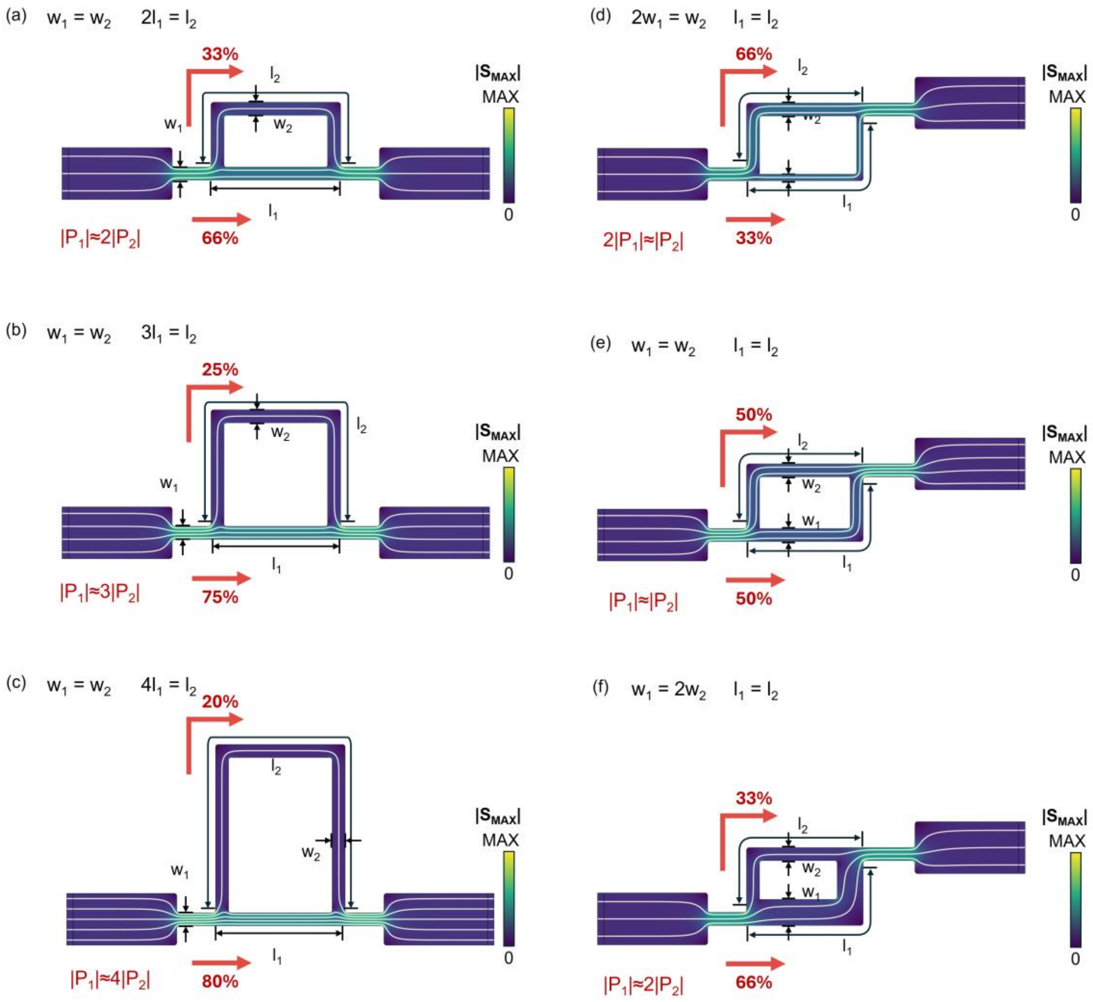

Supplementary Fig. S11 | Power-flow routing in EMNZ media under multi-path conditions. Two paths, labeled Channel 1 and Channel 2, are considered, with $P_1$and P_2denoting the total power transmitted along each path. a-c Both paths have equal widths but different lengths; the power along each path is inversely proportional to its length, with longer paths carrying less power. **d-f** Both paths have equal lengths but different widths; the power along each path is proportional to its width, with wider paths carrying more power. ”

***Reviewer 2 states*: “3. The experiment reconstructs the relative Re{S} map from measured magnetic fields through a mesh top plate. How sensitive are the inferred direction and magnitude of Re{S} to: (i) probe loading, (ii) finite mesh leakage/perturbation, and (iii) deviations from the assumed TE10 symmetry used to relate fields at z = h/2 to the midplane flow? An uncertainty budget (or calibration against a geometry with analytically predictable S) would strengthen the experimental claim.”**

Our Response: We sincerely thank the reviewer for this insightful comment. During testing, we employed a mesh top plate and small probe loops to measure the field strength. This approach has a negligible effect on the field distribution of the constructed EMNZ system. In the updated measurements, the mesh openings are 4 mm (0.02λ at 1.5 GHz), and the probe loops measure 4 mm × 6 mm ($0.02\lambda \times 0.03\lambda$ at 1.5 GHz). These compact structures exert minimal influence on the electromagnetic distribution within the system.

We further performed numerical simulations to verify this effect, as shown in Fig. R10. First， for finite mesh leakage, the transmission spectra of the two maze structures covered by a solid top plate are indicated by the blue curves in Figs. R10(c) and R10(d), while the transmission spectra for the maze structures with a mesh top plate are shown by the red curves. The presence of the mesh top plate has a negligible impact on the EM response of the system. Second, for probe loading, small probe loops were similarly placed at three representative locations, as illustrated in Fig. R10 (e). The calculated port-response spectra are shown in Figs. R10 (g)–(j), where the energy detected by the probes remains below –30 dB, indicating that the probes do not perturb the EM fields within the mazes. Third, for deviations from the assumed TE_{10} symmetry used to relate fields at z = h/2 to the midplane flow, Fig. R10 (f) shows the cross-sectional field distribution of the mazes in the waveguide EMNZ medium. The magnetic field across the section follows a cosine-like profile, consistent with Eq. (10) in the **Methods**. This indicates that the designed structure introduces negligible asymmetry to the TE_{10} mode distribution. Using the methodology described in Eqs. (10)–(15), the power-flow distribution at (z = h/2) within the mazes can thus be extracted and analyzed.
Eqs. (10)–(15) are as follows:

$$E(z) = cos(\frac{\pi z}{h})E(z = 0) \tag{10}$$

$$H(z) = \frac{1}{i\omega\mu_0} cos(\frac{\pi z}{h})(\nabla \times E(z = 0)) - \frac{\pi}{i\omega\mu_0 h} sin(\frac{\pi z}{h})\hat{z} \times E(z = 0) \tag{11}$$

$$E(z = 0) = \frac{i\omega\mu_0 h}{\pi} H(z = \frac{h}{2}) \times \hat{z} \tag{12}$$

$$S(z = 0) = E(z = 0) \times H(z = 0) = \frac{i\omega\mu_0 h}{\pi} H(z = \frac{h}{2}) \times \hat{z} \times H(z = 0) \tag{13}$$

$$S(z = 0) = -\frac{i\omega\mu_0 h}{\pi} H(z = 0)\left[H\left(z = \frac{h}{2}\right)(\hat{z} \cdot \hat{z}) - \hat{z}\left(\hat{z} \cdot H\left(z = \frac{h}{2}\right)\right)\right] = -\frac{i\omega\mu_0 h}{\pi} H(z = 0)H(z = \frac{h}{2}) \tag{14}$$

$$Re(S(z = 0)) \propto Re\left(H(z = \frac{h}{2})\right) \tag{15}$$

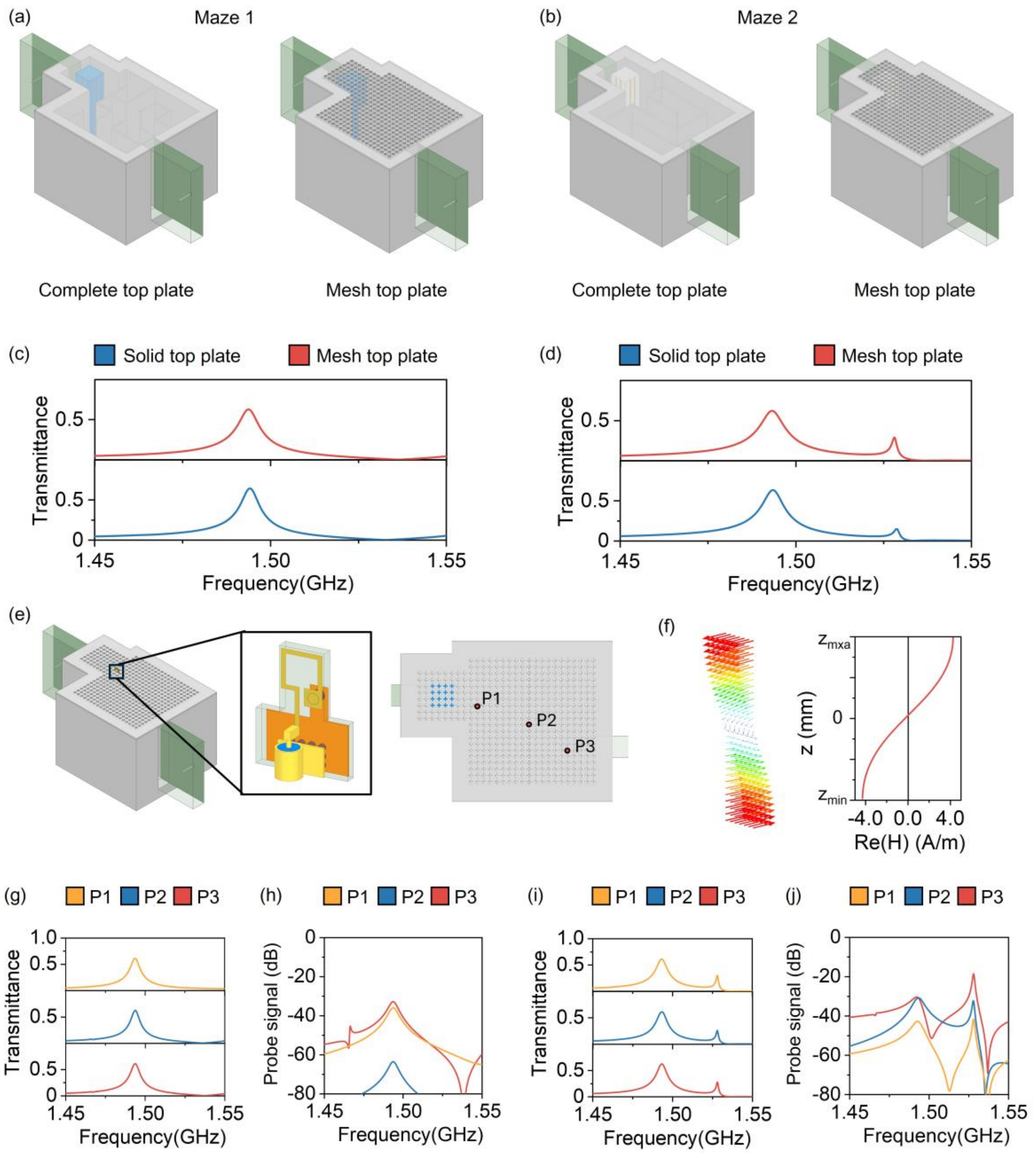

Fig. R10. Effect of test scenarios on waveguide EMNZ fields. (a–b) Schematic diagrams of the two maze configurations with a grid top plate and a solid top plate. (c) Influence of different top plates on the transmission spectrum for Maze 1. (d) Influence of different top plates on the transmission spectrum for Maze 2. (e) Schematic of probe signal detection, with three probe locations labeled P1, P2, and P3. (f) Schematic diagram of magnetic field distribution in the cross-section of the maze. (g) Transmission spectra of the Maze 1 system for probes at P1, P2, and P3. (h) Measured probe signals at P1, P2, and P3 in Maze 1. (i) Transmission spectra of the Maze 2 system for probes at P1, P2, and P3. (j) Measured probe signals at P1, P2, and P3 in Maze 2.

Based on this comment, we have revised the descriptions in the fourth paragraph of **Methods** and **Supplementary Fig. S3** as followings:

“The magnetic field probe of the experimental setup has a radius of 1.5 mm and is connected to one of the ports of the vector network analyzer Agilent N9971A via a 50 Ω coaxial line. The other port is connected to the input waveguide to excite the input EM waves. The probes and mesh top plate have been shown to exert a negligible impact on the fields within the maze, as illustrated in Fig. S3.”

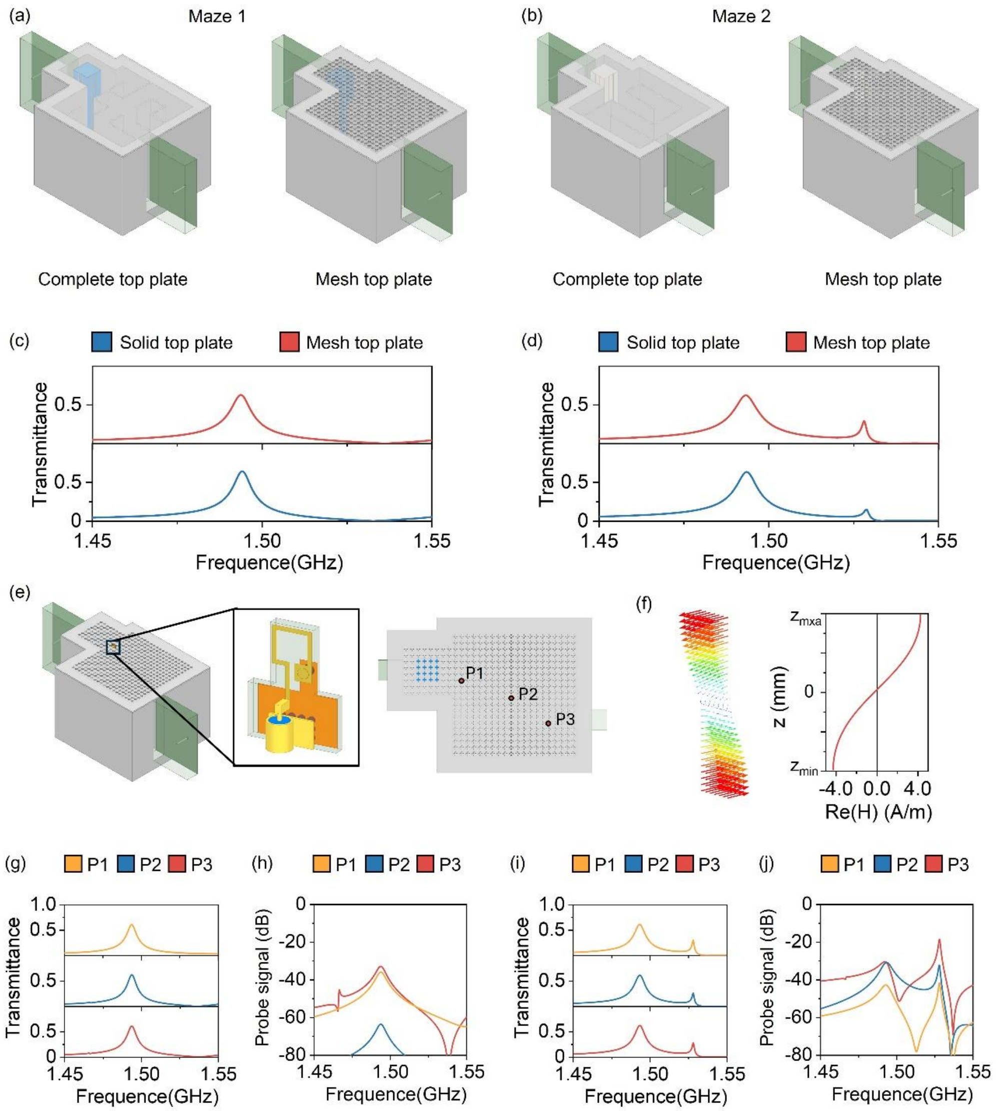

“

Supplementary Fig. S3 | Effect of test scenarios on waveguide EMNZ fields. a–b Schematic diagrams of the two maze configurations with a grid top plate and a solid top plate. **c** Influence of different top plates on the transmission spectrum for Maze 1. (d) Influence of different top plates on the transmission spectrum for Maze 2. **e** Schematic of probe signal detection, with three probe locations labeled P1, P2, and P3. **f** Schematic diagram of magnetic field distribution in the cross-section of the maze. **g** Transmission spectra of the Maze 1 system for probes at P1, P2, and P3. **h** Measured probe signals at P1, P2, and P3 in Maze 1. **i** Transmission spectra of the Maze 2 system for probes at P1, P2, and P3. **j** Measured probe signals at P1, P2, and P3 in Maze 2.”

Reviewer 2 states: **"4. How does the proposed NZI/EMNZ 'bus' compare to, and inter-operate with, other on-chip wave control and ENZ/NZI toolkits? You propose an extendable supercoupling / 'intelligence bus' for plug-and-play interconnects. Could you quantitatively position this against: (a) guided-wave-driven wavefront-control architectures, such as e.g. in Nature Communications 15, 5992 (2024), and (b) established ENZ/NZI building blocks including ENZ tunneling/supercoupling theory and experiments and EMNZ/ideal-fluid frameworks (e.g., Phys. Rev. Lett. 97, 157403 (2006); Phys. Rev. Lett. 100, 033903 (2008); Science 355, 1058-1062 (2017); PNAS 117, 24050-24054 (2020))? Specifically, can your maze/bus network serve as a low-loss feed network for guided-wave metasurfaces, and what are the bandwidth and insertion-loss tradeoffs versus a metasurface-native guided-wave distribution network?"**

Our Response: We thank the reviewer for this valuable comment. We agree that the proposed NZI/EMNZ "bus" should be positioned more clearly with respect to both guided-wave-driven wavefront-control platforms and the established ENZ/NZI literature.

First, compared with guided-wave-driven metasurface architectures, such as the recent work published in Nature Communications 15, 5992 (2024), our proposed EMNZ bus, although also concerned with electromagnetic-wave manipulation, is primarily focused on passive routing and interconnection. In Ref. R8, the guided-wave-driven metasurface is designed for arbitrary wavefront control, in which guided waves are progressively converted into free-space radiation and shaped into prescribed beams; its emphasis is therefore on radiation engineering and aperture synthesis. In contrast, our structure is intended mainly as a passive routing/interconnect platform, in which electromagnetic power is redistributed among ports inside a shared NZI network, while exhibiting strong tolerance to geometric deformation. In this sense, the two approaches should be viewed as complementary rather than competing: the EMNZ bus can provide a low-phase-delay feeding/routing layer, whereas the guided-wave metasurface can provide the final wavefront shaping and radiation functionality. This distinction has now been clarified in the revised manuscript.

Second, with respect to the existing ENZ/NZI report, our work is conceptually built upon and extends several established foundations, including ENZ tunneling/supercoupling theory and experiments, photonic doping, and the ideal-fluid interpretation of NZI power flow. The early ENZ supercoupling studies demonstrated that electromagnetic energy can be squeezed through narrow ENZ channels, with transmission that is largely insensitive to channel geometry [R9]. Subsequent works experimentally verified this phenomenon,

introduced photonic doping as a means to tailor the effective constitutive response of ENZ media [R10,R11], and further showed that power flow in NZI media can be interpreted in terms of an ideal-electromagnetic-fluid picture [R12]. Our contribution is to move beyond isolated point-to-point channels and demonstrate multi-branch routing and multi-port redistribution in complex network geometries, together with an experimentally implemented EMNZ platform and a system-level "bus" interpretation. We have revised the Introduction and Discussion sections to make the relationship to prior ENZ/NZI work much more explicit.

We also agree that the proposed maze/bus network can, in principle, serve as a low-loss feeding network for guided-wave metasurfaces or other on-chip wave-control devices, provided that the two subsystems operate in a compatible frequency regime. In such a hybrid architecture, the EMNZ bus would be responsible for signal routing, splitting, and port-to-port delivery, while the downstream metasurface or leaky-wave structure would handle wavefront extraction and beam formation. As long as the photonic dopant remains sufficiently low-loss, the proposed system can maintain low insertion loss; indeed, the low-loss potential of related photonic-doping-based platforms has already been demonstrated in prior work.

Finally, regarding the bandwidth and insertion-loss tradeoff, we agree with the reviewer that this is an important issue. At present, our cutoff-based EMNZ implementation is inherently a near-cutoff, finite-bandwidth platform. Therefore, its primary advantages are not broadband operation or minimum loss over a wide frequency range, but rather geometric tolerance, suppressed phase accumulation, and impedance-dominated routing near the EMNZ operating point.

Based on this comment, we have revised the descriptions in the third paragraph of **Introduction** and the second paragraph of **Multi-port supercoupling and bus in millimeter and terahertz frequencies** as followings:

"In this work, we extend NZI supercoupling from isolated channels to complex, multi-branch EM networks, and systematically investigate how EM power distributed and routed boundary-conditioned and impedance-dominated transport. Specifically, we demonstrate that in maze-like structures composed of PECs and filled with NZI media, EM power flow injected at an input port is deterministically redirected toward impedance-matched output ports, while power transport into PEC-terminated dead-end branches is intrinsically suppressed[22,23]. This multi-ports supercoupling[24-27] behavior is validated through both full-wave numerical simulations and measurements. By contrast, in geometrically identical structures filled with vacuum, EM power flow exhibits strong sensitivity to

geometry, leading to inefficient energy transport and negligible transmission to the output. The routing behavior is arisen from quasi-static, boundary-conditioned EM transport in NZI media[28-30], and can be further analogized to pressure-driven water flow in maze-like networks and to current distribution in direct-current circuits, enabling the description of EM power flow intensity through an Ohm's-law-like framework. Benefiting from routing in NZI networks, near-total transmission of energy between arbitrary input and output ports can be achieved through any input-output engineering in complex system. This opens the door to implementing a "bus" architecture in optics and microwave circuit[31,32], enabling the integration of plug-and-play devices for future millimeter-wave (mmW) and terahertz (THz) applications, with significant implications for inter-device communication and routing management. "

"Unlike previous ENZ/NZI studies that focus on point-to-point supercoupling, this bus extends the paradigm to multi-branch routing and multi-port power redistribution governed by an Ohm's law of ideal EM fluids[8,22,24-26]. Besides, Unlike guided-wave-driven metasurfaces, which focus on wavefront shaping and radiation, the proposed EMNZ bus is designed for passive routing and interconnection of electromagnetic power[46]."

[R8] Li, S., Tsakmakidis, K. L., Jiang, T., Shen, Q., Zhang, H., Yan, J., ... & Shen, L. (2024). Unidirectional guided-wave-driven metasurfaces for arbitrary wavefront control. Nature Communications, 15(1), 5992.

[R9] Silveirinha, M., & Engheta, N. (2006). Tunneling of Electromagnetic Energy through Subwavelength Channels and Bends using ε-Near-Zero Materials. *Physical review letters*, *97*(15), 157403.

[R10] Liberal, I., Mahmoud, A. M., Li, Y., Edwards, B., & Engheta, N. (2017). Photonic doping of epsilon-near-zero media. *Science*, *355*(6329), 1058-1062.

[R11] Edwards, B., Alù, A., Young, M. E., Silveirinha, M., & Engheta, N. (2008). Experimental Verification of Epsilon-Near-Zero Metamaterial Coupling and Energy Squeezing Using a Microwave Waveguide. *Physical review letters*, *100*(3), 033903.

[R12] Liberal, I., Lobet, M., Li, Y., & Engheta, N. (2020). Near-zero-index media as electromagnetic ideal fluids. *Proceedings of the National Academy of Sciences*, *117*(39), 24050-24054.

Reviewer 2 states**: "5. The authors' simulations state that the effect is 'inherently frequency-independent,' but the EMNZ experimental platform relies on TE10 cutoff and dopant tuning, which are necessarily dispersive. What is the measured operational bandwidth (e.g., 3 dB transmission band and a 'route selectivity' metric band), and how does route selectivity degrade as n departs from near-zero, or as loss increases? A compact scaling law or parametric sweep versus Re(n), Im(n), and maze-junction density would be helpful in translating the concept to mmW/THz/optical implementations."**

Our Response: We thank the reviewer for this insightful question. We agree that the EMNZ experimental platform relies on the TE_{10} cutoff and doping-based tuning, which results in a limited transmission bandwidth. For example, in the two maze structures discussed in the

manuscript, the -3 dB transmission bandwidths are 5.1% and 5.0%, respectively, as shown in Fig. S2. Consequently, full transmission occurs only within a narrow frequency range. However, the ideal-fluid behavior of the power flow in the medium exhibits greater frequency robustness. As previously discussed, Eqs. (R1) and (R2) indicate that when either the permittivity or the permeability approaches zero, the power flow behaves as an ideal fluid. In the waveguide ENZ platform presented in this manuscript, the near-zero permittivity provides ideal power-flow characteristics, while permeability tuning is used solely to adjust the system's transmission.

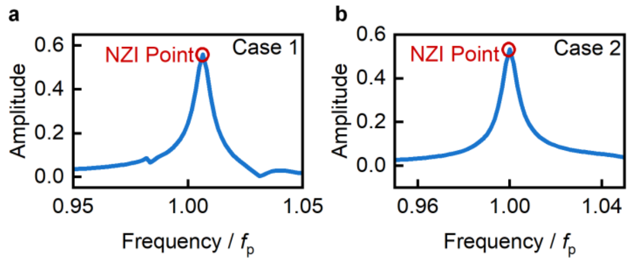

Supplementary Fig. S2 | Measured transmission amplitude of spontaneous escape of light from NZI mazes in Fig. 3 in the main text. a Case 1. **b** Case 2. Both measured frequencies are near the central frequency f_p = 3.04 GHz (see Materials and methods).

$$\nabla \cdot \mathbf{S} = -\frac{\omega}{2}[(\varepsilon_0\varepsilon''|\mathbf{E}|^2 + \mu_0\mu''|\mathbf{H}|^2) + i(\varepsilon_0\varepsilon'|\mathbf{E}|^2 + \mu_0\mu'|\mathbf{H}|^2)] \qquad \text{(R1)}$$

$$\nabla \times \mathbf{S} = -\frac{1}{2}[(\mathbf{H}^* \cdot \nabla)\mathbf{E} - (\mathbf{E} \cdot \nabla)\mathbf{H}^* + \mathbf{E}(\nabla \cdot \mathbf{H}^*) - \mathbf{H}^*(\nabla \cdot \mathbf{E})] \qquad \text{(R2)}$$

In comparison, the waveguide ENZ platform demonstrates superior frequency stability. As shown in Fig. R10 (i), the platform's effective permittivity remains within ±0.2 over a 20% bandwidth. Figs. R11 (a)–(g) illustrates the robustness of the power flow under frequency detuning, showing that the ideal-fluid characteristics are preserved over approximately a 7% frequency range. When the frequency deviation becomes too large ($\varepsilon \neq 0, \mu \neq 0$), pronounced turbulence and vortices emerge in the power flow. Based on Figs. R10 and R6, we draw the following conclusions: In the waveguide ENZ platform, when the permittivity remains within ±0.1, the power flow exhibits quasi–ideal fluid behavior, with negligible vortices or turbulence. The permeability primarily serves to tune the system's transmission. Regarding the issue of loss, as discussed in our response to your Comment 1, losses introduce a non-zero divergence in the power flow but have minimal impact on its curl.

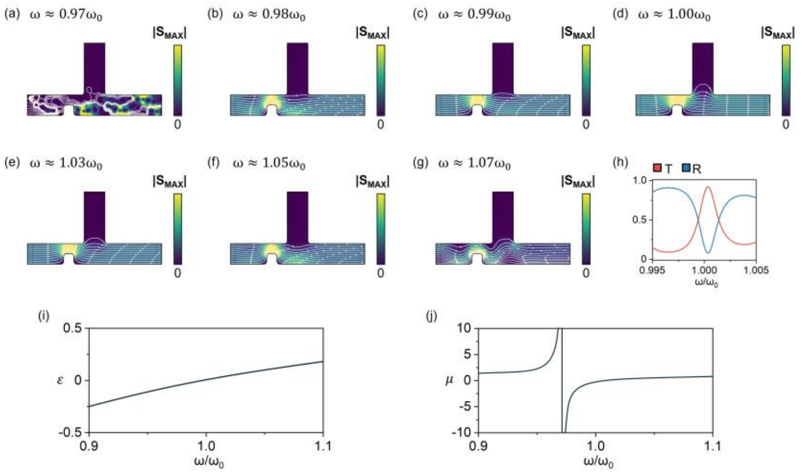

Fig. R11. Frequency response of electromagnetic power-flow distribution in the ENZ medium. Schematic diagrams of the power-flow distribution at different frequencies: (a) $\omega = 0.97\ \omega_0$, (b) $\omega = 0.98\ \omega_0$, (c) $\omega = 0.99\ \omega_0$, (d) $\omega = 1.0\ \omega_0$, (e) $\omega = 1.02\ \omega_0$, (f) $\omega = 1.05\ \omega_0$, (g) $\omega = 1.07\ \omega_0$; (h) corresponding transmission spectrum of the ENZ medium. (i) and (j) Equivalent ε μ spectrum of the medium.

Based on this comment, we have revised the descriptions in the first paragraph of **Experimental verifications** and **Supplementary Fig. S9** as followings:

"In other words, within the ideal EMNZ approximation ($\varepsilon_h = 0$, $\mu_h = 0$), the Poynting vector satisfies the conditions of a harmonic field inside NZI media. For the case in non-ideal conditions, please refer to Supplementary Note 4."

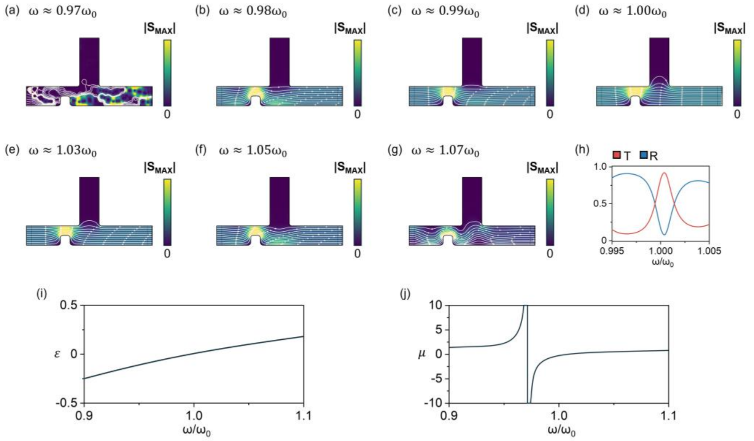

Supplementary Fig. S9 | Frequency response of electromagnetic power-flow distribution in the ENZ medium. Schematic diagrams of the power-flow distribution at different frequencies. a $\omega = 0.97\ \omega_0$, **b** $\omega = 0.98\ \omega_0$, **c** $\omega = 0.99\ \omega_0$, **d** $\omega = 1.00\ \omega_0$, **e** $\omega = 1.02\ \omega_0$, **f** $\omega = 1.05\ \omega_0$, **g** $\omega = 1.07\ \omega_0$. **h** corresponding transmission spectrum of the ENZ medium. **i** and **j** Equivalent ε and μ spectrum of the medium.

Response to Reviewer #3:

***Reviewer 3 states*: "*Summary: This paper presents a fascinating electromagnetic analogue to the fluid dynamics phenomenon where pressure-driven water spontaneously solves mazes. By utilizing Near-Zero-Index (NZI) media, the authors demonstrate—both theoretically and experimentally—that light can "spontaneously escape" complex maze structures by finding the optimal path and rejecting erroneous turns without external guidance. The work derives an "Ohm's law for EM power flow," linking potential differences to energy propagation, and proposes a practical application in the form of an "extendable supercoupling" bus for photonic and millimeter-wave circuits. While the core concept is novel and the potential applications for geometry-agnostic routing are promising, I have several specific questions and concerns regarding the experimental validation, simulation details, and robustness of the proposed mechanism.*"**

Our Response: We thank the Reviewer for the careful reading of our manuscript and for the concise and accurate summary of our work. We address the specific comments and suggestions in detail below.

***Reviewer 3 states*: "*1. Mechanism and Multi-path Behavior (Figure 1) (1) Multiple Valid Paths: The current demonstration focuses on scenarios with a single "correct" exit. What happens if there is more than one valid path to an exit? Does the energy split equally, or does it favor the topologically shorter path? (2) Switching Capabilities: Is it possible to tune the system dynamically to select between multiple valid paths, effectively acting as an optical switch? Expanding on this could significantly enhance the practical relevance of the work.*"**

Our Response: We thank the reviewer for this insightful comment. In NZI-mediated routing, the redistribution of electromagnetic power is primarily governed by the effective impedance of the output ports and the geometry of the branches. Specifically, under near-ideal EMNZ conditions, the Poynting vector satisfies.

$$\mathbf{S} \propto \nabla\phi \qquad \text{(R9)}$$

In this framework, each branch behaves analogously to a resistive channel in a DC circuit, with its effective resistance proportional to $R_S \sim a/b$, where a and b denote the length and width of the branch, respectively, as discussed in our main text. Consequently, when multiple effective paths exist, energy is evenly distributed if the output ports are identical. If the paths have different effective resistances, energy distribution follows a rule similar to Ohm's law, favoring paths with lower effective resistance. This behavior is demonstrated

in Fig. S10 of the revised manuscript. Fig. S10 shows the case of multiple output ports being simultaneously activated. When the output port widths are identical, the input power is approximately evenly distributed among them, consistent with the effective-circuit interpretation. Minor reflections observed at the outputs arise from impedance mismatches between the input port (input width) and the output ports (output widths).

We also thank the reviewer for the suggestion regarding dynamic switching capabilities. In principle, the routing behavior in NZI networks can indeed be dynamically controlled by modifying the impedance conditions at the ports or branches, which directly alters the effective potential distribution governing the power flow. Several approaches can be used to achieve such dynamic switching. For instance, in the microwave regime, switches such as PIN diodes can be employed to control the connectivity of ports or branches. In the optical regime, tunable materials—such as phase-change materials [R13] or electro-optic materials [R14,R15]—can similarly be used to redirect the power flow.

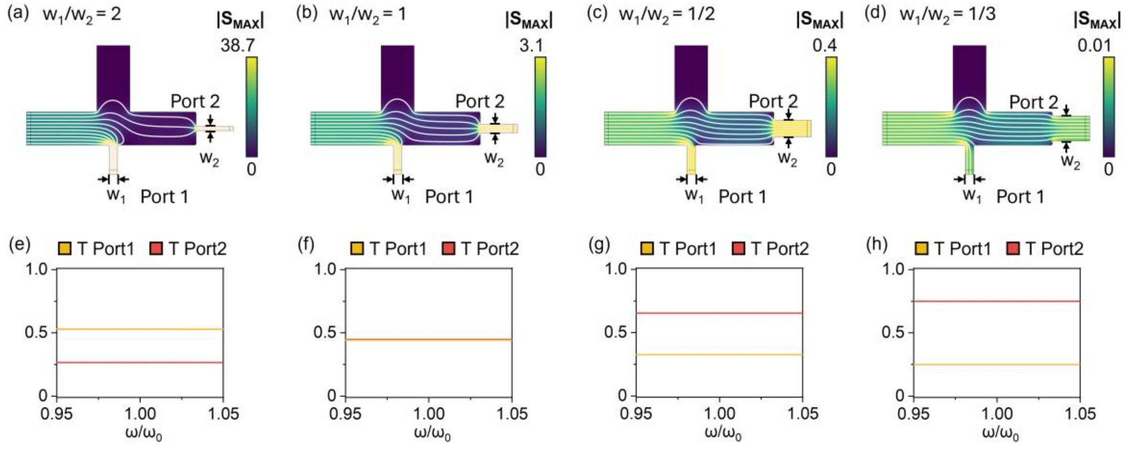

Fig. R11. Power-flow distribution in EMNZ media with multiple output ports. (a)-(b) Schematic illustrations of the power-flow distribution for different output-port width ratios. (e)-(h) Corresponding transmission spectra for the system under the same output-port width ratios.

Based on this comment, we have revised the descriptions in the first paragraph of **Multi-port supercoupling and bus in millimeter and terahertz frequencies** and **Supplementary Fig. S10** as followings:

"We can also control the power distribution among the output ports by adjusting their widths, effectively tuning the impedance R_s associated with the power flow at each port, as illustrated in Fig. S10. In the presence of multiple effective paths, the power distribution between the two channels also follows Eq. (9), as illustrated in Fig. S11. These results show that the Ohm's law of ideal electromagnetic power

flow provides a unified framework for understanding and designing multi-port supercoupling in complex NZI structures."

[R13] King, J., Wan, C., Park, T. J., Deshpande, S., Zhang, Z., Ramanathan, S., & Kats, M. A. (2024). Electrically tunable VO2–metal metasurface for mid-infrared switching, limiting and nonlinear isolation. Nature Photonics, 18(1), 74-80.

[R14] Sisler, J., Thureja, P., Grajower, M. Y., Sokhoyan, R., Huang, I., & Atwater, H. A. (2024). Electrically tunable space–time metasurfaces at optical frequencies. *Nature Nanotechnology*, *19*(10), 1491-1498.

[R15] Nakamura, S., Sekiya, K., Matano, S., Shimura, Y., Nakade, Y., Nakagawa, K., ... & Maki, H. (2022). High-speed and on-chip optical switch based on a graphene microheater. ACS nano, 16(2), 2690-2698.

***Reviewer 3 states*: "2. Simulation Consistency and Robustness (Figure 2)**

1) Intensity Discontinuities: In Figures 2c and 2e, there appear to be discontinuities in the wave path intensity. Theoretically, a continuous flow should conserve energy flux across cross-sections (ignoring loss). The visual representation suggests non-uniformity. Could the authors clarify what causes these intensity variations? Are they simulation artifacts or physical effects of the NZI medium?"

Our Response: We thank the reviewer for this insightful comment and for pointing out the apparent discontinuities in the path intensity observed in Fig. 2(c) and (e). These variations do not arise from simulation errors; rather, they arise from the local reduction in intensity caused by channel widening under the condition of conserved total power.

In our configuration, the power flow density ($\boldsymbol{S}$, i.e., the magnitude of the Poynting vector) is inversely proportional to the local cross-sectional area of the path. In other words, when a channel widens, the same total power is distributed over a larger area, resulting in a reduced local intensity; conversely, narrower channels yield higher local intensities. Importantly, despite these variations in flux density, the electromagnetic power remains strongly confined to the impedance-optimal paths (i.e., those with the lowest effective resistance, $R_s = a/b$), and power entering dead-end or severely mismatched branches is effectively suppressed.

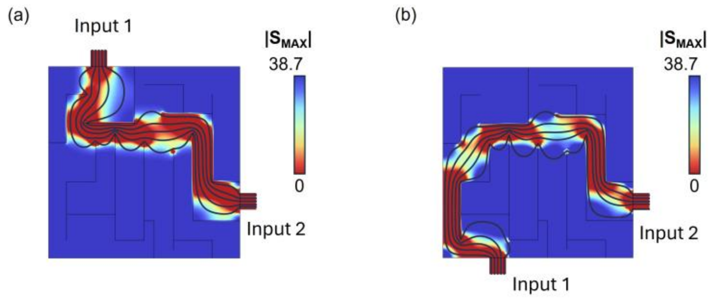

Fig. R12. Power flow streamline diagram of the maze in the manuscript, where the density of the streamlines represents the magnitude of the power flow.

Therefore, the observed intensity "discontinuities" are fully consistent with the ideal electromagnetic fluid model, and power redistribution within the network follows Ohm-like rules. Fig. R12 provides supplementary power-flow streamline diagrams corresponding to Fig. 2 of the manuscript. The electromagnetic power in the EMNZ medium is confined to the "correct paths," while in wider regions or at intersections, the power flow slightly diverges.

In the revised manuscript, revised the descriptions in the first paragraph of **Simulation verifications** as followings:

"The decrease in power flow at the intersection arises from the local widening of the passageway. This behavior is qualitatively analogous to pressure-driven flow redistribution in fluidic mazes, as illustrated in Fig. 1(a) and Extended Data Movie 1. For comparison, the same mazes filled with vacuum (n_h = 1) are simulated in Fig. 2(d) and Fig. 2(f)."

***Reviewer 3 states*: "*2) Sensitivity to Index Variations: How critical is the exact refractive index value (n ≈ 0)? What happens if there is a small deviation or perturbation in the index of the NZI medium? A sensitivity analysis showing how robust the routing is to small changes in n would strengthen the paper.*"**

Our Response: We thank the reviewer for this insightful comment. We next examine the effect of variations in the permittivity and permeability on the electromagnetic power flow. Specifically, for power flow within a medium, the divergence and curl of the Poynting vector can be expressed as:

$$\nabla \cdot \mathbf{S} = -\frac{\omega}{2}[(\varepsilon_0\varepsilon''|\mathbf{E}|^2 + \mu_0\mu''|\mathbf{H}|^2) + i(\varepsilon_0\varepsilon'|\mathbf{E}|^2 + \mu_0\mu'|\mathbf{H}|^2)] \quad \text{(R10)}$$

$$\nabla \times \mathbf{S} = -\frac{1}{2}[(\mathbf{H}^* \cdot \nabla)\mathbf{E} - (\mathbf{E} \cdot \nabla)\mathbf{H}^* + \mathbf{E}(\nabla \cdot \mathbf{H}^*) - \mathbf{H}^*(\nabla \cdot \mathbf{E})] \quad \text{(R11)}$$

where the medium parameters are given by $\varepsilon = \varepsilon' - i\varepsilon''$and $\mu = \mu' - i\mu''$. Eqs. (R6) and (R7) indicate that if either the permittivity or the permeability is zero, the divergence and curl of the power flow vanish. This is confirmed in Fig. R13: when the ε is zero, the μ does not affect the ideal-fluid characteristics of the power flow and serves only to tune the system's transmission. Cases with different loss levels are shown in Fig. R14; losses introduce non-zero divergence in the power flow but have minimal effect on the curl.

Furthermore, when waveguide ENZ dispersion is considered, the power-flow distribution is illustrated in Fig. R15. The permittivity is modeled using a Drude dispersion to match the waveguide ENZ medium, where the waveguide plasma satisfies $\varepsilon_{\text{eff}} = 1 - (\lambda/2h)^2$. The permeability is described by a Lorentzian model, $\mu_{\text{eff}} = (f^2 - f_p^2)/(f^2 - f_0^2)$with $f_0 = 0.97f_p$, to simulate photonic doping within the ENZ medium [R16, R17]. Based on Figs. R13–R15, we conclude that in the waveguide ENZ platform, when the permittivity remains within ±0.1, the power flow exhibits quasi-ideal fluid behavior, with negligible vortices or turbulence. The corresponding frequency range spans approximately 7%, while the permeability primarily serves to tune the system's transmission. Losses introduce a non-zero divergence in the power flow, but their effect on the curl is minimal.

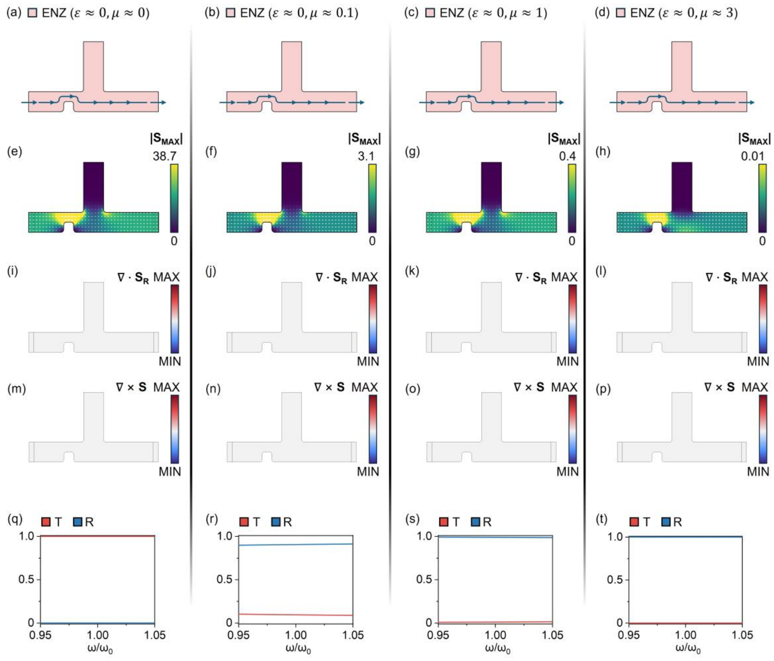

Fig. R13. Power-flow routing of electromagnetic waves in ENZ media with different permeabilities. (a–d) Schematic diagrams of the structures with ENZ media of varying magnetic permeability. (e–h) Corresponding power-flow distributions in each medium, where white arrows indicate the direction of power flow. (i–l) Distributions of the real part of the power-flow divergence for each permeability. (m–p) Distributions of the real part of the power-flow curl for each permeability. (q–t) Transmission spectra of electromagnetic waves in ENZ media with different permeabilities.

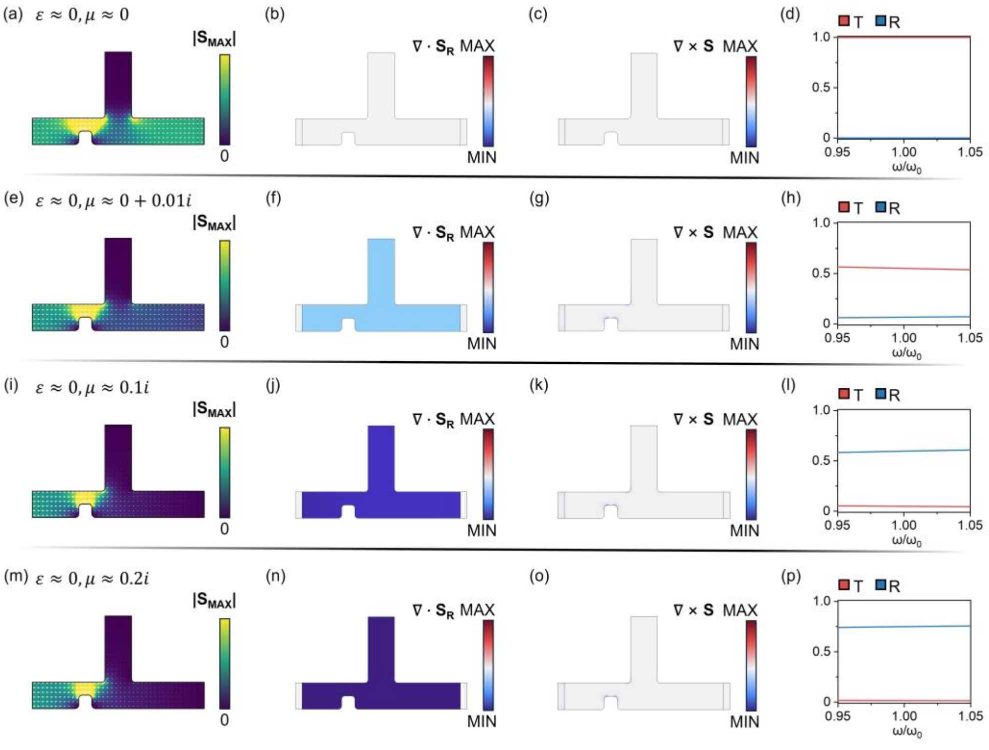

Fig. R14. Schematic diagram of power-flow routes for electromagnetic waves in the ENZ medium under different permeability losses. (a)–(d) Power flow distributions and transmission spectra for $\mu'' = 0$. (e)–(h) Power flow distributions and transmission spectra for $\mu'' = 0.01$. (i)–(l) Power flow distributions and transmission spectra for $\mu'' = 0.1$. (m)–(p) Power flow distributions and transmission spectra for $\mu'' = 0.2$.

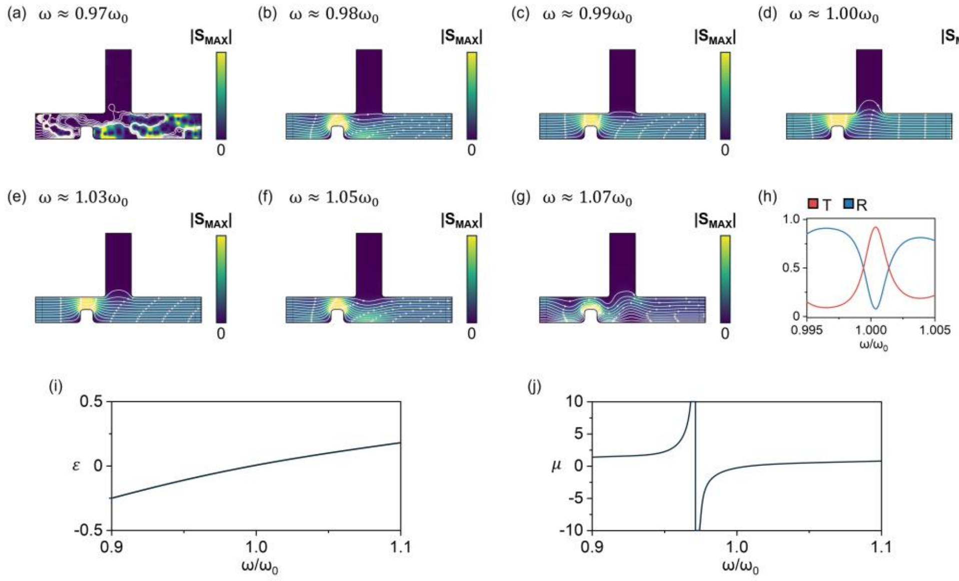

Fig. R15. Frequency response of electromagnetic power-flow distribution in the ENZ medium. Schematic diagrams of the power-flow distribution at different frequencies: (a) $\omega = 0.97\,\omega_0$, (b) $\omega = 0.98\,\omega_0$, (c) $\omega = 0.99\,\omega_0$, (d) $\omega = 1.0\,\omega_0$, (e) $\omega = 1.02\,\omega_0$, (f) $\omega = 1.05\,\omega_0$, (g) $\omega = 1.07\,\omega_0$; (h) corresponding transmission spectrum of the ENZ medium. (i) and (j) Equivalent ε μ spectrum of the medium.

Based on this comment, we have revised the descriptions in the fourth paragraph of **Theoretical Analysis** and **Supplementary Note 4** as followings:

"In other words, within the ideal EMNZ approximation ($\varepsilon_h = 0$, $\mu_h = 0$), the Poynting vector satisfies the conditions of a harmonic field inside NZI media. For the case in non-ideal conditions, please refer to Supplementary Note 4."

"**Supplementary Note 4. Electromagnetic Power Flow in Nonideal EMNZ Media**

In our main text, the theoretical assumptions of ideal fluids, namely $\nabla \times \mathbf{S} = 0$(irrotational) and $\nabla \cdot \mathbf{S} = 0$(incompressible), are idealized. However, in practical applications, particularly on waveguide EMNZ platforms, various non-ideal factors can lead to deviations from these assumptions. Here, we present a quantitative analysis of the robustness of this ideal medium, that is, an analysis of the EMNZ under non-ideal conditions. Specifically, we consider the EM power flow in a medium whose Poynting vector divergence and curl can be described as:

$$\nabla \cdot \mathbf{S} = -\frac{\omega}{2}[(\varepsilon_0\varepsilon''|\mathbf{E}|^2 + \mu_0\mu''|\mathbf{H}|^2) + i(\varepsilon_0\varepsilon'|\mathbf{E}|^2 + \mu_0\mu'|\mathbf{H}|^2)] \quad \text{(S5)}$$

$$\nabla \times \mathbf{S} = -\frac{1}{2}[(\mathbf{H}^* \cdot \nabla)\mathbf{E} - (\mathbf{E} \cdot \nabla)\mathbf{H}^* + \mathbf{E}(\nabla \cdot \mathbf{H}^*) - \mathbf{H}^*(\nabla \cdot \mathbf{E})] \quad \text{(S6)}$$

Here, the relative permittivity and permeability of the media are expressed as $\varepsilon = \varepsilon' - i\varepsilon''$and $\mu = \mu' - i\mu''$. From Eq. (S5), for any ideal lossless media ($\varepsilon'', \mu'' \approx 0$), the real part of $\nabla \cdot \mathbf{S}$ is zero, indicating incompressible. This result simply indicates that there is no power sink in a lossless EMNZ medium. For Eq. (S6), when either ε or μ is zero, it can be readily concluded that the curl of the Poynting vector is zero, indicating irrotational. According to boundary condition, $\mathbf{n} \cdot \mathbf{S}$ is naturally satisfied, indicating inviscid. These three conditions are the characteristics of ideal fluid, which also explains the routing behavior of EM waves in the maze.

For practical applications, however, a truly lossless EMNZ cannot be realized. Considering the EM field conditions in the waveguide-emulated plasmonic ENZ media[35,36], ENZ is emulated by the TE_{10} mode near the cutoff frequency. At this time, we have $\mathbf{H} = \mathbf{z}H_z$ and $\mathbf{E} = \mathbf{x}E_x + \mathbf{y}E_y = (-i\omega\varepsilon_0\varepsilon)^{-1}\nabla H_z \times \mathbf{z}$. Accordingly, Eq (S6) can be rewritten as:

$$\nabla \times \mathbf{S} = \frac{1}{2}(\mathbf{E} \cdot \nabla)\mathbf{H}^* = \frac{1}{2}\left(\mathbf{x}E_x + \mathbf{y}E_y\right) \cdot \nabla \cdot \mathbf{z}H_z \quad \text{(S7)}$$

For Eq. (S7), the curl of the Poynting vector depends solely on the homogeneity of the magnetic field within the ENZ medium.When $\varepsilon \approx 0$ in the ENZ medium, the magnetic field is uniformly distributed, and therefore $\nabla \times \mathbf{S} = 0$, indicating irrotational. Besides, according to Eq. (S5), for any lossless 2D ENZ medium with $\varepsilon \approx 0$ and $\mu = \mu' \neq 0$, $\nabla \cdot \mathbf{S} = i\mu_0\mu'|\mathbf{H}|^2$, which means $\nabla \cdot \mathbf{S}_R = 0$ ($\mathbf{S}_R$ represents the real part of Poynting Vector), also indicating imcompressible. Together with $\mathbf{n} \cdot \mathbf{S}_R = 0$, the ideal power-flow condition also remains valid in lossless 2D ENZ media. The difference is that, since ε and μ are not equal in this case, the impedance-matching condition is no longer satisfied, leading to reduced transmission. In summary, these results demonstrate that variations in permeability do not affect the ideal-fluid characteristics of the power flow within the ENZ medium; they only modify the overall system transmittance.

We further validate the above conclusions through simulation. We consider geometry containing dead ends and obstacles. In the EMNZ medium ($\varepsilon \approx 0, \mu \approx 0$), the power flow exhibits ideal fluid characteristics and achieves nearly 100% transmittance, with both $\nabla \times \mathbf{S} = 0$ (irrotational) and $\nabla \cdot \mathbf{S} = 0$ (incompressible) satisfied, as shown in Fig. S6 (b, f, j, n, r). In contrast, the power flow in a conventional air medium is chaotic and disordered, as illustrated in Fig. S6 (a, e, i, m, q). Next, we examine an ideal ENZ medium with a dielectric constant of approximately zero ($\varepsilon \approx 0$), while the permeability is set to $\mu = 1$. According to Eqs. (S5) and (S6), the real parts of both the divergence and the curl of the power flow are predicted to be zero, satisfying the characteristics of an ideal fluid. The power flow indeed exhibits ideal-fluid behavior, as shown in Fig. S6 (c, g, k, o). However, since the permeability μ is not zero, the impedance of this ENZ medium at the ports is mismatched, resulting in nearly zero transmittance, as shown in Fig. S6(s). By adjusting the permeability dopant, the ENZ medium can achieve full transmission while maintaining ideal power flow, as shown in Fig. S6 (d, h, l, p, t). Fig. S7 shows the normalized power-flow distribution in the ENZ medium for different permeability values. As observed, in both ideal ENZ and permeability-doped ENZ media, variations in permeability do not affect the ideal-fluid behavior of the power flow within the medium; they only modify the overall system transmittance.

We also analyzed the impact of permeability losses on the EMNZ power flow. According to Eqs. (S5) and (S6), the presence of loss directly induces a non-zero divergence in the power flow, proportional to the imaginary part of the dielectric constant and permeability, which correlates with field attenuation. The effect of loss on the curl of the power flow is more indirect: as the magnetic field becomes non-uniform, analytical prediction becomes difficult. As shown in Fig. S8, non-zero divergence of the power flow increases with μ''. In contrast, the curl of the power flow remains largely robust, as permeability loss does not significantly affect the uniform distribution of the magnetic field in the EMNZ medium. This behavior is also discussed in Ref. 22.

Next, we examine the effect of the permittivity of the medium on the power flow. Eqs. (S4) and (S5) indicate that variations in the permittivity influence the homogeneity of the magnetic field within the medium, and consequently, the curl of the power flow. To illustrate this, we provide a numerical analysis of the frequency response of the power-flow distribution in the EMNZ medium, as shown in Fig. S9. The permittivity is modeled using a Drude dispersion to match the dispersion characteristics of the waveguide ENZ medium, where the waveguide plasma satisfies $\varepsilon_{\text{eff}} = 1 - (\lambda/2h)^2$. The permeability of the medium is described by a Lorentzian model, $\mu_{\text{eff}} = (f^2 - f_p^2)/(f^2 - f_0^2)$with $f_0 = 0.97 f_p$, to simulate photonic doping in the ENZ medium[7, 22]. As shown in Fig. S9, the system exhibits a degree of robustness to frequency variations, maintaining relatively stable ideal-fluid characteristics within a 7% bandwidth. When the frequency offset becomes too large ($\varepsilon \neq 0, \mu \neq 0$), noticeable turbulence and vortices appear.

In general, the permittivity of the medium follows Eqs. (S4) and (S5) and affect the curl and divergence of the power flow. In the system proposed in this manuscript, we construct an ENZ environment using the cutoff frequency of the waveguide. Frequency deviations from the cutoff frequency affect the dielectric constant of the medium, thus influencing the power flow. The permeability of the medium,

through doping modulation, only affects the transmittance of the system within the $\varepsilon \approx 0$ frequency band, without affecting the curl and divergence of the power flow. The presence of losses directly causes non-zero divergence in the power flow, proportional to the imaginary parts of the dielectric constant and/or permeability and can be correlated with field attenuation.

[R16] Zhou, Z., Li, Y., Li, H., Sun, W., Liberal, I., & Engheta, N. (2019). Substrate-integrated photonic doping for near-zero-index devices. Nature communications, 10(1), 4132.

[R17] Liberal, I., Lobet, M., Li, Y., & Engheta, N. (2020). Near-zero-index media as electromagnetic ideal fluids. Proceedings of the National Academy of Sciences, 117(39), 24050-24054.

Reviewer 3 states: "***3)Path Selection in Complex Scenarios: An open question regarding the "intelligence" of the escape mechanism: What happens in a maze with two possible solutions—one "easy" (shorter/direct) and one "harder" (longer/more tortuous)? Does the system prioritize the shortest path, or does energy distribute based on path impedance? Clarifying this behavior is essential for understanding the limits of the proposed "maze-solving" capability.***"

Our Response: We thank the reviewer for this valuable comment. As discussed in our analysis of multi-port systems, in NZI-mediated routing, the redistribution of electromagnetic power is primarily governed by the effective impedance of the output ports and the geometry of the branches. Each branch behaves analogously to a resistive channel in a DC circuit, with an effective resistance $R \sim a/b$, where $a$and b denote the branch length and width, respectively (Eqs. 6–9). This principle applies generally to networks with multiple branches, where the energy distribution between two or more paths follows these effective-resistance rules.

Specifically, when multiple effective paths exist, the power along each path is determined by its relative resistance. As illustrated in Figs. R16(a)–(c), when two paths have equal widths, the total power along each path is inversely proportional to its length—longer paths carry less power. Conversely, as shown in Figs. R16(d)–(f), when two paths have equal lengths, the total power along each path is proportional to its width—wider paths carry more power. In all cases, the energy remains confined to the impedance-favored routes.

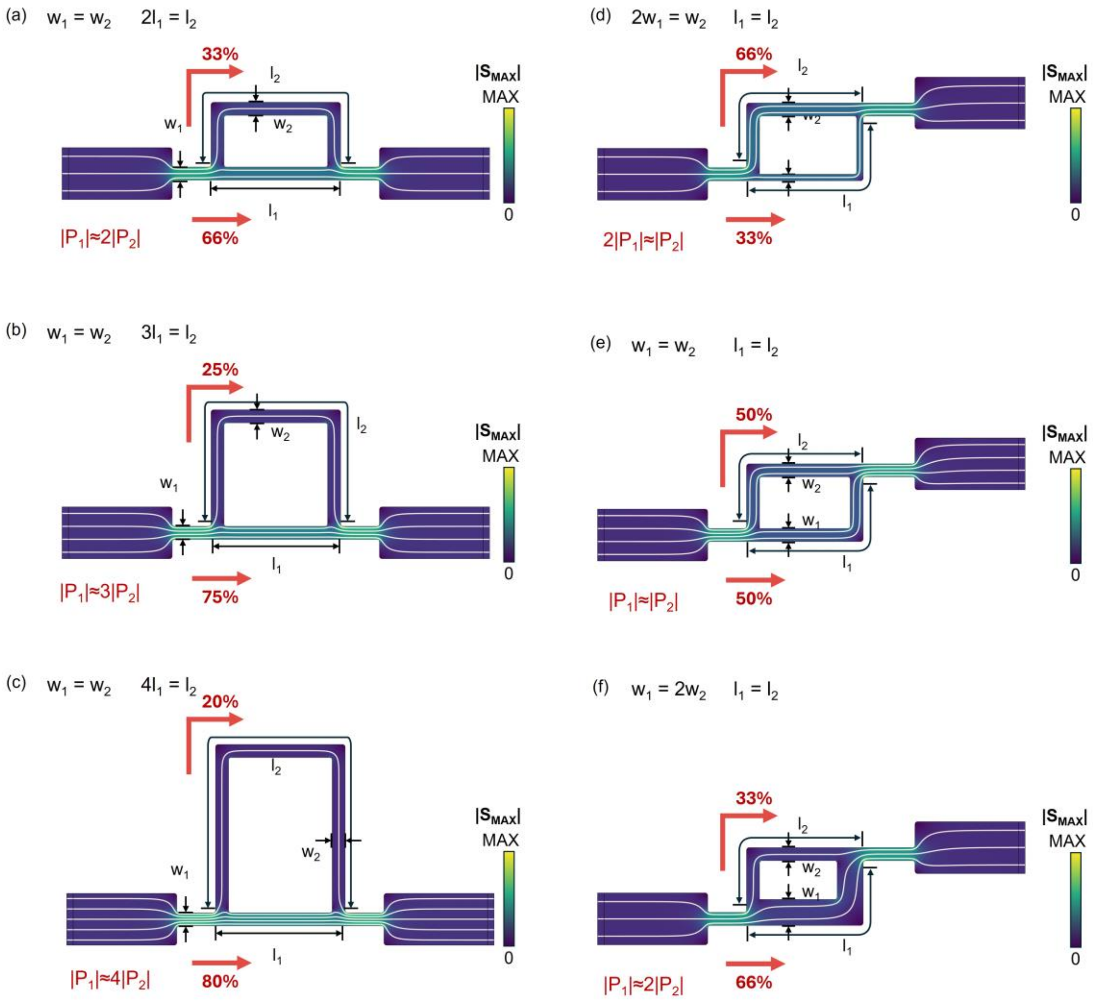

Fig. R16. Power-flow routing in EMNZ media under multi-path conditions. Two paths, labeled Channel 1 and Channel 2, are considered, with P_1 and P_2 denoting the total power transmitted along each path. (a–c) Both paths have equal widths but different lengths; the power along each path is inversely proportional to its length, with longer paths carrying less power. (d–f) Both paths have equal lengths but different widths; the power along each path is proportional to its width, with wider paths carrying more power.

Based on this comment, we have revised the descriptions in the first paragraph of **Multi-port supercoupling and bus in millimeter and terahertz frequencies** and **Supplementary Fig. S11** as followings:

"We can also control the power distribution among the output ports by adjusting their widths, effectively tuning the impedance R_s associated with the power flow at each port, as illustrated in Fig. S10. In the presence of multiple effective paths, the power distribution between the two channels also follows Eq. (9), as illustrated in Fig. S11. These results show that the Ohm's law of ideal electromagnetic power flow provides a unified framework for understanding and designing multi-port supercoupling in complex NZI structures."

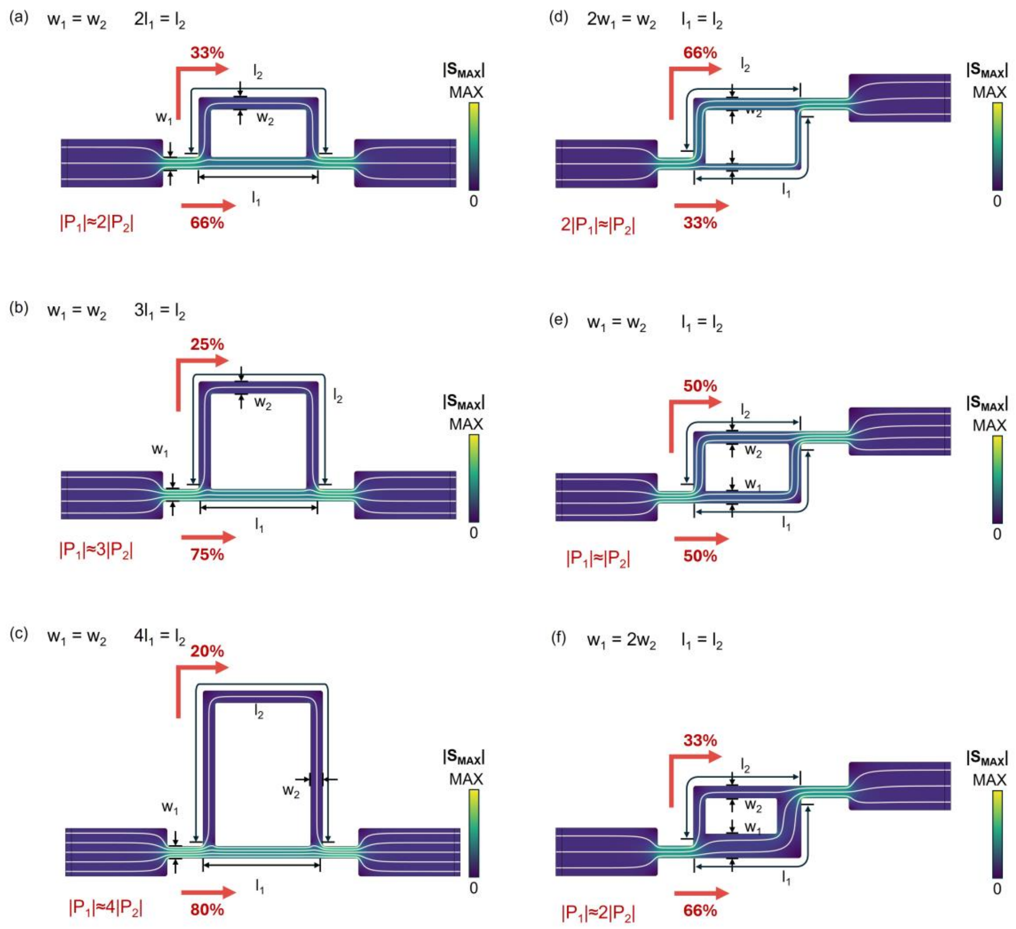

Supplementary Fig. S11 | Power-flow routing in EMNZ media under multi-path conditions. Two paths, labeled Channel 1 and Channel 2, are considered, with P_1 and P_2 denoting the total power transmitted along each path. a-c Both paths have equal widths but different lengths; the power along each path is inversely proportional to its length, with longer paths carrying less power. **d-f** Both paths have equal lengths but different widths; the power along each path is proportional to its width, with wider paths carrying more power. ”

***Reviewer 3 states*: "*4) Typographical Errors: There are minor typographical errors in the figure annotations/text (e.g., "topo" notations) that should be corrected for the final manuscript.*"**

Our Response: We appreciate the reviewers' valuable comments. We have thoroughly proofread the images and text in the manuscript. All figure captions, figure numbers, and corresponding text have been carefully checked to eliminate similar errors and ambiguities.

***Reviewer 3 states*: “Experimental Validation and Measurement (Figure 3)**
1) Data Resolution: The resolution of the experimental field scans (Figs. 3d, 3g) appears quite low compared to the simulations. Is it possible to improve the spatial resolution of the magnetic field probe measurements? Higher-resolution data would more solidly prove the correspondence between the theoretical prediction and physical reality”

Our Response: We thank the reviewer for this helpful comment. We agree that, compared with the simulation results, the spatial resolution of the original magnetic-field scans in Figs. 3(d) and (g) were relatively limited. This limitation mainly arose from the measurement-grid density adopted in the initial experiments and the finite size of the probe. To balance measurement accuracy and mechanical robustness, the probe size was set to 5 mm.

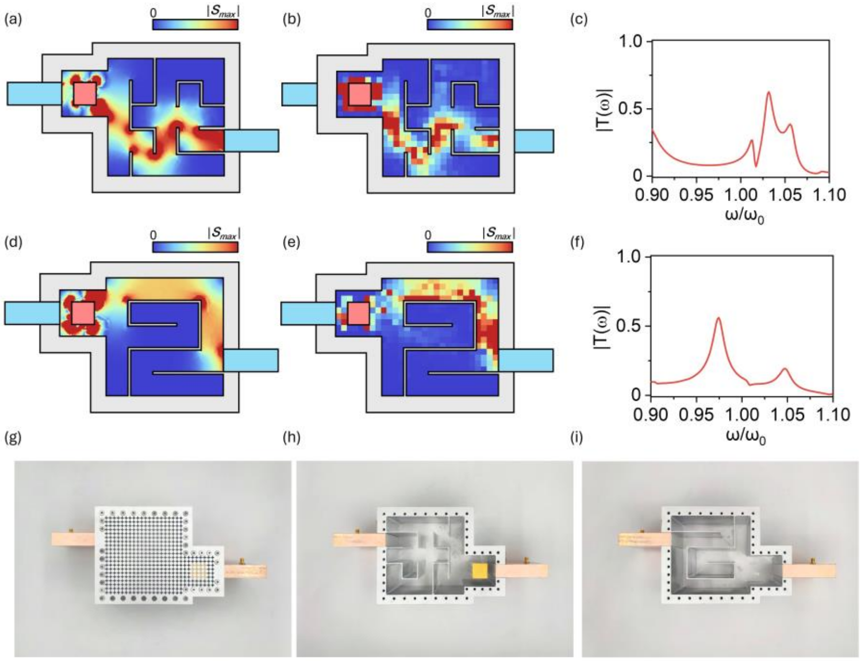

Fig. R17. (a) Simulated power-flow distribution of the high-resolution Maze 1. (b) Measured power-flow distribution of the high-resolution Maze 1. (c) Measured transmission spectrum of the high-resolution Maze 1. (d) Simulated power-flow distribution of the high-resolution Maze 2. (e) Measured power-flow distribution of the high-resolution Maze 2. (f) Measured transmission spectrum of the high-resolution Maze 2. (g) Schematic of the mesh grid used in the newly fabricated high-resolution maze platform. (h) Internal view of the newly fabricated high-resolution Maze 1. (i) Internal view of newly fabricated high-resolution Maze 2.

In response to this comment, we fabricated new maze samples with dimensions twice those of the original designs. This reduced the operating frequency by a factor of two and enabled a higher spatial resolution, as shown in Figs. R17(g)–(i). The measured transmission

spectra of the two new mazes are presented in Figs. R17(c) and (f), while the corresponding field-mapping results are shown in Figs. R17a(a)-(b) and Figs. R17a(d)-(e). These new experimental results reveal the power-flow distribution more clearly and exhibit improved agreement with the simulated field patterns. In particular, the new maze configuration provides improved measurement precision, enabling us to clearly observe the dominant propagation path together with the suppression of power flow in mismatched or dead-end branches. This further confirms the consistency among theoretical predictions, numerical simulations, and experimental observations.

Based on this comment, we have revised the descriptions in the last paragraph of **Experimental Verification** and **Supplementary Fig. S12** as followings:

"Additionally, a higher-resolution experimental result is presented in Fig. S11, which more solidly demonstrates the correspondence between the theoretical prediction and physical reality."

***Reviewer 3 states*: "2) *Dopant Uniformity: The experiment relies on a dielectric ceramic dopant to tune the effective permeability. How uniform is the effective permeability achieved by this dopant across the waveguide? Non-uniformity could explain discrepancies between simulation and experiment. Can the authors provide a map or estimate of the field uniformity?*"**

Our Response: We thank the reviewer for this important comment. In our design, dielectric ceramic dopants are introduced to tune the effective permeability of the waveguide-emulated ENZ host medium. Within the framework of photonic doping, when the area of the ENZ host region is much larger than that of the dopant, the composite system can be approximately described by a spatially uniform effective permeability, with only local perturbations in the immediate vicinity of the dopant. These features have been established in Ref. 18. Therefore, although photonic doping may introduce slight local inhomogeneity near the dopant boundaries when tuning the permeability of the ENZ medium, the overall homogeneity of the system remains well preserved. Such local perturbations do not significantly alter the global power-flow routing behavior, which is primarily governed by the near-zero-index condition and the impedance-dominated transport mechanism of the network. This conclusion is further supported by the results in Fig. R18. As shown in Fig. R18 (c,g,k,o,s), the ENZ medium exhibits the same power-flow distribution as the ideal EMNZ case in Fig. R18 (b,f,j,n,r), although the transmission drops to zero. By contrast, with photonic doping, the system recovers both the ideal-fluid-like power-flow pattern and finite transmission, as shown in Fig. R18 (d,h,l,p,t). Overall, these results indicate that

variations in permeability, including those introduced by doping, do not affect the ideal-fluid characteristics of power flow in ENZ media; rather, they mainly modify the overall transmission of the system.

We would also like to note that the remaining discrepancies between simulations and experiments are more likely caused by practical non-ideal factors, such as fabrication tolerances, slight frequency detuning from the exact EMNZ point, and limited measurement resolution, rather than by a breakdown of the effective-permeability approximation. Consistently, in our new high-resolution measurements, we observed field distributions that agree more closely with the simulations, as shown in Fig. S12.

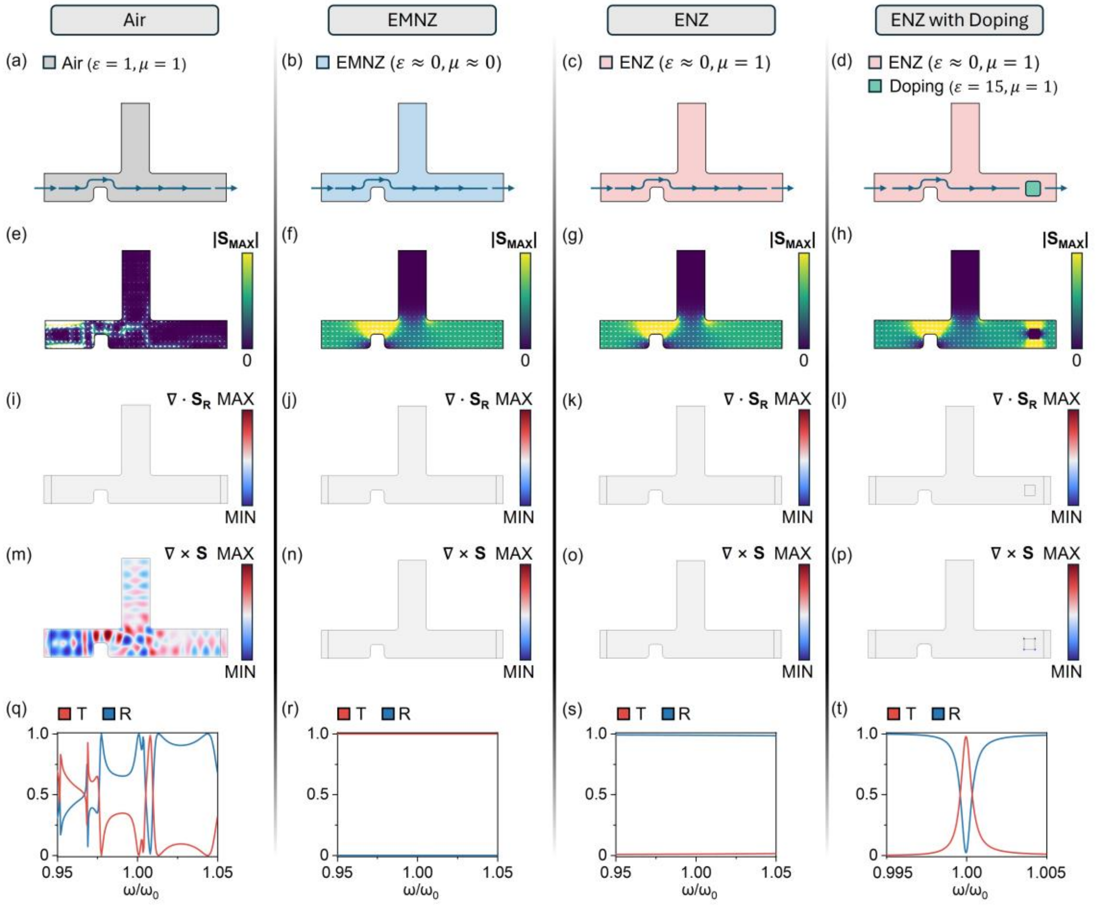

Fig. R18. Power-flow routing of electromagnetic waves in different media. (a–d) Schematic diagrams of the structures with air, EMNZ, ENZ, and ENZ with doping, respectively. (e–h) Corresponding power-flow distributions in each medium, where the white arrows indicate the direction of power flow. (i–l) Distributions of the real part of the power-flow divergence for each medium. (m–p) Distributions of the real part of the power-flow curl for each medium. (q–t) Transmission spectra of electromagnetic waves in the four media.

Based on this comment, we have revised the descriptions in the second paragraph of **Theoretical Analysis** as followings:

"Benefiting from the intrinsic field-enhancement effect of ENZ environments, embedding a square dielectric inclusion within the ENZ background enables controlled modification of the effective permeability, which can be expressed as:

$$\mu_{\text{eff}} = 1 + \frac{64 l_d^2}{\pi^4 A} \frac{\omega^2}{\omega_{\text{pmc}}{}^2 - \omega^2}, \ \omega_{\text{pmc}} = \frac{c}{\sqrt{\varepsilon_d}} \sqrt{2(\pi / l_d)^2} \quad (1)$$

where A is the area of the ENZ background, and l_d is the size of the square dopant. The frequency dependance of the effective μ_{eff} is shown in Fig. S9 according to Eq. (1). When the area of the ENZ host is much larger than that of the dopant, the interior of the composite system can be approximately treated as having a uniform effective permeability distribution."

[R18] Liberal I, Mahmoud AM, Li Y, Edwards B, Engheta N. Photonic doping of epsilon-near-zero media. *Science* **355**, 1058-1062 (2017).

***Reviewer 3 states*: "3) Probe Perturbation: The experimental results show a non-zero noise floor in regions that should be "dark." This could be induced by the measurement probe itself. Can the authors estimate the magnitude of the perturbation introduced by the magnetic field probe? Quantifying this invasiveness is important for validating the "spontaneous escape" claim.?"**

Our Response: We thank the reviewer for this important comment. In our experiment, the magnetic field is measured using an electrically small loop probe connected through a coaxial cable. As stated in the Methods section, the measured transmission coefficient from the input port to the probe is typically below –20 dB, indicating that no more than approximately 1% of the total power is coupled into the probe during measurement. Therefore, the perturbation induced by the probe is expected to be weak and does not qualitatively alter the routing behavior inside the cavity.

To further verify this point, we performed additional numerical simulations, as shown in Fig. R19. First, regarding possible leakage through the finite mesh, the transmission spectra of the two maze structures covered by a solid top plate are shown by the blue curves in Figs. R19(c) and R9d, while those of the corresponding maze structures with a mesh top plate are shown by the red curves. The results indicate that the presence of the mesh top plate has a negligible impact on the electromagnetic response of the system. To assess probe loading, small probe loops were placed at three representative locations, as illustrated in Fig. R19(e). The calculated port-response spectra are shown in Figs. R19(g)–(j), where

the energy detected by the probes remains below –30 dB, indicating that the probes do not noticeably perturb the electromagnetic fields within the mazes. Fig. R9(f) shows the cross-sectional field distribution of the maze in the waveguide EMNZ medium. The magnetic field across the section follows a cosine profile, indicating that the probe has a negligible effect on the electromagnetic field distribution within the maze.

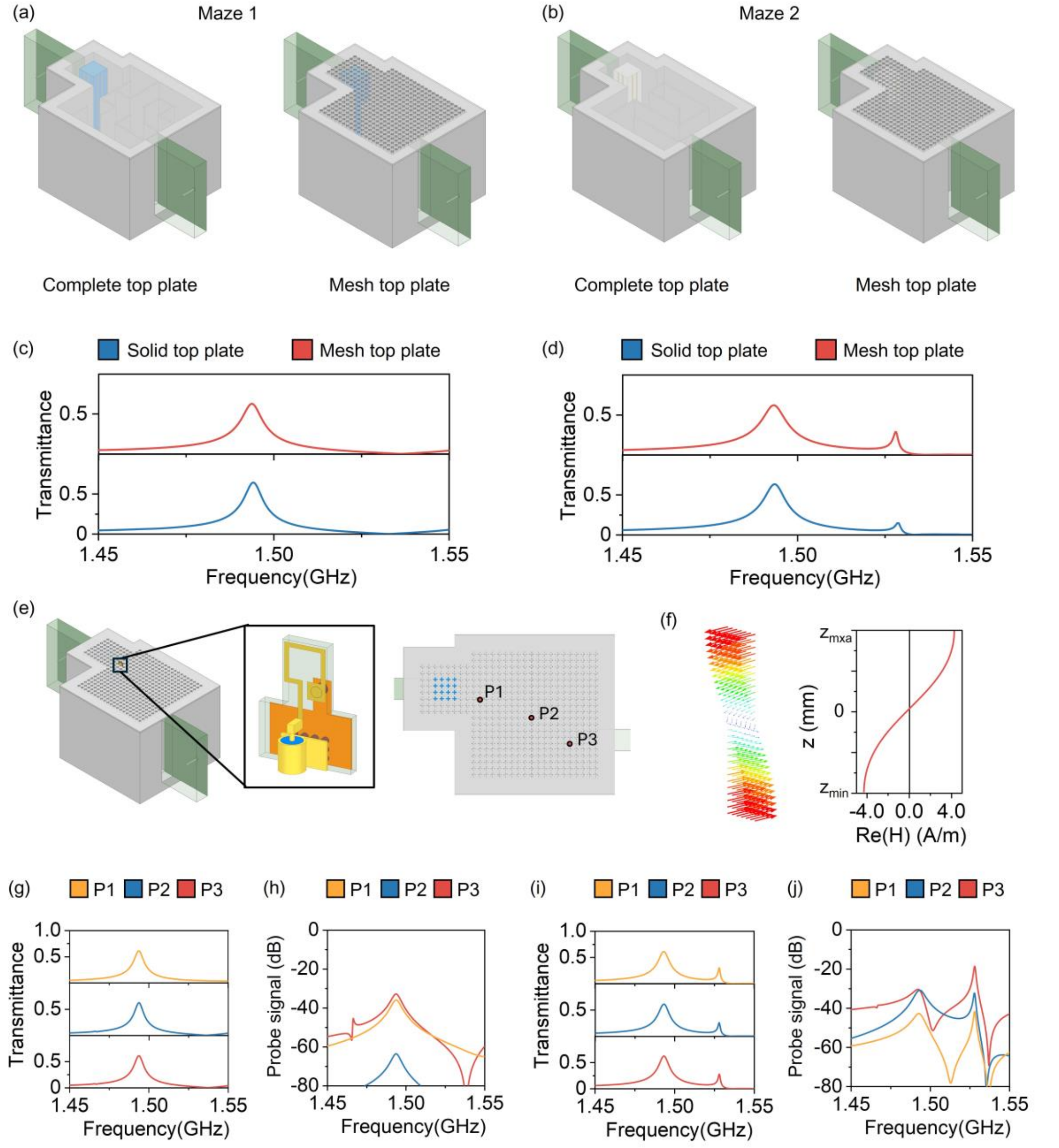

Fig. R19. Effect of test scenarios on waveguide EMNZ fields. (a–b) Schematic diagrams of the two maze configurations with a grid top plate and a solid top plate. (c) Influence of different top plates on the transmission spectrum for Maze 1. (d) Influence of different top plates on the transmission spectrum for Maze 2. (e) Schematic of probe signal detection, with three probe locations labeled P1, P2, and P3. (f) Schematic

diagram of magnetic field distribution in the cross-section of the maze. (g) Transmission spectra of the Maze 1 system for probes at P1, P2, and P3. (h) Measured probe signals at P1, P2, and P3 in Maze 1. (i) Transmission spectra of the Maze 2 system for probes at P1, P2, and P3. (j) Measured probe signals at P1, P2, and P3 in Maze 2.

We further note that the remaining discrepancies between the simulations and experiments are more likely caused by practical non-ideal factors, such as fabrication tolerances and slight frequency detuning from the exact EMNZ condition, whereas the probes have a negligible impact on the electromagnetic fields in the maze system. Consistently, in our new high-resolution measurements, we observe field distributions that agree more closely with the simulations, as shown in Fig. S12.

Based on this comment, we have revised the descriptions in the fourth paragraph of **Methods** as followings:

"The magnetic field probe of the experimental setup has a radius of 1.5 mm and is connected to one of the ports of the vector network analyzer Agilent N9971A via a 50 Ω coaxial line. The other port is connected to the input waveguide to excite the input EM waves. The probes and mesh top plate have been shown to exert a negligible impact on the fields within the maze, as illustrated in Fig. S3."

***Reviewer 3 states*:** **"Conclusion:This paper introduces a high-impact concept with strong theoretical grounding. However, addressing the questions regarding experimental fidelity (resolution, probe perturbation, material uniformity) and the robustness of the physical mechanism (sensitivity to index, multi-path behavior) is necessary to fully validate the claims. I recommend publication subject to these revisions."**

Our Response: We sincerely thank the reviewer for this thoughtful and encouraging overall assessment. We are grateful that the reviewer recognizes the conceptual significance and theoretical foundation of this work. We also fully agree that, to support the conclusions more convincingly, the manuscript should address the experimental accuracy and the robustness of the underlying physical mechanism more comprehensively.

In response, we have carefully revised the manuscript and carried out substantial additional work to address all the concerns raised by the reviewer. Specifically:

(1) Experimental resolution:

We fabricated and measured a new high-resolution maze platform and obtained updated field maps with improved spatial resolution. These new results show better agreement between experimental observations and the theoretical and simulated routing patterns.

(2) Probe perturbation:

We further clarified and quantified the invasiveness of the magnetic-field probe. As discussed in the revised manuscript, the coupling from the input to the probe is typically below –20 dB, indicating that the probe extracts no more than approximately 1% of the total power. This confirms that the probe-induced perturbation is weak and does not qualitatively affect the routing behavior.

(3) Material/dopant uniformity:

We added further discussion and analysis regarding the effective-permeability uniformity of the photonic-doping-based EMNZ platform, and clarified the validity and limitations of the effective-medium approximation adopted in our interpretation.

(4) Robustness of the physical mechanism:

We included new analyses on the sensitivity to refractive index and the EMNZ condition, as well as multi-path routing cases, which confirm that the observed routing is governed by effective impedance rather than by the geometric shortest path alone.

Overall, these new measurements, analyses, and textual revisions substantially strengthen the manuscript. We believe that the revised version now provides a much more rigorous and complete validation of both the experimental observations and the underlying physical mechanism.

We are very grateful for the reviewer's constructive suggestions, which have significantly improved the quality and clarity of the manuscript.

Our Response to the Reviewers' Comments on our Manuscnaript NCOMMS-25-88698A "Ohm's Law of Electromagnetic Ideal Fluids: Routing Light in Complex Near-Zero-Index Networks" now entitled "Ohm's Law of Electromagnetic Ideal Fluids: Impedance-Governed Supercoupling in Near-Zero-Index Networks"

Authors: *Wendi Yan, Peihang Li, Jiarui Liu, Kaifeng Li, Pengyu Fu, Shuyu Wang, Mingzhe Hu, and Yue Li*

Response to Reviewer #1:

***Reviewer 1 states*: "I thank you for their detailed response and for the revisions made to the manuscript. The changes have improved clarity in several places, particularly regarding the role of ENZ/EMNZ conditions and the experimental implementation.**

However, the central conceptual concern raised in my original report remains only partially addressed. While some terminology has been moderated, the manuscript still conveys an interpretation that may be misleading, especially for readers not deeply familiar with ENZ physics."

Our Response:

We sincerely thank the Reviewer for this important comment. We fully understand the concern that, despite our previous revisions, the manuscript may still convey a potentially misleading interpretation of the observed phenomenon. We appreciate this clarification and have carefully revised the manuscript accordingly to better align the presentation with the underlying physical picture. The specific revisions are addressed point by point in our responses below.

***Reviewer 1 states*: "The manuscript continues to describe the observed behavior using language that suggests an emergent "pathfinding" or "self-guiding" capability of electromagnetic waves.**

Even in its revised form, the narrative still implies that light:

• selects a path,

• avoids certain branches,

• or exhibits an intrinsic routing capability.

This interpretation is not physically accurate.

The observed behavior is more rigorously understood as:
a quasi-static field distribution in an ENZ/EMNZ medium, where the Poynting vector is governed by boundary conditions and impedance contrasts, and where dead-end branches do not support propagating modes.
In this regime, the field configuration is the solution of a boundary-value problem (effectively Laplacian), not the result of any dynamic or selective process."

Our Response:
We thank the Reviewer for this important clarification and fully agree with this assessment. In the revised manuscript, we have systematically reframed the presentation to avoid any wording suggestive of autonomous "**pathfinding**", "**self-guiding**", or intrinsic route selection by electromagnetic waves. The text now consistently describes the phenomenon as a passive and deterministic quasi-static power-flow distribution in an ENZ/EMNZ medium, governed by boundary conditions and effective branch impedances. The detailed revisions are addressed in our responses to the specific comments below.

Reviewer 1 states**: "*****As such, I strongly recommend that the authors:***
1. Explicitly state that the effect is not a form of "maze solving" or path selection, but a consequence of ENZ-induced quasi-static behavior."

Our Response:
We thank the Reviewer for this important suggestion and fully agree that the observed effect should not be interpreted as any form of "maze solving" or path selection, but rather as a consequence of ENZ-induced quasi-static behavior.
Specifically, to avoid anthropomorphic or misleading implications, we have removed the following expressions throughout the manuscript:

1. "**maze**", because it naturally evokes a dynamic solving process.
2. "**routing**", because it may imply spontaneous directional behavior of EM waves.
3. "**escape**", because it carries a strong anthropomorphic connotation.
4. "**avoid**", because it may suggest that EM waves actively reject certain branches, rather than being passively determined by boundary conditions.
5. "**correct/incorrect**", because correctness implicitly suggests "selection".

The manuscript has now been consistently reframed to describe the phenomenon as a quasi-static power-flow distribution governed by boundary conditions and effective branch

impedances.

Revisions are as follows:
"Routing Light" -> "Impedance-Governed Supercoupling"
"maze-like networks" -> "complex multi-branch structures/networks"
"spontaneous escape of light"/"maze solving" -> "quasi-static power-flow distribution"
"routing behavior" -> "power-flow redistribution"
"correct path" -> "Impedance-matched output"
"incorrect path" -> "PEC-terminated branch"

Reviewer 1 states*: "*2. Clarify the role of impedance in determining the observed routing, making clear that:

o branch geometry (length/width) sets effective impedance,

o the resulting field distribution is passive and deterministic."

Our Response: We thank the Reviewer for this helpful suggestion and fully agree. We have clarified that the observed power redistribution is governed by the effective impedance of each branch, which is set by its geometry, specifically the length-to-width ratio of the branch in the ideal or near-ideal ENZ/EMNZ regime. We have also made clear that the resulting field distribution is passive and deterministic. We have emphasized this point particularly in the Discussion section, where we now state as follows:

"Moreover, the effective impedance of each branch is set by its geometry, with $R_S \sim a / b$. As a result, the power-flow distribution is passively and deterministically governed by the boundary conditions and impedance contrasts across the network, in agreement with the simulated results in Fig. 2 and as further illustrated in Fig. S10 and Fig. S11."

"Inspired by the above Ohm's-law framework, the EM power-flow distribution in such systems is determined by the boundary potentials and the effective branch impedances set by $R_S \sim a / b$, thereby enabling passive control of EM power redistribution through structural design."

Reviewer 1 states*: "*3. Avoid residual anthropomorphic or suggestive terminology, even if softened."

Our Response: We thank the Reviewer for this important suggestion and fully agree. In the revised manuscript, we have systematically removed residual anthropomorphic or suggestive terminology throughout the text. Owing to the deletion of these passages and the associated reorganization of the text, the order of the references has also been updated accordingly. Specifically, the removed wording includes, among others, the following expressions and descriptions:

"This type of routing in complex networks has been extensively studied in various natural physical systems[12-15], has broad applications in areas such as autonomous driving, robotic navigation, and network routing[16-18]" -> "Deleted from the revised manuscript"

Reviewer 1 states**: "*4. Clearly state the limitations of the phenomenon, namely that:*
o it occurs only under carefully engineered ENZ/EMNZ conditions,
*o it is not representative of general electromagnetic propagation."***

Our Response: We thank the Reviewer for this important suggestion and fully agree. In the revised manuscript, we now state more explicitly that the reported phenomenon occurs only under carefully engineered ENZ/EMNZ conditions and should not be interpreted as representative of general electromagnetic propagation. Instead, it is a specific consequence of the near-zero-index regime considered in this work. Revisions are as follows:

In **Discussion** section:
"However, it should be emphasized that this phenomenon arises only under carefully engineered ENZ/EMNZ conditions and is not representative of general EM propagation. In the present platform, because both the permittivity and permeability are dispersive, this effect can only be observed in a narrowband structure. In fact, the corresponding bandwidth is about 7%, as shown in Fig. S9."
"In addition, although the above theoretical derivation was carried out for the EMNZ case, the underlying mechanism is in fact highly robust against variations in permeability. It therefore applies to a broad range of ENZ implementations, including the waveguide-emulated plasmonic used in our experiments. However, for conventional media, where the assumptions underlying ideal EM power flow no longer hold, the Ohm's-law description is generally no longer applicable."

In addition, we have carefully considered whether the term "Ohm's law of ideal EM fluids" might itself be open to misinterpretation. Given that Iñigo et al. [R1] already predicted that

ideal EM-fluid-like behavior arises only in ENZ/EMNZ media, we believe that the phrase "ideal EM fluids" already carries this ENZ/EMNZ assumption. On this basis, we have chosen to retain the term for the present work. At the same time, we now use it with greater caution and explicitly confine it to the ENZ/EMNZ regime throughout the revised manuscript.

[R1] Liberal, I., Lobet, M., Li, Y. & Engheta, N. Near-zero-index media as electromagnetic ideal fluids. *Proc. Natl. Acad. Sci.* **117**, 24050-24054 (2020).

Reviewer 1 states*: "*About Novelty, the revised manuscript still presents the results in a way that may suggest a fundamentally new physical mechanism. I encourage the authors to more clearly position their contribution as:

• an application and visualization of known ENZ supercoupling and quasi-static transport,

rather than:

• a fundamentally new paradigm in wave propagation."

Our Response: We thank the Reviewer for this important comment and fully agree that the novelty of the work should be positioned more carefully. In the revised manuscript, we have therefore reframed the presentation to avoid suggesting that the reported results constitute a fundamentally new wave-propagation mechanism. Instead, we now position our contribution as an extension, application, and direct visualization of known ENZ supercoupling and quasi-static transport in complex multi-branch networks.

More specifically, the revised manuscript emphasizes that the main advances of this work are: (i) extending the supercoupling picture from conventional point-to-point channels to complex multi-port structures, (ii) directly visualizing the corresponding power-flow distribution experimentally, and (iii) providing an effective Ohm-like framework for describing such impedance-governed redistribution under ENZ/EMNZ conditions. This revised positioning is now reflected throughout the title, Abstract, Introduction, Discussion, and Conclusion.

Revisions in **Abstract** section:

"In this work, we extend NZI supercoupling to complex, multi-branch EM structures, where the EM power flow follows a passive, determined, and quasi-static distribution governed by boundary conditions and impedance contrasts, while PEC-terminated branches do not carry propagating power flow."

"This quasi-static power-flow distribution can be described by an "Ohm's law of ideal EM power flow", in which the potential is determined by the boundary conditions, while the effective impedance is set by the length-to-width ratio of each branch. "
"Beyond the physical interpretation, this multi-branch supercoupling suggests a possible route toward multi-port EM interconnect architectures, with the desired interconnection determined by the design of boundary conditions and branch impedances."

Revisions in **Introduction** section:
"In this work, we extend NZI supercoupling from isolated channels to complex, multi-branch EM networks, and systematically investigate how EM power is distributed through boundary-conditioned and impedance-governed transport."
"This multi-port supercoupling behavior is directly visualized experimentally using a waveguide-emulated plasmonic platform[28,29] combined with photonic doping[14,15]."
"Motivated by analogies with pressure-driven water flow in branched hydraulic networks[21] and current distribution in direct-current circuits, we derive an Ohm's law of ideal EM power flow[19,20], which provides a simple framework for explaining the observed quasi-static power-flow distribution in NZI media."

Revisions in **Conclusion** section:
"In this work, extend NZI supercoupling form conventional point-to-point transport to complex multi-branch structures, and investigate the resulting quasi-static EM power-flow redistribution through an analogy to pressure-driven flow in fluidic networks. Specifically, EM power injected into complex multi-branch NZI structures is passively redistributed toward impedance-matched output ports, while power flow into strongly mismatched or PEC-terminated branches is strongly suppressed. Both simulations and experiments verify and directly visualize this multi-branch power-flow distribution. The behavior can be described by an Ohm's law of ideal EM fluids, in which the Poynting-vector potential difference between ports and the effective impedance width-to-length ratio determines the power-flow intensity, providing a predictive description of passive and deterministic EM power redistribution in complex NZI structures."

Reviewer 1 states*: "*In conclusion, the work is technically sound and experimentally well executed. However, further clarification and reframing of the physical interpretation are necessary to avoid misleading conclusions and to ensure proper contextualization within the ENZ literature.
For these reasons, I still recommend major revision."

Our Response: We sincerely thank the Reviewer for the positive assessment of the technical quality and experimental execution of our work. We also fully appreciate the Reviewer's concern regarding the need for clearer physical interpretation and more careful positioning within the ENZ literature. Following this valuable guidance, we have comprehensively revised the manuscript to clarify the physical picture, remove potentially misleading wording, and more appropriately frame the work as an extension, application, and visualization of known ENZ supercoupling and quasi-static transport in complex multi-branch networks. For clarity, we have provided a detailed table summarizing the revisions made throughout the main text. We hope that these revisions have adequately addressed the Reviewer's concerns.

Table R1. Representative wording revisions made throughout the manuscript

Section	Original Wording	Revised Wording
Title	Routing Light	Impedance-Governed Supercoupling
Abstract	a highly directional redistribution of EM energy toward output ports impedance-matched to the input	a passive, determined, and quasi-static distribution governed by boundary conditions and impedance contrasts
	PEC-terminated branches is effectively suppressed	PEC-terminated branches do not carry propagating power flow
	a reconfigurable multi-port EM bus architecture	a possible approach toward multi-port EM interconnect architectures
	plug-and-play integration	on-chip interconnects
Fig. 1	maze	Complex branched channel network
	spontaneous escape of light	quasi-static power flow distribution
Introduction	a distinctive electromagnetic (EM) transport mechanism	a characteristic feature of electromagnetic (EM) wave propagation
	investigate how EM power is distributed and routed in complex NZI structures	investigate how EM power is distributed across interconnected NZI branches.
	when water is injected into the entrance of a complex maze, it flows under gravity, spontaneously finds the shortest path, and naturally avoids the wrong turns	when water is injected into a complex branched channel network, it propagates from the inlet to the outlet through a connected branch, while almost no water enters the blocked branches
	Routed	Governed
	maze-like structures	complex multi-branch PEC-defined structures
	this multi-port routing behavior	this multi-port supercoupling behavior
Results	NZI mazes	NZI networks
	EM power is preferentially transported	power flow is predominantly

	along a dominant route	concentrated in the impedance-favored branches
	pressure-driven flow redistribution in fluidic mazes	pressure-driven flow redistribution in fluidic networks
	boundary-conditioned routing behavior	boundary-conditioned redistribution behavior
Discussion	impedance-matched transport path	impedance-matched transport branch
	correct path	Impedance-matched output
	incorrect path	PEC-terminated branch
	spontaneous escapes	quasi-static power-flow distributions
	EM energy preferentially propagates toward impedance-matched exits	EM power is passively redistributed toward impedance-matched output ports
	Multi-port supercoupling and bus in millimeter and terahertz frequencies	Multi-port interconnect applications enabled by multi-branch supercoupling
	allows dynamic interconnection … for precise wavefront control	could enable reconfigurable coupling … to form arrays for wavefront shaping
Conclusion	NZI-filled maze-like networks	complex multi-branch NZI structures
	validate this striking behavior	verify and directly visualize this multi-branch power-flow distribution
	guiding EM power redistribution	predictive description of passive and deterministic EM power redistribution
	electromagnetic-fluid-like transport	quasi-static EM power-flow distribution

Response to Reviewer #2:

Reviewer 2 states*: *"I have read the revised version of the manuscript, and the authors' responses to the three referees.

From my perpsective, the authors have done a good job in carefully addressing all of the referees' questions, and have suitably 'reflected' these amendments in the revised manuscript.

Although there are still some doubts as to the novelty of the presented concept (as indicated by the first referee, too), I am satisfied that the revised work is a solid advance, now (even marginally) meriting publication in NCOMMs, as is."

Our Response: We sincerely thank the Reviewer for this very positive assessment of our revised manuscript and for recognizing our efforts in addressing the referees' comments. We are also grateful for the Reviewer's thoughtful perspective on the novelty of the work.

Following the reviewers' suggestions, we have further clarified the positioning and physical interpretation in the revised manuscript.

Response to Reviewer #3:

Reviewer 2 states*: *"I thank the authors for their careful and substantial revisions. They have addressed my previous concerns well, and I find the revised manuscript significantly improved in both clarity and technical support. In particular, the additional explanations, simulations, and experimental clarifications have satisfactorily resolved the main issues I raised.

Overall, I am now supportive of publication.

I only noticed one minor issue: in Fig. R12, the colorbar labeling appears to be reversed. If this figure, or a related version of it, is to be included in the main text or supplementary materials, I suggest that the authors correct the colorbar annotation to avoid possible confusion.

Other than this small point, I have no further concerns."

Our Response: We sincerely thank the Reviewer for the careful reading of our revised manuscript and for the very positive assessment. We are glad that the additional explanations, simulations, and experimental clarifications have satisfactorily resolved the Reviewer's previous concerns. We also appreciate the Reviewer for pointing out the minor issue in Fig. R12. Although this figure does not appear in the main text or Supplementary Information, we have corrected the colorbar labeling accordingly for completeness. The corrected figure is provided below.

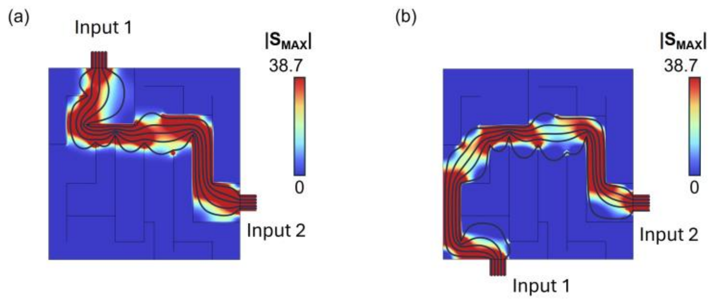

Fig. R12. Power flow streamline diagram of the maze in the manuscript, where the density of the streamlines represents the magnitude of the power flow.
